# National-level assessment of infrastructure-coupled roadside solar energy toward transportation decarbonization in China

**DOI:** 10.1038/s41467-026-73872-w

**Published:** 2026-06-02

**Authors:** Zhaoyuan Wu, Jianxiao Wang, Lanyi Wei, Bo Li, Lin Chen, Lu Zhang, Daniel M. Kammen, Gengyin Li, Ming Zhou, Jie Song

**Affiliations:** 1 State Key Laboratory of Alternate Electrical Power System with Renewable Energy Sources, North China Electric Power University, Beijing, China; 2 National Engineering Laboratory for Big Data Analysis and Applications, Peking University, Beijing, China; 3 School of Electrical Engineering, Guangxi University, Nanning, Guangxi Zhuang Autonomous Region China; 4 Institute for Interdisciplinary Information Sciences, Tsinghua University, Beijing, China; 5 College of Information and Electrical Engineering, China Agricultural University, Beijing, China; 6 Energy and Resources Group, University of California Berkeley, Berkeley, CA USA; 7 School of Advanced Manufacturing and Robotics, Peking University, Beijing, China

**Keywords:** Energy science and technology, Energy and society

## Abstract

Decarbonizing transportation requires approaches that embed renewable generation into existing infrastructure. Here we show that roadside photovoltaic deployment along China’s roads and railways can be quantified using a geospatial framework that links segmented transport corridors to meteorological grids. The approach maps 480,019 km of transport infrastructure to 4,133 meteorological grids and provides a scalable alternative to coarse regional averaging. Across all deployment scenarios, roadside photovoltaic systems could support 40.91-202.84 GW of installed capacity and generate 56.6-239.2 TWh of electricity annually. Under the baseline scenario, annual generation reaches about 100.6 TWh, equivalent to about 50% of current transport-sector electricity demand. The resulting carbon reduction reaches 33.62-143.97 Mt CO_2_ annually. The results reveal strong regional heterogeneity, with North and Central China showing the highest near-term potential, while Northwest China could act as a generation-export region. These findings provide a basis for region-specific infrastructure planning and more coordinated transport-energy system integration.

## Abstract (web summary)

This study quantifies infrastructure-coupled roadside solar potential across mainland China by linking transport corridors to meteorological grids and assessing electricity generation, carbon mitigation and grid-flexibility effects.

## Introduction

The integration of renewable energy into land-based transportation systems has emerged as a strategic lever to address pressing global challenges, including climate change, resource scarcity, and air pollution^1–3^. Transportation corridors, which extend across vast geographic and climatic zones, offer not only logistical connectivity but also untapped spatial resources for distributed energy deployment. Several countries have already initiated such integration: the United States has piloted photovoltaic (PV)-powered electric vehicle charging stations along interstate highways^4–6^; Germany has installed PV panels on sound barriers and rail-side embankments^7^; and France has experimented with solar roadways and station rooftops. These international efforts underscore the growing recognition of transportation infrastructure as a viable platform for solar energy generation^8^. In China, the scale of opportunity is particularly pronounced^9,10^. As of 2024, the national transportation network encompassed 5.44 million kilometers of roads, including 183,600 kilometers of highways, and 159,000 kilometers of railways, including 45,000 kilometers of high-speed lines^11^. A wide array of spaces, such as roadside safety zones, service areas, tunnels, bridges, and rail-side platforms, can be utilized for PV deployment^12^. These distributed systems enable localized power generation, reduce grid expansion needs, and support energy demands for both traction and non-traction loads, including electric vehicle charging, station lighting, and, in cold regions, road heating for de-icing^13^. Such synergies between mobility and clean energy infrastructure lay a promising foundation for China’s low-carbon transportation transition.

Currently, the integration of renewable energy in land-based transportation systems primarily centers on the deployment of PV systems along roadside corridors. This approach capitalizes on underutilized land adjacent to transportation infrastructure, seamlessly integrating PV generation with existing facilities to enhance spatial efficiency. While distributed PV potential has been rigorously assessed across spatial scales, from urban to national and global levels, existing research predominantly focuses on rooftop PV installations^14–16^. For example, Europe’s Solar Atlas project and the distributed PV potential analysis platform developed by the U.S. National Renewable Energy Laboratory (NREL) employ refined modeling to evaluate regional PV development potential and economic feasibility^17,18^. In China, regional PV potential models have recently been established, including quantitative analyses of rooftop PV potential in urban areas and estimations of their associated carbon reduction benefits^19,20^. This bias stems not only from technical and structural constraints but also from institutional and sociopolitical factors, including preferential policies, urban-centric energy planning, and public engagement in visible urban projects. In contrast, the deployment of PV systems along transportation corridors remains underexplored, owing to spatial complexities and technical uncertainties, and more critically, the systemic exclusion of transportation infrastructure from conventional energy planning paradigms. By redefining transportation corridors as active contributors rather than passive components of national decarbonization strategies, this study aims to bridge this strategic oversight, providing a systematic evaluation of an underexplored yet high-potential renewable energy frontier.

Despite growing interest in integrating solar energy with transportation infrastructure, quantifying roadside PV potential across a national-scale transportation system remains methodologically demanding because estimates of installable capacity, generation output and carbon reduction need to be aligned across extensive linear transport corridors and heterogeneous meteorological conditions. Previous work has quantified the photovoltaic potential of highway surfaces in China, providing an important basis for transport-infrastructure solar assessment^21^. However, extending this perspective from highway-surface applications to infrastructure-coupled roadside solar across both roads and railways requires coordinated representation of corridor geometry, deployable roadside area, spatially matched meteorological conditions, generation profiles, carbon mitigation potential and power-system effects. These requirements arise from the technical and computational complexity of such evaluations. Unlike rooftop or urban PV assessments, transportation networks are linear, discontinuous and spatially extensive, often traversing diverse geographic and climatic zones. Accurate estimation requires the alignment of meteorological data with the detailed geometry of roads and railways, which is a nontrivial task. For example, considering that China’s road network spans more than 5.4 million kilometers, matching each 1-km road segment to a national gridded meteorological dataset would involve tens of millions of spatial queries and hourly data computations. Traditional province-level averaging methods may misestimate the PV potential in regions with steep spatial gradients in solar irradiance or terrain conditions. Therefore, developing a scalable methodology that captures spatial heterogeneity while accurately quantifying carbon reduction potential remains an open and critical challenge.

Here, we show that an infrastructure-aware assessment can quantify the nationwide deployable capacity, electricity generation, and carbon mitigation potential of roadside solar systems along China’s transportation corridors. By coupling segmented transport networks from OpenStreetMap with meteorological data from 4133 grid points, the framework resolves the spatial heterogeneity that is missed by province-level averaging. The results indicate 40.91–202.84 GW of deployable capacity and 56.6–239.2 TWh of annual generation across scenarios, enough to meet about half of current transport-sector electricity demand and reduce emissions by 33.62–143.97 Mt annually. Furthermore, the study analyses the mismatch between regional transportation energy demand and PV energy potential and highlights the importance of regionalized energy integration planning. It also evaluates the compatibility of newly deployed distributed PV systems with existing load profiles and provides a scientific basis for prioritizing energy integration strategies across regions.

## Results

### National geospatial framework for roadside solar

This study develops a comprehensive assessment framework for evaluating the energy potential of China’s land transportation system. The quantitative analysis focuses on mainland China, excluding Taiwan, Hong Kong and Macau. On the basis of nationwide road and meteorological data, the framework integrates data cleaning, matching, and multi-scenario analysis to comprehensively uncover the PV resource potential and its regional disparities. First, road network data for roads and highways were obtained from OpenStreetMap^22^. After preliminary cleaning, seven types of inapplicable road data, including bridge, tunnel, bicycle lane, and light rail data, were excluded, leaving only usable datasets for roads and highways. The retained datasets include 101,055 km of railways, 134,162 km of highways, 130,567 km of arterial roads, and 114,235 km of other roads, totaling 480019 km. To facilitate regional analysis, China was divided into six regions, Northeast China (NE), North China (N), Northwest China (NW), East China (E), Central China (C), and South China (S), on the basis of the power grid zoning methodology. This classification reflects the operational boundaries commonly used in national power system planning.

**Fig. 1:**
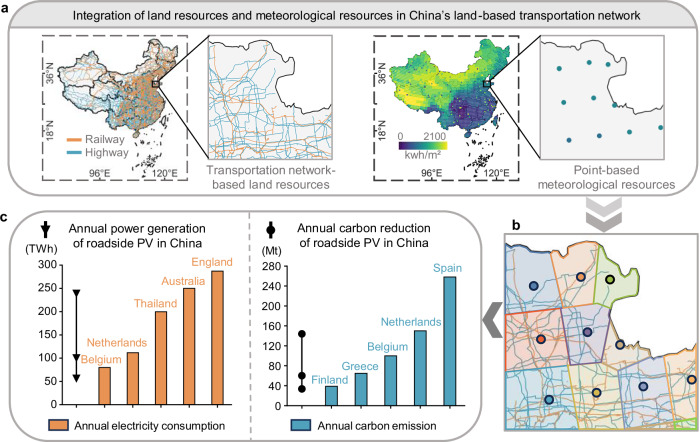
Methodology and preliminary results of geospatial matching between meteorological data and transportation system layouts. **a** Nationwide extraction and spatial integration of transportation-network-based land resources and point-based meteorological resources in China. Orange lines denote railways, blue lines denote highways, and the color scale indicates annual solar irradiation based on meteorological station data. **b** Enlarged view of the local matching between transportation corridors and nearby meteorological observation points. **c** Comparison of the estimated annual electricity generation and carbon mitigation potential of roadside photovoltaic deployment in China with the annual electricity consumption and annual carbon emissions of selected countries. Orange bars indicate annual electricity consumption, blue bars indicate annual carbon emissions, and black markers indicate the estimated roadside PV potential under optimistic (high-efficiency modules with dense deployment), moderate (medium efficiency and deployment density) and conservative (low-efficiency modules with sparse deployment) deployment scenarios. PV photovoltaic. Map outlines were generated from Natural Earth public-domain data, and transportation network data were derived from OpenStreetMap contributors under the Open Database License. The map shows Taiwan, Hong Kong and Macau for geographic context, whereas the quantitative analysis is restricted to mainland China. Maps were drawn and modified by the authors in QGIS v.3.32.0.

In the data-matching process, as illustrated in Fig. 1, segmented road network data were matched with gridded meteorological data across 4133 grid points nationwide^23^. With the use of a longitude‒latitude-to-distance transformation method, key road points were aligned with the meteorological grid to ensure spatial and temporal consistency in the PV potential assessment. The matching procedure assigned solar irradiance and other hourly meteorological variables from the nearest grid point to each road segment, providing spatially matched inputs for estimating the electricity generation potential of roadside PV systems. By analysing combinations of different scenarios, this study comprehensively determined the regional disparities and technical sensitivities of roadside PV resources. Building on this, the potential contribution of roadside PV to carbon emission reduction was calculated using regional carbon emission factors and standard coal consumption data. Figure 1c presents the preliminary results and compares the electricity generation potential and carbon reduction capacity with those of other major countries.

### Spatiotemporal distribution of roadside PV deployment potential

**Fig. 2:**
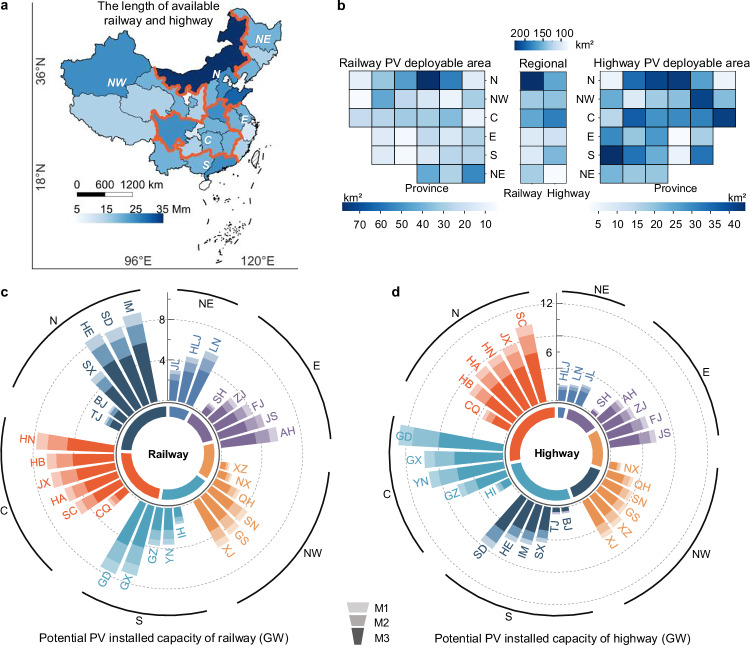
Spatial and technical distribution of roadside PV deployment potential in various scenarios. **a** Total road network length across six major regions of China, highlighting regional disparities. The red solid lines denote regional boundaries, and the blue color scale indicates the total available railway and highway length in each province. **b** Deployable roadside PV area under a moderate deployment scenario at provincial and regional scales. The central columns represent total deployable areas by region; the left and right columns show provincial deployable areas for railways and highways, respectively. **c**, **d** Potential PV installed capacity along railways (**c**) and highways (d) under three deployment scenarios (M1–M3). The inner rings indicate total regional capacities, whereas the radial bar charts show provincial potentials, with light to dark shading denoting M1, M2 and M3, respectively. The bars reflect the range between the maximum and minimum potential capacities across scenarios. M1, dual-axis tracking with conservative deployment and 20% module efficiency; M2, tilted single-axis tracking with moderate deployment and 24% module efficiency; M3, fixed-tilt deployment with optimistic deployment and 28% module efficiency; E east; NE northeast; N north; NW northwest; S south; C central. PV photovoltaic. AH Anhui; BJ Beijing; CQ Chongqing; FJ Fujian; GD Guangdong; GS Gansu; GX Guangxi; GZ Guizhou; HA Henan; HB Hubei; HE Hebei; HI Hainan; HLJ Heilongjiang; HN Hunan; IM Inner Mongolia; JL Jilin; JS Jiangsu; JX Jiangxi; LN Liaoning; NX Ningxia; QH Qinghai; SC Sichuan; SD Shandong; SH Shanghai; SN Shaanxi; SX Shanxi; TJ Tianjin; XJ Xinjiang; XZ Xizang; YN Yunnan; ZJ Zhejiang. The map was drawn and modified by the authors in QGIS v.3.32.0, and map outlines were generated from Natural Earth public-domain data. The map shows Taiwan, Hong Kong and Macau for geographic context, whereas the quantitative analysis is restricted to mainland China.

The energy potential of land transportation systems refers to the capacity to develop renewable energy along railway and highway networks by deploying distributed PV systems. This involves assessing the PV installation capacity and generation potential of road systems across different regions and considering regional resource conditions and technical parameters to determine energy utilization efficiency. Figure 2 illustrates the spatial distribution of energy potential within China’s land transportation system.

China’s roadside PV deployment potential exhibits pronounced spatial and technical heterogeneity, shaped by the interplay among infrastructure density, solar irradiance, and deployment configurations. As shown in Fig. 2, North China and Central China possess the highest absolute installable capacities for both highways and railways, driven by their dense transportation infrastructure—particularly in provinces such as Henan, Hebei, and Shandong. However, the southern region, despite having a shorter total road length, demonstrates disproportionately high PV installation potential due to its favorable solar irradiance and compact transportation corridors, which make it well-suited for high-density distributed PV applications.

Technical parameters, including module efficiency and system configuration, further influence regional deployment prospects. In the optimistic scenario—fixed-tilt structures with 28% efficiency—the installed capacity reaches 202.84 GW nationally, nearly five times that of the minimum scenario using dual-axis tracking and 20% efficiency. Central China and North China consistently perform best across all the scenarios, whereas Northwest China benefits more from land availability than from solar intensity. This underscores the importance of matching deployment strategies to regional conditions: centralized rail-side PV may be prioritized in North China and Northwest China, whereas highway-based distributed systems may be more effective in Central China and South China.

**Fig. 3:**
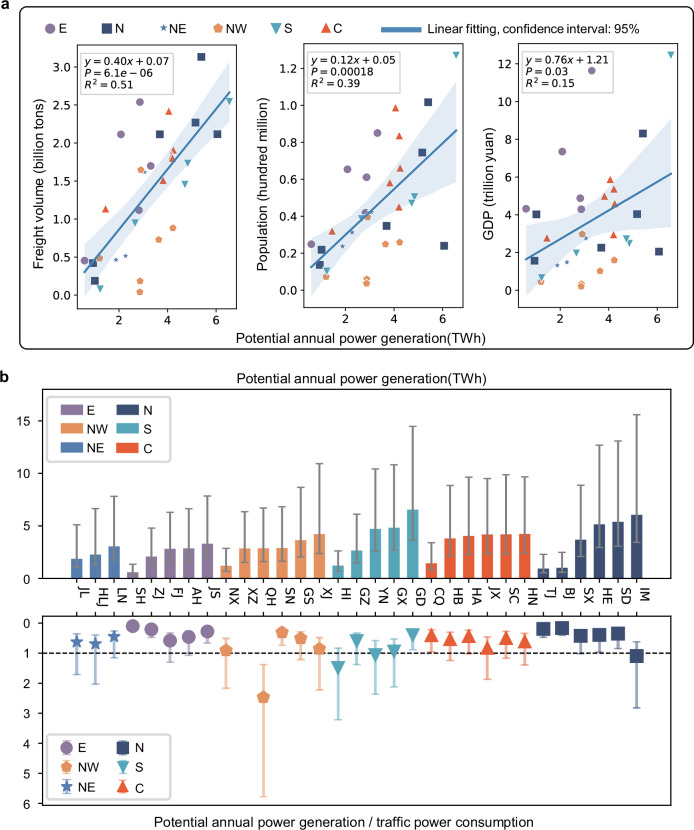
Roadside PV generation potential and key factors in China’s land transportation system. **a** Correlations between roadside PV generation potential and socioeconomic indicators across 31 provinces in China. Symbols and colors denote the six regions. Blue lines indicate linear fits across all provinces, shaded areas indicate 95% confidence intervals, and *P*-values and R² values are obtained from simple linear regression. **b** Absolute PV generation potential and its ratio to annual electricity consumption in the transportation sector across provinces. The bars and scatter points reflect the results in the baseline scenario (M2), whereas the error bars represent the full range (maximum to minimum) of outcomes across all 63 deployment scenarios, capturing the underlying uncertainty. M2, tilted single-axis tracking with moderate deployment and 24% module efficiency; E east; NE northeast; N north; NW northwest; S south; C central. PV photovoltaic; GDP gross domestic product; AH Anhui; BJ Beijing; CQ Chongqing; FJ Fujian; GD Guangdong; GS Gansu; GX Guangxi; GZ Guizhou; HA Henan; HB Hubei; HE Hebei; HI Hainan; HLJ Heilongjiang; HN Hunan; IM Inner Mongolia; JL Jilin; JS Jiangsu; JX Jiangxi; LN Liaoning; NX Ningxia; QH Qinghai; SC Sichuan; SD Shandong; SH Shanghai; SN Shaanxi; SX Shanxi; TJ Tianjin; XJ Xinjiang; XZ Xizang; YN Yunnan; ZJ Zhejiang.

These findings suggest that optimizing China’s roadside PV deployment requires consideration of both the physical infrastructure layout and scenario-specific technical assumptions rather than a sole reliance on road length or irradiance levels. Nationally, the PV installation potential in the baseline scenario (75.22 GW) is comparable to Japan’s current total PV capacity, and the optimistic scenario approaches the scale of Europe’s installations, underscoring the strategic significance of transport-integrated solar systems in national energy planning. To further reveal regional heterogeneity and deployment opportunities, Fig.3 summarizes the roadside PV generation potential and key factors in China’s land transportation system.

Roadside PV generation potential and transportation electricity demand exhibit distinct spatial patterns, resulting in regionally differentiated deployment opportunities. As shown in the correlation patterns derived from Fig. 3a, North China and Central China demonstrate strong alignment between PV potential and freight volume, which reflects the colocation of extensive transportation infrastructure and solar resource availability. In contrast, eastern and southern provinces, such as Jiangsu and Guangdong, present high PV potential, driven largely by their dense populations and elevated transportation activity, despite facing land use constraints that limit deployment scale. The relationship between PV potential and gross domestic product (GDP) is less consistent across regions. High-GDP provinces typically exhibit convergence of infrastructure and electricity demand. By contrast, Xinjiang and Qinghai in Northwest China show considerable roadside PV potential, driven mainly by resource endowments such as available land and favorable solar irradiance rather than by economic development.

The alignment between PV generation potential and actual electricity demand in the transportation sector further illustrates spatial imbalances that affect system planning. In North China and Central China, high generation capacity coincides with substantial transportation electricity consumption, making these regions suitable for balanced, high-utilization deployment. Conversely, in Northwest China and Northeast China, the generation potential exceeds the local demand, which indicates that these areas could serve as energy exporters or flexible power sources supporting broader grid needs. In contrast, provinces in East China and South China, although rich in generation potential, experience demand levels that exceed locally deployable PV capacity, which suggests the need for storage integration and external power balancing.

These results imply a functional classification of regions on the basis of their supply‒demand balance. North China and Central China emerge as zones demonstrating generation−demand matching, where rapid PV deployment could be directly utilized. Northwest China and Northeast China can be positioned as generation−surplus regions that support interregional balancing or serve electrified transportation corridors with flexible supply. Eastern and southern provinces, with their high demand but limited capacity, require integrated solutions that combine PV with energy storage, grid enhancements, or demand-side management. Recognizing these regional dynamics is essential for designing a transport‒energy transition strategy that is not only technically efficient but also spatially coordinated. However, the credibility of such region-specific assessments depends heavily on the underlying spatial resolution of the estimation method. Most conventional approaches rely on the province-level aggregation of irradiance and infrastructure statistics, which may fail to capture the fine-grained heterogeneity revealed in our spatial analysis. To examine how estimation accuracy varies across methodologies, we next compare the proposed spatial matching technique with a baseline province-level averaging approach.

### Comparative analysis of roadside PV potential evaluation methods

**Fig. 4:**
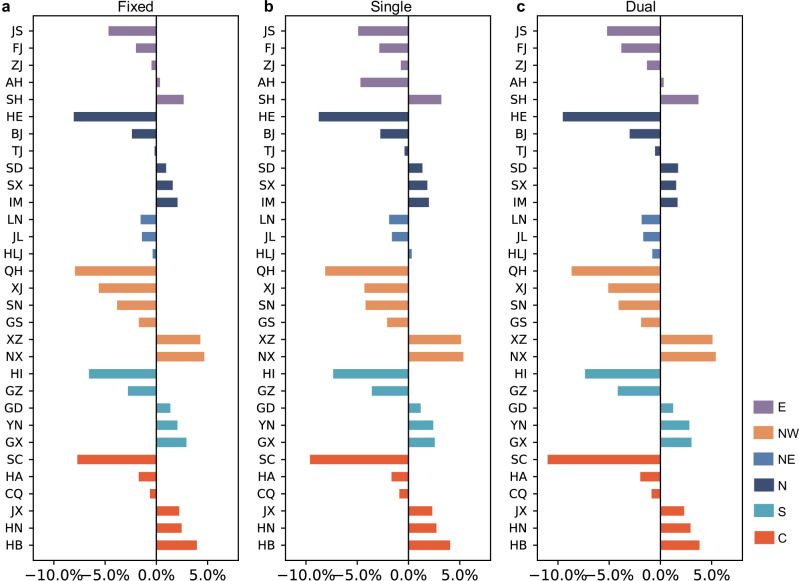
Provincial estimation bias of roadside PV generation under different spatial resolution assumptions. **a–c** Relative error in provincial roadside PV power generation estimated using the conventional province-level method relative to the proposed spatial matching approach under fixed-tilt (**a**), single-axis tracking (**b**) and dual-axis tracking (**c**) configurations. The relative error is calculated as (estimated - reference) / reference, where the reference scenario adopts the proposed spatial matching approach and the estimated scenario uses the conventional province-level method. In the restimated scenario, the provincial capital city obtained from the Gaode Map API (2023) is used as a fixed reference point; The nearest meteorological grid to this capital point is used as the representative climatic condition. Positive and negative values indicate overestimation and underestimation, respectively. Colors denote the six regions. E east; NE northeast; N north; NW northwest; S south; C central. PV photovoltaic; AH Anhui; BJ Beijing; CQ Chongqing; FJ Fujian; GD Guangdong; GS Gansu; GX Guangxi; GZ Guizhou; HA Henan; HB Hubei; HE Hebei; HI Hainan; HLJ Heilongjiang; HN Hunan; IM Inner Mongolia; JL Jilin; JS Jiangsu; JX Jiangxi; LN Liaoning; NX Ningxia; QH Qinghai; SC Sichuan; SD Shandong; SH Shanghai; SN Shaanxi; SX Shanxi; TJ Tianjin; XJ Xinjiang; XZ Xizang; YN Yunnan; ZJ Zhejiang.

While the proposed spatial matching framework captures fine-grained variability in solar irradiance, infrastructure density, and road layout, most existing studies apply aggregated regional assumptions that overlook these heterogeneities. Such simplifications can distort both the technical assessment of PV potential and the formulation of region-specific deployment strategies. To quantify these effects, we conduct an analysis comparing the proposed spatial matching approach with a traditional province-level averaging method across different system configurations, including fixed-tilt, single-axis tracking, and dual-axis tracking, as shown in Fig. 4; the methodological details are provided in the “Methods” section.

Nationally, spatial aggregation introduces a systematic underestimation of −0.9% to −1.1% across configurations. The deviation is −0.86% for the fixed-tilt configuration, −1.05% for single-axis tracking, and −1.11% for dual-axis tracking, which suggests that while the overall magnitude of bias remains moderate, notable spatial heterogeneity persists across regions. At the provincial scale, the northwestern, central, and southern regions exhibit relatively pronounced deviations. Provinces such as Qinghai, Sichuan, and Hainan show systematic underestimations of approximately 7–11%. In contrast, the northeastern provinces, together with several northern ones, including Inner Mongolia, Shandong, and Shanxi, display consistently small errors within ±2%, indicating that in areas with uniformly distributed road networks and broad climatic zones, spatial aggregation effects remain comparatively stable. For fixed-tilt systems, high installation densities in land-constrained regions help mitigate spatial mismatch, resulting in relatively low estimation errors. In contrast, single-axis and dual-axis tracking systems result in slightly higher estimation errors because of the compounded effects of dynamic tracking interactions with localized microclimatic variability, which increase the sensitivity to spatial aggregation assumptions. These results highlight the coupling between system configurations and estimation biases arising from capacity factor assumptions.

**Fig. 5:**
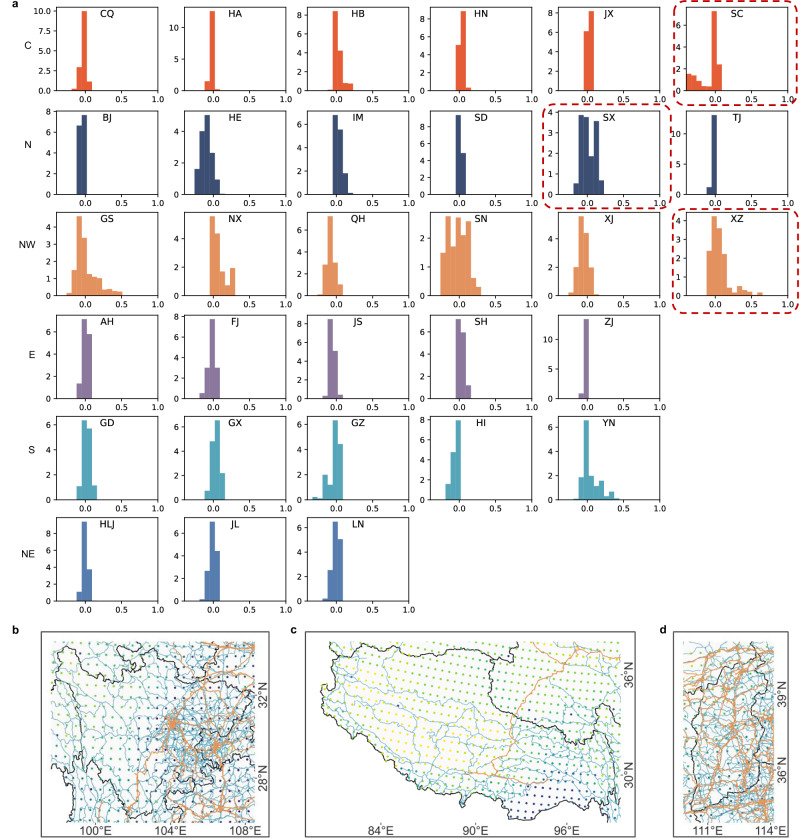
Intra-provincial spatial distribution of estimation errors and their driving mechanisms. **a** Frequency distribution of relative errors in annual roadside PV generation for fixed-tilt systems, comparing the conventional method based on provincial capital cities with the spatial matching approach. Red dashed boxes indicate the provinces selected for the case studies shown in (**b**–**d**). **b–d** Spatial distributions of solar irradiance and transportation networks in Sichuan (**b**), Xizang (**c**), and Shanxi (**d**). The dots represent selected spatial grid points, with the color intensity indicating the solar irradiance level. Orange lines denote railways, and blue lines denote highways. E east; NE northeast; N north; NW northwest; S south; C central. PV photovoltaic; AH Anhui; BJ Beijing; CQ Chongqing; FJ Fujian; GD Guangdong; GS Gansu; GX Guangxi; GZ Guizhou; HA Henan; HB Hubei; HE Hebei; HI Hainan; HLJ Heilongjiang; HN Hunan; IM Inner Mongolia; JL Jilin; JS Jiangsu; JX Jiangxi; LN Liaoning; NX Ningxia; QH Qinghai; SC Sichuan; SD Shandong; SH Shanghai; SN Shaanxi; SX Shanxi; TJ Tianjin; XJ Xinjiang; XZ Xizang; YN Yunnan; ZJ Zhejiang. Map outlines were generated from Natural Earth public-domain data, and transportation network data were derived from OpenStreetMap contributors, available under the Open Database License; maps were drawn and modified by the authors in QGIS v.3.32.0.

Beyond province-level aggregation and point-wise mapping, an alternative approach is a raster-based pipeline using satellite-derived surface radiation data. Raster-based methods typically offer finer nominal spatial resolution, allowing detailed capture of local gradients in solar radiation; however, they can introduce retrieval biases over complex terrain, adjacency effects due to heterogeneous land-cover types, and intermittent temporal gaps caused by cloud-masking processes. Furthermore, raster-vector overlay operations at hourly resolution increase computational complexity at the national scale. In contrast, our proposed KD-tree spatial matching method, employing NASA POWER hourly radiation data, ensures continuous temporal coverage without gaps, providing robust stability for annual assessments and long-term scenario analysis. Additionally, by reducing hourly spatial computations to a one-time nearest-neighbor assignment, our KD-tree approach substantially decreases computational runtime and memory demands while maintaining sufficient spatial accuracy for corridor-level infrastructure planning. Thus, our method effectively balances spatial accuracy, temporal continuity, and computational scalability for nationwide assessments. A detailed summary table comparing these approaches is available in Supplementary Information. To further investigate the regional drivers and spatial characteristics of aggregation bias, Fig. 5 provides a detailed analysis of provincial-level deviations in annual generation estimates for the fixed-tilt configuration. The distribution of segment-level relative errors across provinces is presented in Fig. 5a, while Fig. 5b–d illustrates the spatial interactions between solar irradiance gradients and road network layouts for three representative provinces, highlighting the mechanisms through which spatial heterogeneity leads to estimation bias under conventional capacity factor assumptions.

In southern and central provinces with unevenly distributed road networks, such as Yunnan, estimation errors are generally skewed towards overestimation and exhibit greater dispersion, reflecting considerable intra-provincial variability in segment-level deviations. Conversely, provinces with relatively balanced road networks, such as Hunan and Jiangxi, demonstrate less dispersed error distributions. Northern and northwestern provinces, such as Gansu, Shanxi, and Inner Mongolia, present more concentrated error distributions predominantly shifted towards underestimation, reflecting systematic shortfalls in conventional estimates associated with sparse road networks.

Aggregation bias is driven primarily by three interacting spatial factors. The combination of pronounced solar irradiance gradients and highly clustered transportation corridors plays a dominant role in southern regions. In Sichuan (Fig. 5b), for example, solar irradiance declines sharply from west to east, whereas roads are tightly concentrated within the central Sichuan Basin. Conventional methods based on provincial capital cities (located in areas of relatively lower solar resource) fail to represent the superior irradiance conditions along major transportation corridors, systematically underestimating the overall PV potential, as indicated by the extended left tail in Fig. 5a. Spatial misalignment between road network clusters and reference locations further contributes to marked biases in western provinces such as Xizang. In Xizang, the road network extends horizontally and is primarily concentrated in regions with lower solar irradiance, whereas the reference point (provincial capital) is situated in an area of higher irradiance. Consequently, conventional estimates exhibit systematic overestimation of approximately 5% at the provincial scale. Localized topographical complexity increases the fine-scale variability in solar resources and produces bidirectional estimation biases in certain northern regions. In Shanxi (Fig. 5d), while the road network is fairly evenly distributed, complex terrain and surface reflectivity generate sharp transitions between high- and moderate-irradiance zones at microspatial scales. Conventional methods, typically relying on fixed regional-average meteorological values, fail to capture these localized variations, resulting in simultaneous over- and underestimation across different road segments. Consequently, these segment-level errors aggregate into a distinctive bimodal distribution, highlighting the intrinsic limitation of coarse-scale estimation methods in resolving fine-scale spatial heterogeneity of solar irradiance along transportation corridors.

Together, these spatial mechanisms demonstrate that aggregation bias arises from the coupled effects of road network density, irradiance gradients, and spatial misalignment. The proposed spatial matching approach, by directly linking transportation segments with localized meteorological grids, effectively mitigates cumulative spatial mismatch effects, increases the accuracy of PV resource assessments in geospatially complex regions, and provides a robust basis for differentiated deployment strategies and integrated energy-transportation planning.

### Impact of roadside PV on power system supply-demand balance

To evaluate the impact of roadside PV deployment on the net load curves of power systems, this study explores the temporal characteristics of PV generation and their implications for the power system’s supply-demand balance. The net load, defined as the hourly regional electricity demand minus the hourly PV generation, directly affects the flexibility requirements of the grid, influencing the operational demands for resources such as energy storage and thermal power plants, as well as shaping the economic efficiency and integration capacity of PV systems. Specifically, two metrics are introduced to capture key aspects of the net load profile:

1. **Daily peak-to-valley differences (PTV)** quantify the range between the highest and lowest hourly net load within a day, reflecting the required daily ramping capacity and indicating the maximum intra-day operational stress on grid-balancing resources.
2. **Daily fluctuation mileage (MIL)** measures the cumulative absolute hourly ramps of net load over a day, capturing the overall variability and frequency of adjustments required from dispatchable resources, thus reflecting the intensity of operational flexibility demands.

**Fig. 6:**
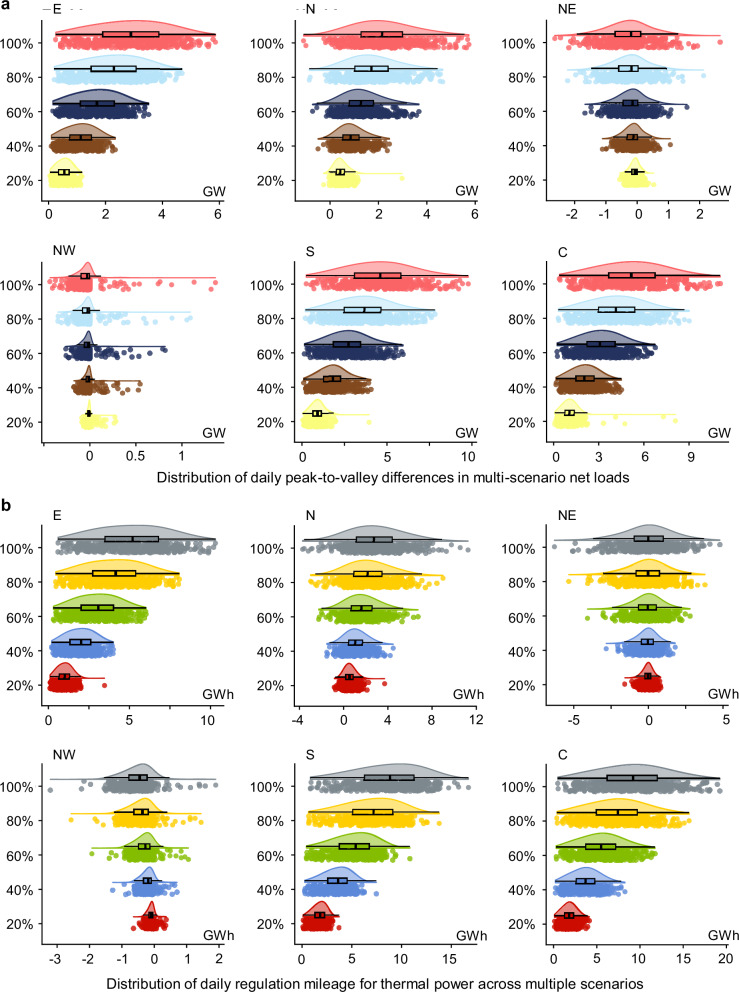
Impact of roadside PV deployment on the net load curve. **a** Changes in daily net load peak-to-valley differences across regions at different PV deployment levels compared with the baseline scenario (0% deployment). **b** Changes in daily thermal power adjustment mileage across regions at different PV deployment levels compared with the baseline scenario. In each subplot, the vertical axis indicates deployment levels of 20%, 40%, 60%, 80% and 100%, and the horizontal axis shows the change relative to the baseline scenario. Each raindrop plot shows 365 daily values, with individual dots representing daily data points and half-violins indicating the distribution density. Box plots are overlaid, with the center line indicating the median, the box bounds indicating the 25th and 75th percentiles, and the whiskers extending to the most extreme values within 1.5 times the interquartile range (IQR)^48^. Colors denote deployment levels. E east; N north; NE northeast; NW northwest; S south; C central. PV photovoltaic; AH Anhui; BJ Beijing; CQ Chongqing; FJ Fujian; GD Guangdong; GS Gansu; GX Guangxi; GZ Guizhou; HA Henan; HB Hubei; HE Hebei; HI Hainan; HLJ Heilongjiang; HN Hunan; IM Inner Mongolia; JL Jilin; JS Jiangsu; JX Jiangxi; LN Liaoning; NX Ningxia; QH Qinghai; SC Sichuan; SD Shandong; SH Shanghai; SN Shaanxi; SX Shanxi; TJ Tianjin; XJ Xinjiang; XZ Xizang; YN Yunnan; ZJ Zhejiang.

These two metrics are chosen because they comprehensively characterize the operational challenges imposed by variable renewable generation on power system operation, both the magnitude of short-term fluctuations (PTV) and the total flexibility burden (MIL). The regional load profiles enter the analysis explicitly through the hourly calculation of net load, combining spatially resolved PV output data with actual hourly demand patterns. **A detailed description of how these metrics are computed is provided in the Methods section**. Figure 6 illustrates the changes in these two metrics across different levels of roadside PV deployment and regional contexts, highlighting substantial variability in flexibility requirements driven by regional load and resource characteristics.

The integration of roadside PV systems reshapes regional power system load dynamics, with flexibility requirements strongly influenced by the match between generation profiles and local demand characteristics. As shown in Fig. 6, North China and Central China experience the most pronounced increases in daily net load variability as PV penetration increases. In the 100% deployment scenario, peak-to-valley differences in these regions increased by more than 15% compared with those in the low-penetration cases. This is due primarily to a mismatch between midday PV generation and industrial load curves that peak in the evening, resulting in deeper evening ramps and greater temporal volatility. The fluctuation mileage metric further confirms the heightened need for system flexibility in these areas, with the required ramping capacity increasing by 35–40% relative to the baseline levels.

In contrast, Northwest China and Northeast China demonstrate greater compatibility between PV output and electricity demand. The agricultural and residential load profiles in these regions align more closely with midday generation, resulting in more stable net load curves. In Northeast China, the prevalence of combined heat and power (CHP) systems during the heating season further buffers PV variability by absorbing excess generation, particularly in the winter months. As a result, even at high PV penetration, these regions show only modest increases in the peak-to-valley range and ramping mileage, which are generally below 15%.

These outcomes highlight three functional categories in terms of PV−flexibility interactions: (i) volatility-prone regions, such as N and C, which require substantial investment in storage, demand-side management, or interregional balancing; (ii) load-compatible regions, such as NW and NE, where PVs can be scaled with minimal system disruption; and (iii) hybrid areas that may require seasonal strategies. Understanding these patterns is critical for optimizing flexible resource deployment and realizing the full system value of roadside PV in a spatially differentiated manner.

### Carbon reduction potential of roadside PV

**Fig. 7:**
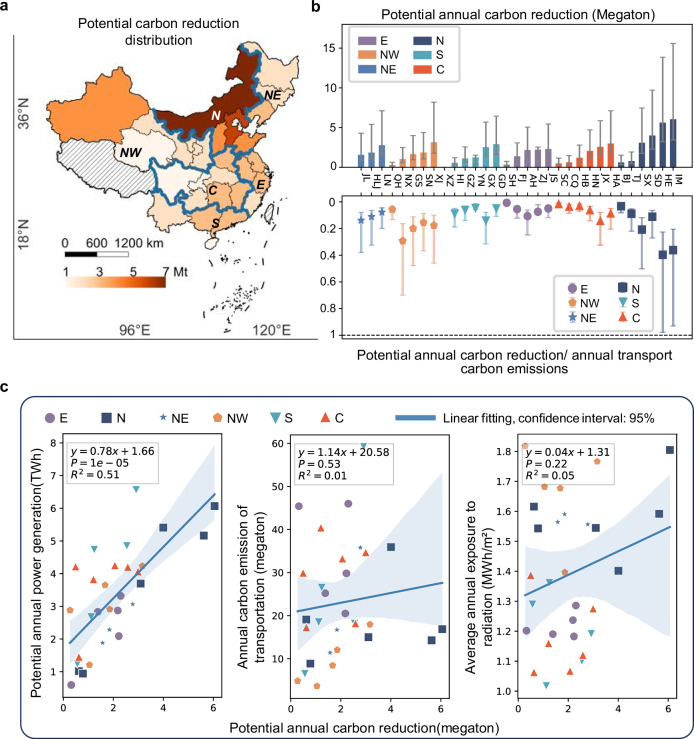
Carbon reduction potential of roadside distributed PV across regions. **a** Spatial distribution of the potential annual carbon reduction across the six regions of China under the baseline scenario (M2). The color scale indicates the potential annual carbon reduction, and blue lines denote regional boundaries. **b** Relationship between provincial roadside PV deployment and the equivalent ratio relative to transport-related CO_2_ emissions. The central values correspond to the baseline scenario (M2), and error bars extend from the minimum to maximum values across all 63 deployment scenarios. **c** Relationships between the potential annual carbon reduction and annual PV generation potential, annual transport carbon emissions, and annual solar irradiance across provinces. Symbols and colors denote the six regions. Blue lines indicate linear fits, shaded areas indicate 95% confidence intervals, and *P*-values and *R²*-values were obtained from simple linear regression. M2, tilted single-axis tracking with moderate deployment and 24% module efficiency; E east; NW northwest; NE northeast; N north; S, south; C central. PV photovoltaic; AH Anhui; BJ Beijing; CQ Chongqing; FJ Fujian; GD Guangdong; GS Gansu; GX Guangxi; GZ Guizhou; HA Henan; HB Hubei; HE Hebei; HI Hainan; HLJ Heilongjiang; HN Hunan; IM Inner Mongolia; JL Jilin; JS Jiangsu; JX Jiangxi; LN Liaoning; NX Ningxia; QH Qinghai; SC Sichuan; SD Shandong; SH Shanghai; SN Shaanxi; SX Shanxi; TJ Tianjin; XJ Xinjiang; XZ Xizang; YN Yunnan; ZJ Zhejiang. The map was drawn and modified by the authors in QGIS v.3.32.0, and map outlines were generated from Natural Earth public-domain data. The map shows Taiwan, Hong Kong and Macau for geographic context, whereas the quantitative analysis is restricted to mainland China.

This section evaluates the carbon reduction benefits of roadside PV deployment, in which fossil fuel-based electricity is replaced, and the transportation sector’s carbon emissions are reduced. The transportation-sector electricity demand referenced here specifically includes electricity consumption associated with electrified railway systems (both high-speed and conventional railways) and road transportation infrastructure (e.g., electric vehicle charging along highways). Maritime, aviation, and pipeline transportation are excluded from this analysis. Provincial transportation-related carbon emissions data used in this analysis are sourced from the China Emission Accounts and Datasets (CEADs), which provide direct CO₂ emissions from final fossil fuel consumption for the transportation, storage, and postal sectors, explicitly excluding indirect emissions from electricity or heat production^24^. Therefore, these emissions data represent direct final energy consumption, inherently excluding efficiency losses related to electricity generation, transmission, distribution, or vehicle charging processes. Using regional carbon emission data, the annual carbon reduction potential and its ratio with respect to transportation-related emissions are quantified to reveal regional disparities and trends, as shown in Fig. 7.

The carbon mitigation impact of roadside PV deployment in China’s transportation sector varies considerably across regions, reflecting differences in both the generation potential and baseline transportation emissions. Across all deployment scenarios, the national annual carbon reduction reaches 33.62−143.97 MtCO₂, equivalent to 4.89–20.95% of the latest available direct transport-related CO₂ emissions used here. North China and Central China account for nearly half of this total, led by provinces such as Hebei, Henan, and Shandong, where dense road networks coincide with high transport-related emissions. In contrast, Northwest China and Northeast China contribute less in absolute terms, despite their strong solar resource availability, due to limited underlying emissions. South China exhibits wide internal variability, with some provinces, such as Guangdong, achieving relatively high reductions and others, such as Yunnan and Guizhou, presenting low reductions because of a combination of weak infrastructure and modest generation capacity.

When normalized by transport-related CO_2_ emissions, the equivalent ratios provide a clearer view of regional decarbonization potential. North China and Northwest China show the highest equivalent ratios, 18.45% and 17.22%, respectively, indicating that roadside PV could deliver avoided emissions comparable in magnitude to a substantial share of current local transport-related CO_2_ emissions. This outcome is shaped not only by generation capacity but also by marginal carbon factors and emission baselines. In contrast, eastern and central provinces, despite high transportation activity, yield lower equivalent ratios (5–6%), suggesting a weaker relative mitigation effect under current resource-demand conditions. These patterns highlight the need to consider both absolute and relative metrics when evaluating mitigation potential.

Correlation analysis indicates a strong positive association between PV generation potential and carbon reduction, especially in resource-rich northern provinces. Baseline transportation emissions show moderate correlation, whereas solar irradiance, though having weaker correlation overall, is particularly influential in low-emission regions, such as Northwest China, where high insolation is associated with substantial carbon mitigation even at modest-scale PV deployment. These findings highlight the value of region-specific carbon accounting: in high-emission, infrastructure-dense provinces, PV can serve as a bulk offset mechanism, while in resource-rich but low-demand areas, it represents a targeted decarbonization option with high marginal value.

### Impact of PV tracking structures on roadside deployment potential

**Fig. 8:**
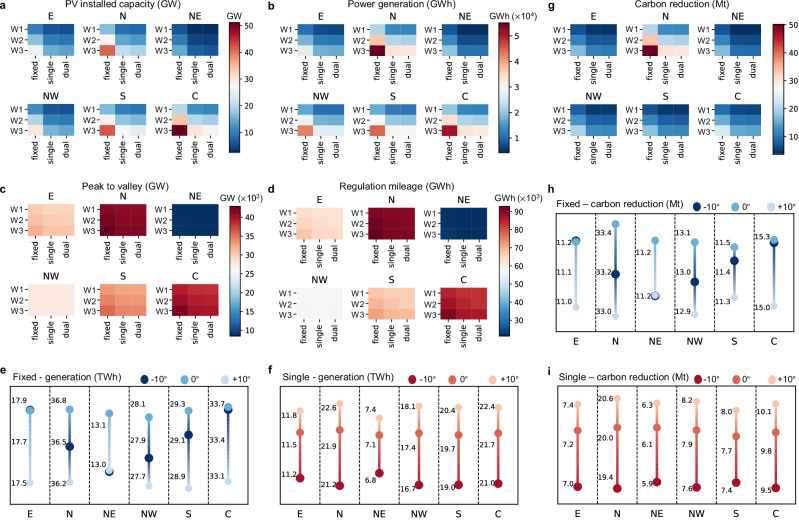
Sensitivity of tracker type and deployment rules (W1–W3) across macro-regions. Heatmaps (**a**–**d**, **g**) show the effects of tracker type (fixed-tilt, single-axis tracking and dual-axis tracking; x-axis) and deployment rule (W1–W3; y-axis) on installed capacity (**a**), annual power generation (**b**), annual sum of daily peak-to-valley net-load spans (**c**), annual sum of daily thermal regulation mileage (**d**), and annual carbon reduction (**g**) across the six macro-regions. Dot-range plots (**e**, **f**, **h**, **i**) summarize the corresponding regional outcomes for annual power generation (**e**, **f**) and annual carbon reduction (**h**, **i**) under different tracker configurations. Marker color intensity from dark to light denotes tilt-angle settings of optimal tilt −10°, optimal tilt, and optimal tilt +10°, respectively.W1, conservative deployment with 20% module efficiency; W2, moderate deployment with 24% module efficiency; W3, optimistic deployment with 28% module efficiency; E east; N north; NE northeast; NW northwest; S south; C central. PV photovoltaic; AH Anhui; BJ Beijing; CQ Chongqing; FJ Fujian; GD Guangdong; GS Gansu; GX Guangxi; GZ Guizhou; HA Henan; HB Hubei; HE Hebei; HI Hainan; HLJ Heilongjiang; HN Hunan; IM Inner Mongolia; JL Jilin; JS Jiangsu; JX Jiangxi; LN Liaoning; NX Ningxia; QH Qinghai; SC Sichuan; SD Shandong; SH Shanghai; SN Shaanxi; SX Shanxi; TJ Tianjin; XJ Xinjiang; XZ Xizang; YN Yunnan; ZJ Zhejiang.

To systematically understand how critical design parameters affect roadside PV deployment potential, we conducted a structured sensitivity analysis considering tracker type (fixed-tilt, tilted single-axis, dual-axis) and spatial deployment rules. Collectively, these factors determine roadside PV system’s installed capacities, electricity generation potential, carbon mitigation benefits, and grid flexibility performance across China’s transportation corridors. The results are presented in Fig. 8.

Our analyses indicate that fixed-tilt PV systems consistently offer the highest installed capacities and annual generation potentials nationwide due to their efficient land utilization and simplified structural requirements. As shown in Fig. 8a, b, fixed-tilt configurations achieve approximately 90% greater installable capacity and roughly 60% higher annual generation compared to dual-axis tracking systems. Although tracking methods, particularly tilted single-axis, can enhance annual electricity output through improved solar alignment, their larger land footprints, higher structural complexity, and increased installation costs often outweigh these yield gains in constrained corridor scenarios. In particular, tilted single-axis systems offer only modest incremental gains, about 8% in installed capacity and 3–5% in annual generation, relative to dual-axis systems, suggesting limited justification for their widespread deployment in densely packed transportation corridors. However, tilted single-axis tracking provides clear operational benefits in grid flexibility, particularly in regions with dense transportation networks and pronounced solar variability, such as Southern and Central China. Specifically, single-axis tracking markedly reduces annual peak-to-valley net load fluctuations and thermal power plant ramping mileage compared to fixed-tilt systems (Fig. 8c, d). These operational improvements enhance grid stability and integration capability, illustrating a critical advantage for tracking systems in managing intermittent renewable generation.

Regional performance disparities are shaped by latitude-dependent solar incidence angles and corridor spatial constraints. In high-latitude areas, such as Northeast China, fixed-tilt systems exhibit the greatest relative advantages due to steeper optimal tilt angles and lower winter sun elevations. Conversely, low-latitude provinces such as Guangdong and Yunnan, characterized by consistently high solar angles and limited roadside areas, exhibit minimal performance differences between fixed-tilt and tracking systems. Midlatitude regions, including North and Central China, present favorable conditions balancing solar availability and spatial flexibility, reinforcing fixed-tilt systems’ overall advantages in capacity deployment and electricity generation.

Further sensitivity analyses of tilt angle deviations reveal asymmetric impacts across regions. For fixed-tilt configurations, deviations of +10° from optimal angles result in greater generation losses compared to −10° deviations, particularly in mid- and low-latitude regions. This asymmetry arises from flatter tilt angles better aligning with dominant summer sun positions, suggesting that slight under-tilting could be advantageous. In contrast, high-latitude regions exhibit more symmetrical losses with both positive and negative tilt deviations due to steeper baseline angles and balanced seasonal solar paths. Evaluations of tilted single-axis systems, which currently derive their tilt angles from fixed-tilt optimal designs, indicate room for further optimization. All analyzed scenarios underperform relative to theoretical maxima, notably in high-irradiance regions such as Northwest China, where optimized tilt angles could enhance generation by more than 5%. These insights underscore the importance of region-specific geometric optimizations, especially given that even small percentage improvements can translate into substantial absolute benefits in large-scale deployments.

## Discussion

This study advances understanding of how spatial structure, infrastructure form, and solar resource distribution jointly shape the deployment potential and system effects of distributed solar generation in transportation systems. Three key structural mechanisms emerge. First, pronounced spatial decoupling exists between PV resource availability and transportation electricity demand, driven by the mismatch between solar irradiance gradients and freight activity concentrations. Second, estimation biases in PV potential are systematically linked to spatial aggregation effects, particularly in regions with concentrated or elongated road networks and steep irradiance gradients. Third, the choice of system configuration, such as the tracking method and tilt angle, interacts nonlinearly with local resource conditions, highlighting the need for deployment strategies that reflect both spatial heterogeneity and geometric sensitivity. In addition, sub-grid variability in irradiance can be more pronounced in complex-terrain and high-elevation regions due to terrain-induced gradients and heterogeneous cloud conditions. As a result, province-level estimates should be interpreted with appropriate caution in these settings, and project-level deployment would benefit from finer-scale irradiance products and site-specific shading assessments.

The region-specific deployment strategies proposed here closely align with China’s recent renewable-energy policies and infrastructure development plans. The Chinese government has explicitly encouraged the integration of photovoltaic systems with transportation infrastructure, emphasizing their coordinated deployment with electric vehicle charging facilities and local renewable energy utilization^25,26^. Additionally, recent policy developments facilitating direct renewable energy supply to targeted consumers have further strengthened support for roadside PV projects^27^. Such policy support provides clear guidance and implementation frameworks for roadside PV expansion. Based on regional resource conditions and energy demand profiles, North and Northwest China, with ample roadside space and superior solar irradiation, present opportunities for large-scale roadside PV deployment. In contrast, Eastern and Southern China, facing constrained land availability, should prioritize roadside PV coupled with storage and charging facilities to optimize local renewable energy utilization, while Central China might adopt a hybrid approach balancing generation capacity and grid flexibility needs. Insights from international experiences and existing studies also provide clear benchmarks and strategic guidance for roadside PV deployment in China. The European Union’s “Solar Highways” initiative, exemplified by the Rijkswaterstaat’s EU LIFE project, has successfully demonstrated integrated PV noise barriers along highways^28,29^. In the United States, the Federal Highway Administration’s Right-of-Way Alternative Use Framework enabled the Massachusetts Department of Transportation’s successful public-private partnerships for roadside PV installations^30–32^. Furthermore, highway operators like Switzerland’s ASTRA and Austria’s ASFINAG have successfully promoted market-driven PV projects along highways, accumulating valuable technical standards and viable business models^33–35^. These international policy practices and project experiences offer valuable benchmarks for China. Concurrently, China is rapidly enhancing its technical standards and policy frameworks for roadside PV deployment, including ongoing standardization efforts by the National Development and Reform Commission, ensuring critical technical support and policy guarantees for scaling up roadside PV.

Building on these insights and policy contexts, we clearly identify potential impacts and prioritize future research pathways. In the near term, densely populated, economically active regions in Eastern and Southern China with intensive energy demand but limited land availability should prioritize integrated roadside PV deployment combined with energy storage and charging infrastructure. Studies addressing dynamic factors, such as climate change impacts, electrification trends, and sector coupling (e.g., logistics, cold chains, urban mobility), should be prioritized in these regions to effectively meet growing local renewable energy needs and infrastructure demands. In the medium-to-long term, regions with abundant solar resources and available land, particularly Northwest and North China, are suitable for higher-standard, large-scale roadside PV deployments, including studies on integrating green-power direct connection schemes and cross-regional transmission networks. Additionally, exploring synergies between roadside PV and other linear infrastructures (such as transmission corridors, high-speed railways, and pipelines) in resource-rich regions represents an important long-term research direction. Clearly defining these priorities based on demand-driven and resource-driven contexts allows for more precise and effective deployment strategies, supporting China’s targeted, economically feasible energy transition.

However, the practical deployment of roadside PV systems also confronts multifaceted important constraints. Economic feasibility remains a critical consideration, encompassing critical dimensions including upfront investment costs, the cost-effectiveness of grid infrastructure upgrades, and long-term maintenance expenditures. Land-use conflicts, such as competition with agriculture, ecological conservation areas, or urban development, introduce additional layers of complexity requiring careful spatial planning and policy coordination. Socio-technical barriers, including legal rights-of-way restrictions, potential shading from vegetation, vehicles, or nearby structures, and road-safety buffer requirements, may further limit practical deployment. In addition, detailed system design, including panel orientation, tilt-angle setting, and shading-aware layout optimization, is important for maximizing performance and economic returns. Future site-specific studies should therefore incorporate these factors more explicitly in project-level feasibility assessment and planning. Although the analytical framework developed here is broadly applicable, its extension to other countries or infrastructure systems would require adaptation to local road-network characteristics, data availability, and regulatory conditions. More broadly, these findings show that spatially explicit, infrastructure-coupled energy modeling can provide a practical basis for integrating distributed photovoltaic deployment into transportation systems in a regionally differentiated manner. Rather than representing a uniform national resource, roadside photovoltaic deployment should be understood as a location-specific infrastructure strategy shaped by resource conditions, demand patterns, and implementation constraints. In this sense, the framework offers a scalable basis for embedding distributed renewables into large-scale built infrastructure and for supporting more coordinated, flexible, and carbon-efficient energy transitions.

## Methods

### Deployable area and installation capacity

To account for technical and terrain constraints, three deployment assumptions were used to define the usable width on one side of different transportation corridors^36^. Under the conservative assumption, the deployable widths were set to 3 m for railways, 1 m for highways, 0.5 m for national roads, and 0.25 m for provincial roads. Under the moderate assumption, the corresponding widths were 4 m, 1.5 m, 0.75 m, and 0.5 m, respectively. Under the optimistic assumption, the widths were 5 m for railways, 2 m for highways, 1 m for national roads, and 0.75 m for provincial roads. The deployable area was obtained by multiplying corridor length by the assumed deployable width. Under the three deployment assumptions, the national deployable area ranges from 1119.80 to 2037.49 square kilometers. Under the moderate assumption, the deployable areas are 808.44 square kilometers for railways and 770.21 square kilometers for highways.

The potential PV installation capacity, ${P}_{E}$, is calculated using Eq. (1):

1 $${P}_{E}=10\times \frac{{S}_{E}}{{S}_{B}}$$

where ${P}_{E}$ denotes the potential installation capacity. ${S}_{E}$ and ${S}_{B}$ denote the deployable area (m²), as derived in the previous calculation, and the land area required for a 10 MW PV system (m²), respectively. Equation (1) is derived from the Chinese Ministry of Land and Resources’ official industry standard *“Land quota of photovoltaic power station project” (TD/T 1075-2023)*^37^, which uses a 10 MW PV installation as a reference unit to standardize land-use calculations across different project scales. The 10-MW value is therefore used only as a benchmark for land-area conversion rather than as a limit on actual installed capacity. The reference land-area parameter inherently reflects practical engineering requirements, including module spacing, maintenance access, shading losses, and module availability. Accordingly, no additional correction coefficient was introduced in this study. This treatment provides a realistic and policy-consistent estimate of roadside photovoltaic deployment potential without requiring detailed modeling of specific engineering layouts.

Unless otherwise specified, the baseline calculation assumes monocrystalline silicon photovoltaic modules with a conversion efficiency of 24% and tilted single-axis tracking. The land-use parameters were further interpolated to account for latitude-dependent variation. At the national level, the potential installation capacity ranges from 40.91 to 202.84 GW across scenarios. Under the moderate assumption, the corresponding capacities are 39.17 GW for highways and 36.05 GW for railways.

### Matching road points with meteorological grid points

To account for spatial variability in resource conditions along transportation corridors, we developed a nearest-neighbor spatial matching method based on a KD-tree algorithm to associate each road point with its nearest meteorological grid point. Road infrastructure data from OpenStreetMap were first segmented into discrete spatial points at predefined intervals (for example, 1-km segments). These road points were then matched with 4133 meteorological grid points covering China from the NASA POWER dataset, which has a spatial resolution of approximately 0.5° (about 50 km)*.* Each segmented road point was assigned the hourly meteorological variables of its nearest grid point, including solar irradiance, temperature, and other relevant atmospheric conditions. Because the method does not involve a clustering step or explicit specification of cluster numbers, it remains computationally efficient while preserving sufficient spatial precision for national-scale assessment.

Let the meteorological grid-point set be denoted by *G* = ${\{{g}_{i}\}}_{i=1}^{n}$, where each grid point ${g}_{i}$ has latitude‒longitude coordinates (${{lat}}_{i}$, ${{lon}}_{i}$) and meteorological data ${w}_{i}$. Similarly, define the road point set *R* = ${\{{r}_{i}\}}_{j=1}^{m}$, where each road point ${r}_{j}$ has coordinates (${{lat}}_{j}$, ${{lon}}_{j}$). The distance ${d}_{a,b}$ between two points (${{lat}}_{a}$, ${{lon}}_{a}$) and (${{lat}}_{b}$, ${{lon}}_{b}$) is calculated using Eq. (2):

2 $$d=2{R}_{e}\times arcsin \left[\sqrt{{sin }^{2}\left(\frac{{la}{t}_{i}-{la}{t}_{j}}{2}\right)+cos ({la}{t}_{i})cos ({la}{t}_{j}){sin }^{2}\left(\frac{{lo}{n}_{i}-{lo}{n}_{j}}{2}\right)}\right]$$

where *R*_*e*_ is the Earth’s radius. A KD-tree is then constructed from the grid-point set G to enable efficient nearest neighbor searches^38^. For each road point ${r}_{j}\in R$, the matched meteorological grid point is determined using Eq. (3):

3 $$k\left(j\right)={arg } {min }_{i\in \left\{1,2,\ldots,n\right\}}d\left(\left({la}{t}_{j},{lo}{n}_{j}\right),\left({la}{t}_{i},{lo}{n}_{i}\right)\right)$$

where $k(j)$ represents the index of the grid point ${g}_{k(j)}$ nearest to road point ${r}_{j}$. The meteorological data assigned to road point ${r}_{j}$ are then given by ${w}_{k\left(j\right)}.$

Using the matched meteorological data, hourly photovoltaic generation is calculated for each road segment through the photovoltaic generation model described below. The resulting outputs are then aggregated to the provincial and regional levels to obtain hourly and monthly generation profiles across China.

### Converting meteorological data to PV power generation

Photovoltaic generation in this study was derived from meteorological variables, including solar irradiance, ambient temperature, and solar zenith angle, for 4133 grid points across China. The meteorological data have an hourly temporal resolution (8760 h per year) and a spatial resolution of 0.5° × 0.5°. Monocrystalline silicon photovoltaic modules with tilted single-axis tracking were assumed. The baseline tilt angle at each location was determined from more than 900 county-level latitude–longitude points, and the tilt angle of the nearest reference point was assigned to each roadside photovoltaic system.

Tilted surface irradiance was calculated from global horizontal irradiance, direct normal irradiance, and diffuse horizontal irradiance using the DIRINT-based decomposition framework. Direct normal irradiance was first estimated from global horizontal irradiance, and diffuse horizontal irradiance was then obtained using Eq. (4):

4 $${GH}{I}_{t}={DH}{I}_{t}+{DN}{I}_{t}\times cos {\theta }_{Z,t}$$

The tilted surface irradiance ${GT}{I}_{t}$ is then calculated using Eqs. (5–7):

5 $${GT}{I}_{t}=\max ({DN}{I}_{t}\times cos {\alpha }_{t},0)+{GH}{I}_{t}\times {f}_{{albedo},t}\times \frac{1-cos {\beta }_{t}}{2}+{DH}{I}_{t}\times {M}_{t}$$

6 $${M}_{t}=\left[\frac{1+cos {\beta }_{t}}{2}\right]\times \left[1+{F}_{t}\times {sin }^{3}\left(\frac{{\beta }_{t}}{2}\right)\right]\times \left[1+{F}_{t}\times {cos }^{2}{\alpha }_{t}\times {sin }^{3}{\theta }_{Z,t}\right]$$

7 $${F}_{t}=1-{\left(\frac{{DH}{I}_{t}}{{GH}{I}_{t}}\right)}^{2}$$

where ${GH}{I}_{t}$, ${DN}{I}_{t}$, and ${DH}{I}_{t}$ denote the global horizontal, direct normal, and diffuse horizontal irradiance at time *t*. Additional parameters include the solar zenith angle (${\theta }_{Z,t}$), panel tilt angle (${\beta }_{t}$), albedo factor (${f}_{{albedo},t}$), and incidence angle (${\alpha }_{t}$), shown as Eq. (8):

8 $${\alpha }_{t}=\arccos \left[cos {\beta }_{t}\times cos {\theta }_{z,t}+sin {\beta }_{t}\times sin {\theta }_{z,t}\times cos \left({\theta }_{A,t}-{\lambda }_{t}\right)\right]$$

where ${\theta }_{A,t}$ is the solar azimuth angle and ${\lambda }_{t}$ is the PV array azimuth angle, which aligns with the solar azimuth under single-axis tracking.

Photovoltaic power output was then calculated using Eq. (9)^39^:

9 $${P}_{{pvt}}={P}_{{PVR}}\left(\frac{{I}_{t}}{{I}_{0}}\right)\left[1+{\alpha }_{p}\left({T}_{t}-{T}_{{STC}}\right)\right]$$

where ${P}_{{pvt}}$ and ${P}_{{PVR}}$ denote the actual PV power output and rated power under standard test conditions (${I}_{0}$ = 1000 W/m^2^, ${T}_{{STC}}$ = 25 °C), respectively. ${I}_{t}$ denotes the actual irradiance received by the panel, and ${\alpha }_{p}$ is the power temperature coefficient (−0.0046). ${T}_{t}$ is the PV module temperature, which is calculated using Eq. (10):

10 $${T}_{t}={T}_{\alpha }+\frac{{I}_{t}}{1000}\left(\frac{{T}_{{NOCT}}-20}{0.8}\right)$$

where ${T}_{\alpha }$ represents the ambient temperature from the meteorological data. ${T}_{{NOCT}}$ is the nominal operating cell temperature

### Scenario design

We designed a comprehensive set of scenarios to capture realistic variations in the deployment potential of roadside photovoltaic systems across China. The scenario design considers three key dimensions that influence photovoltaic output and deployment scale: available roadside land resources, tracking configuration and tilt-angle setting, and module efficiency. These parameters directly affect installation capacity and electricity generation potential, while also being constrained by road conditions, terrain, engineering feasibility, economic factors, and construction limitations.

Available roadside land resources are represented by conservative, moderate, and optimistic assumptions based on the typical cross-sectional widths of transportation corridors, including railways and different classes of highways, such as expressways, national roads, and provincial roads^40,41^. Tracking configuration includes fixed-tilt, tilted single-axis, and dual-axis systems, reflecting commercially available photovoltaic technologies with different trade-offs between structural complexity, land-use intensity, and solar-radiation capture capability^42^. Tilt angle is treated as an additional design parameter because it affects the geometric relationship between photovoltaic modules and incoming solar radiation. For each location, the baseline setting adopts the locally optimal tilt angle, and deviations of ±10° are further considered to reflect realistic installation conditions^43^. Module efficiency is set at three levels, 20%, 24%, and 28%, representing conservative, moderate, and optimistic assumptions based on mainstream commercial monocrystalline silicon photovoltaic modules and national technical standards^44,45^.

Combining these dimensions yields a total of 27 installation scenarios and 63 generation scenarios, thereby covering a broad range of potential deployment outcomes. Detailed parameter settings, numerical values, and specific scenario definitions, including the representative minimum and maximum cases, are provided in Supplementary Information.

### Province level benchmark method

To benchmark the proposed spatial matching approach, we also consider a conventional province-level averaging method. In this benchmark, the provincial capital city of each province, obtained from the Gaode Map API (2023), is used as a fixed reference location. The meteorological data from the grid point closest to the provincial capital are then taken to represent the climatic conditions of the entire province. This representative value is subsequently used to estimate the roadside photovoltaic generation potential at the provincial level, shown as Eq. (11):

11 $${E}_{i}\left(t\right)=\,{H}_{k\left(i\right)}\left(t\right)\times {P}_{i}$$

where ${E}_{i}(t)$ denotes the estimated time series of roadside PV generation for province *i.* ${H}_{k\left(i\right)}(t)$ represents the annual capacity factor time series at the meteorological grid point closest to the provincial capital city of province *i*; and *P*_*i*_ denotes the total installable roadside PV capacity within province *i*. The index k(i) indicates the meteorological grid point that is spatially nearest to the provincial capital city.

### Net load metrics

Six PV deployment scenarios were defined (0%, 20%, 40%, 60%, 80%, and 100%), and hourly load and generation data were used to calculate the daily net load peak‒valley difference (PTV) and daily fluctuation mileage (MIL) for each scenario. To further analyse the variations across scenarios, the differences in these two metrics between the deployment scenarios (20%, 40%, 60%, 80%, and 100%) and the baseline scenario (0% deployment) were calculated. The formulas are defined using Eq. (12):

12 $$\left\{\begin{array}{c}{PT}{V}_{i,D}=max ({L}_{i,D,t})-min ({L}_{i,D,t})\hfill \\ {MI}{L}_{i,D}={\sum }_{t=1}^{23}|{L}_{i,D,t+1}-{L}_{i,D,t}|\hfill \\ i=0,1\ldots,5{;D}=1,2\ldots,365{;t}=1,2\ldots,24\end{array}\right.$$

where ${{PTV}}_{i,D}$ (in GW) and ${{MIL}}_{i,D}$ (in GWh) denote the daily net load peak-to-valley difference and daily fluctuation mileage, respectively. ${L}_{i,D,t}$ denotes the net load for scenario *i* at hour *t* on day *D*.

The differences in the two metrics between the deployment scenarios and the baseline are defined using Eq. (13):

13 $$\left\{\begin{array}{c}{PT}{V}_{i-0,D}={PT}{V}_{i,D}-{PT}{V}_{0,D}\,i=1,2\ldots,5\\ {MI}{L}_{i-0,D}={MI}{L}_{i,D}-{MI}{L}_{0,D}\,i=1,2\ldots,5\end{array}\right.$$

where ${{PTV}}_{i-0,D}$ and ${{MIL}}_{i-0,D}$ represent the differences in the daily net load peak-to-valley difference and thermal power adjustment mileage between scenario *i* and the baseline scenario (0% deployment) for day *D*.

### Uncertainty and practical constraints

Several potential sources of uncertainty and data biases should be noted in interpreting the results of this study. First, the meteorological dataset from NASA POWER has a spatial resolution of approximately 0.5° (about 50 km), which may not fully capture sub-grid variability in solar irradiance caused by local cloud dynamics, terrain effects, or other microclimatic conditions. In addition, mapping gridded meteorological fields to linear transportation corridors inevitably involves spatial representation and aggregation choices.

Second, local shading effects are not explicitly represented in the national-scale assessment. Potential obstructions from vegetation, roadside structures, buildings, safety barriers, and moving vehicles may reduce the actual photovoltaic output at specific project sites. Explicitly modeling these effects would require detailed site-level data and substantially increase computational complexity, which is beyond the scope of the present nationwide analysis. The generation estimates reported here should therefore be interpreted as technical potential under generalized exposure conditions. At the project scale, more detailed obstruction-aware approaches, such as LiDAR-based three-dimensional solar potential mapping^46^ or parametric design optimization^47^, would be valuable for quantifying shading-related losses more precisely.

Third, the transportation infrastructure data used in this study are derived from OpenStreetMap, which is widely used and openly accessible but may vary in completeness and positional accuracy across regions. Such variability may affect the estimation of deployable area and, consequently, installed photovoltaic capacity along transportation corridors. This source of uncertainty is especially relevant in regions where local infrastructure records are incomplete or where corridor classification is more heterogeneous.

Fourth, this study evaluates roadside photovoltaic deployment mainly from the perspective of technical potential and system-level impacts and does not explicitly incorporate grid-connection constraints. Factors such as proximity to substations, feeder hosting capacity, voltage-level requirements, and the cost of grid reinforcement may limit the practically deployable capacity in specific locations. The results should therefore be interpreted as theoretical or technical potential rather than directly bankable deployment potential. For practical implementation, project-level assessments should further examine local grid access conditions, including the spatial proximity of roads to substations and transmission lines, available feeder and substation headroom, and potential network upgrade costs.

### Reporting summary

Further information on research design is available in the Nature Portfolio Reporting Summary linked to this article.

## Supplementary information

Supplementary Information

Reporting Summary

Transparent Peer Review file

## Source data

Source Data

## Data Availability

The transportation network data used in this study were obtained from OpenStreetMap (https://www.openstreetmap.org/). Hourly meteorological data were obtained from the NASA Prediction Of Worldwide Energy Resources (POWER) project through the Data Access Viewer (https://power.larc.nasa.gov/data-access-viewer/). Provincial electricity consumption and carbon emission data for the transportation sector were obtained from the China Carbon Accounting Database (CEADs) (https://www.ceads.net/data/). Additional data supporting the findings of this study are available within the paper and its Supplementary Information. No restrictions apply to the public datasets used in this study beyond the terms of use of the original data providers. Source data are provided with this paper.

## Code availability

The custom code used in this study for roadside resource assessment, solar resource simulation, spatial matching between transportation segments and meteorological grids, hourly photovoltaic generation calculation, and error evaluation is available at GitHub: https://github.com/wuzyncepu/Revealing-infrastructure-coupled-solar-resources-for-transportation-decarbonization.

## References

[1] IPCC. Transport. In *Climate Change 2022 - Mitigation of Climate Change: Working Group III Contribution to the Sixth Assessment Report of the Intergovernmental Panel on Climate Change.* 1049-1160 (Cambridge Univ. Press, Cambridge, 2023).

[2] Speizer, S. et al. Integrated assessment modeling of a zero-emissions global transportation sector. *Nat. Commun.* **15**, 4439 (2024). 38789428 10.1038/s41467-024-48424-9 PMC11126718

[3] Li, T. T. et al. Integrating solar-powered electric vehicles into sustainable energy systems. *Nat. Rev. Electr. Eng.* **2**, 467–479 (2025).

[4] Federal Highway Administration. Historic step: all fifty states plus D.C. and Puerto Rico greenlit to move EV charging networks forward, covering 75,000 miles of highway. https://highways.dot.gov/newsroom/historic-step-all-fifty-states-plus-dc-and-puerto-rico-greenlit-move-ev-charging-networks (2022).

[5] Zhao, A. P. et al. Hydrogen as the nexus of future sustainable transport and energy systems. *Nat. Rev. Electr. Eng.* **2**, 447–466 (2025).

[6] Liu, X. et al. Transforming public transport depots into profitable energy hubs. *Nat. Energy* **9**, 1206–1219 (2024).

[7] PVresources. Photovoltaic noise barriers. https://www.pvresources.com/en/pvpowerplants/noisebarriers.php#notes (2022).

[8] Kakoulaki, G. et al. European transport infrastructure as a solar photovoltaic energy hub. *Renew. Sustain. Energy Rev.* **196**, 114344 (2024).

[9] Qiu, T. et al. Potential assessment of photovoltaic power generation in China. *Renew. Sustain. Energy Rev.* **154**, 111900 (2022).

[10] Ji, L. et al. Solar photovoltaics can help China fulfill a net-zero electricity system by 2050 even facing climate change risks. *Resour. Conserv. Recycl.* **186**, 106596 (2022).

[11] Ministry of Transport of the People’s Republic of China. Statistical bulletin on the development of the transportation industry in 2023. https://xxgk.mot.gov.cn/2020/jigou/zhghs/202406/t20240614_4142419.html (2024).

[12] Chen, Z. et al. Using existing infrastructures of high-speed railways for photovoltaic electricity generation. *Resour. Conserv. Recycl.* **178**, 106091 (2022).

[13] Nijsse, F. J. et al. The momentum of the solar energy transition. *Nat. Commun.* **14**, 6542 (2023). 37848437 10.1038/s41467-023-41971-7 PMC10582067

[14] Joshi, S. et al. High resolution global spatiotemporal assessment of rooftop solar photovoltaics potential for renewable electricity generation. *Nat. Commun.* **12**, 5738 (2021). 34611151 10.1038/s41467-021-25720-2 PMC8492708

[15] Bódis, K., Kougias, I., Jäger-Waldau, A., Taylor, N. & Szabó, S. A high-resolution geospatial assessment of the rooftop solar photovoltaic potential in the European Union. *Renew. Sustain. Energy Rev.* **114**, 109309 (2019).

[16] Zhang, Z. et al. Worldwide rooftop photovoltaic electricity generation may mitigate global warming. *Nat. Clim. Chang.* **15**, 393–402 (2025).

[17] European Commission Joint Research Centre. Photovoltaic Geographical Information System (PVGIS). https://joint-research-centre.ec.europa.eu/photovoltaic-geographical-information-system-pvgis_en (2017).

[18] Maclaurin, G. et al. *The Renewable Energy Potential (reV) Model: A Geospatial Platform for Technical Potential and Supply Curve Modeling.* (National Renewable Energy Laboratory, Golden, CO, 2019).

[19] Shi, M. et al. Unveiling deployable rooftop solar potential across Chinese cities. *Nat. Cities* **2**, 650–661 (2025).

[20] Zhang, Z. et al. Carbon mitigation potential afforded by rooftop photovoltaic in China. *Nat. Commun.* **14**, 2347 (2023). 37095101 10.1038/s41467-023-38079-3 PMC10126133

[21] Zhang, K. et al. Quantifying the photovoltaic potential of highways in China. *Appl. Energy* **324**, 119600 (2022).

[22] Mooney, P. & Minghini, M. A review of OpenStreetMap data. In *Mapping and the Citizen Sensor.* 37–59 (Ubiquity Press, London, 2017).

[23] Sparks, A. H. nasapower: a NASA POWER global meteorology, surface solar energy and climatology data client for R. *J. Open Source Softw.* **3**, 1035 (2018).

[24] Xu, J., Guan, Y., Oldfield, J., Guan, D. & Shan, Y. China carbon emission accounts 2020−2021. *Appl. Energy* **360**, 122837 (2024).

[25] State Council of the People’s Republic of China. Action plan for energy conservation and carbon reduction (2024–2025). https://english.www.gov.cn/policies/latestreleases/202405/29/content_WS66570ed0c6d0868f4e8e79d0.html (2024).

[26] Ministry of Transport of the People’s Republic of China et al. Guiding opinions on promoting the integration of transportation and energy. https://xxgk.mot.gov.cn/jigou/zhghs/202504/t20250425_4167770.html (2025).

[27] National Development and Reform Commission & National Energy Administration. Notice on promoting the orderly development of green-power direct connection. https://www.ndrc.gov.cn/xxgk/zcfb/tz/202505/t20250530_1398138.html (2025).

[28] Rijkswaterstaat. Solar highways: Innovative noise barrier. https://www.rijkswaterstaat.nl/en/projects/international-projects/solar-highways (2025).

[29] European Commission, LIFE Programme. LIFE13 ENV/NL/000971 Solar Highways. https://webgate.ec.europa.eu/life/publicWebsite/project/LIFE13-ENV-NL−000971/solar-panels-as-integrated-constructive-elements-in-highway-noise-barriers (2025).

[30] Federal Highway Administration. Right-of-Way use agreements; 23 CFR 710.405. https://www.ecfr.gov/current/title-23/part-710/section-710.405 (2025).

[31] Federal Highway Administration. Right-of-Way solar program, Massachusetts. https://www.fhwa.dot.gov/ipd/value_capture/strategies_in_practice/mass_solar_program.aspx (2025).

[32] Massachusetts Department of Transportation. Highway right-of-way solar project. https://www.mass.gov/info-details/massdot-renewable-energy-projects (2025).

[33] Swiss Federal Roads Office (FEDRO). Photovoltaic installations along national roads. https://www.astra.admin.ch/astra/fr/home/themes/energie-climat/photovoltaiques-routes-nationales.html (2025).

[34] Swiss Federal Roads Office (FEDRO). Roads and Traffic 2023/2024 - Developments, facts and figures. https://www.astra.admin.ch/dam/astra/en/dokumente/abteilung_direktionsgeschaefteallgemein/strassen-verkehr/strassen_und_verkehr_2023_2024_developments_facts_and_figures.pdf.download.pdf/roads_and_traffic_2023_2024_developments_facts_and_figures.pdf (2024).

[35] ASFINAG & IOB-Innovationsplattform. Producing electricity with the noise barrier. https://www.ioeb-innovationsplattform.at/fileadmin/user_upload/Media_Library/Uploads/Challenge/Documents/English_Translation_of_ASFINAG_IOEB-CHALLENGE.pdf (2020).

[36] Jia, L. M., Shi, R. F. & Ma, J. *Research on the Energy Potential of Land Transportation Infrastructure Assets in China.* (Science Press, Beijing, 2020).

[37] Ministry of Natural Resources of the People’s Republic of China. Land quota of photovoltaic power station project (TD/T 1075-2023). (Ministry of Natural Resources of the People’s Republic of China, Beijing, 2023).

[38] Cunningham, P. & Delany, S. J. K-nearest neighbour classifiers - a tutorial. *ACM Comput. Surv.* **54**, 128 (2021).

[39] Chen, S. et al. The potential of photovoltaics to power the Belt and Road Initiative. *Joule* **3**, 1895–1912 (2019).

[40] Ministry of Transport of the People’s Republic of China. Technical Standards for Highway Engineering (JTG B01-2014). (People’s Transportation Publishing House, Beijing, 2014).

[41] National Railway Administration of the People’s Republic of China. Code for Design of Railway Line (TB 10098-2017) (China Railway Publishing House, Beijing, 2017).

[42] Rodriguez-Gallegos, C. D. et al. Global techno-economic performance of bifacial and tracking photovoltaic systems. *Joule* **4**, 1514–1541 (2020).

[43] Nsengiyumva, W., Chen, S. G., Hu, L. & Chen, X. Recent advancements and challenges in solar tracking systems (STS): a review. *Renew. Sustain. Energy Rev.* **81**, 250–279 (2018).

[44] National Standard of the People’s Republic of China. Specification of photovoltaic power generation efficiency (GB/T 39857-2021) (State Administration for Market Regulation & Standardization Administration of China, Beijing, 2021).

[45] Green, M. A. Silicon solar cells step up. *Nat. Energy* **8**, 783–784 (2023).

[46] Waqas, H., Jiang, Y., Shang, J., Munir, I. & Khan, F. U. An integrated approach for 3D solar potential assessment at the city scale. *Remote Sens.* **15**, 5616 (2023).

[47] Waqas, H. et al. Enhancement of the energy performance of an existing building using a parametric approach. *J. Energy Eng.* **149**, 04022057 (2023).

[48] Allen, M. et al. Raincloud plots: a multi-platform tool for robust data visualization. *Wellcome Open Res.* **4**, 63 (2021). 31069261 10.12688/wellcomeopenres.15191.1 PMC6480976

