# Supplementary material for: National-level assessment of infrastructure-coupled roadside solar energy toward transportation decarbonization in China
Source: Nat Commun. 2026 Jun 2;17:7095. doi: 10.1038/s41467-026-73872-w (PMC13392230; doi:10.1038/s41467-026-73872-w)
Supplement: Supplementary file 1 — Supplementary Information [file 41467_2026_73872_MOESM1_ESM.pdf]

Supplementary Information for

National-level assessment of infrastructure-coupled roadside solar energy toward transportation decarbonization in China

Zhaoyuan Wu[1,†], Jianxiao Wang[2, †], Lanyi Wei[1], Bo Li[3], Lin Chen[4], Lu Zhang[5], Daniel M. Kammen[6], Gengyin Li[1], Ming Zhou[1,*] and Jie Song[2, 7*]

[1] State Key Laboratory of Alternate Electrical Power System with Renewable Energy Sources, North China Electric Power University, Beijing, China

[2] National Engineering Laboratory for Big Data Analysis and Applications, Peking University, Beijing, China

[3] School of Electrical Engineering, Guangxi University, Nanning, Guangxi Zhuang Autonomous Region, China

[4] Institute for Interdisciplinary Information Sciences, Tsinghua University, Beijing, China

[5] College of Information and Electrical Engineering, China Agricultural University, Beijing, China

[6] Energy and Resources Group, University of California Berkeley, Berkeley, CA, USA

[7] School of Advanced Manufacturing and Robotics, Peking University, Beijing, China

[†] These authors contributed equally: Zhaoyuan Wu, Jianxiao Wang

[*] Correspondence: zhouming@ncepu.edu.cn (M. Z.), jie.song@pku.edu.cn (J.S.)

Contents

Supplementary Figure 1. 1

Supplementary Figure 2. 1

Supplementary Figure 3. 2

Supplementary Figure 4. 3

Supplementary Table 1. 3

Supplementary Table 2. 4

Supplementary Table 3. 4

Supplementary Table 4. 5

Supplementary Table 5. 5

Supplementary Table 6. 6

Supplementary Table 7. 7

Supplementary Table 8. 8

Supplementary Table 9. 9

Supplementary Table 10. 11

Supplementary Table 11. 11

Supplementary Table 12. 12

Supplementary Table 13. 13

Supplementary Note 1. Data Sources and Processing 13

Supplementary Figure 5. 15

Supplementary Table 14. 16

Supplementary Note 2. Photovoltaic Potential Estimation Method and Key Parameter Descriptions 16

Supplementary Note 3. Scenario Settings and Parameter Matrix 18

Supplementary Note 4. Analysis of Key Influencing Factors 19

Supplementary Figure 6. 23

Supplementary Figure 7. 24

Supplementary Figure 8. 25

Supplementary Figure 9. 26

Supplementary Figure 10. 28

Supplementary Note 5. Investment cost estimation method for roadside PV projects 29

Supplementary Figure 11. 31

Supplementary References 31

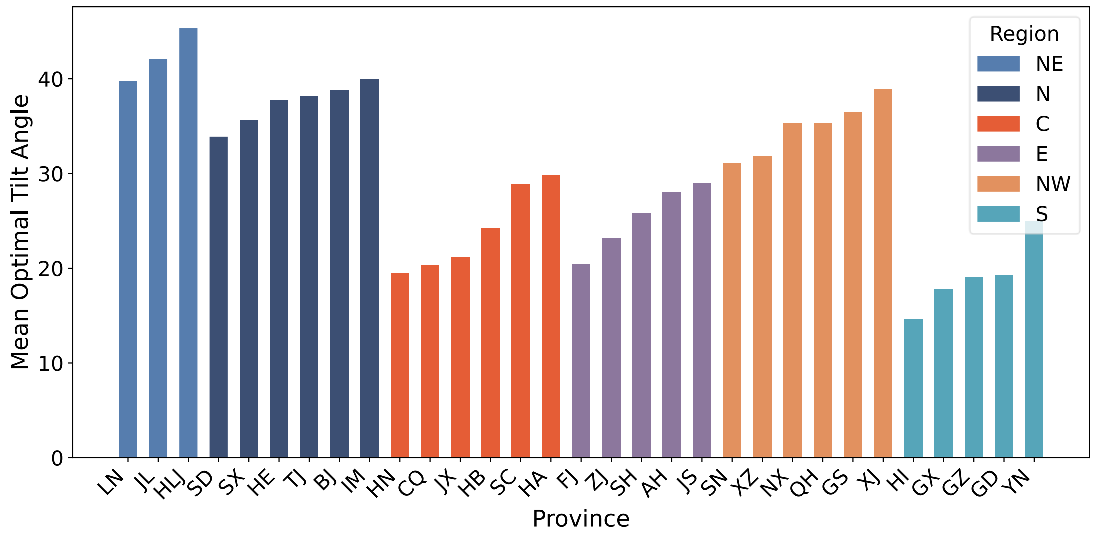

Supplementary Figure 1. Provincial mean optimal tilt angles. Bar chart showing the mean optimal tilt angle of photovoltaic (PV) installation for each province, in degrees (°). Different colours indicate the six regions. E, east; NE, northeast; N, north; NW, northwest; S, south; C, central. PV, photovoltaic; AH, Anhui; BJ, Beijing; CQ, Chongqing; FJ, Fujian; GD, Guangdong; GS, Gansu; GX, Guangxi; GZ, Guizhou; HA, Henan; HB, Hubei; HE, Hebei; HI, Hainan; HLJ, Heilongjiang; HN, Hunan; IM, Inner Mongolia; JL, Jilin; JS, Jiangsu; JX, Jiangxi; LN, Liaoning; NX, Ningxia; QH, Qinghai; SC, Sichuan; SD, Shandong; SH, Shanghai; SN, Shaanxi; SX, Shanxi; TJ, Tianjin; XJ, Xinjiang; XZ, Xizang; YN, Yunnan; ZJ, Zhejiang.

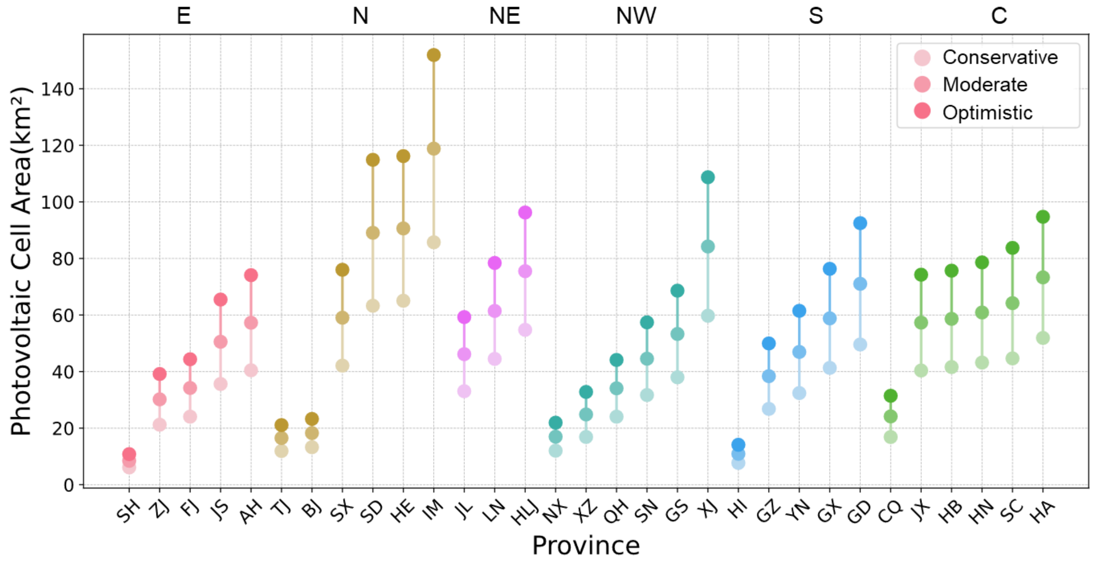

Supplementary Figure 2. Potential installable areas of highways and railways by province. Dots show the roadside PV installable area for each province (km²). Dot

colours from light to dark indicate the conservative, moderate, and optimistic deployment scenarios, respectively. E, east; NE, northeast; N, north; NW, northwest; S, south; C, central. PV, photovoltaic; AH, Anhui; BJ, Beijing; CQ, Chongqing; FJ, Fujian; GD, Guangdong; GS, Gansu; GX, Guangxi; GZ, Guizhou; HA, Henan; HB, Hubei; HE, Hebei; HI, Hainan; HLJ, Heilongjiang; HN, Hunan; IM, Inner Mongolia; JL, Jilin; JS, Jiangsu; JX, Jiangxi; LN, Liaoning; NX, Ningxia; QH, Qinghai; SC, Sichuan; SD, Shandong; SH, Shanghai; SN, Shaanxi; SX, Shanxi; TJ, Tianjin; XJ, Xinjiang; XZ, Xizang; YN, Yunnan; ZJ, Zhejiang.

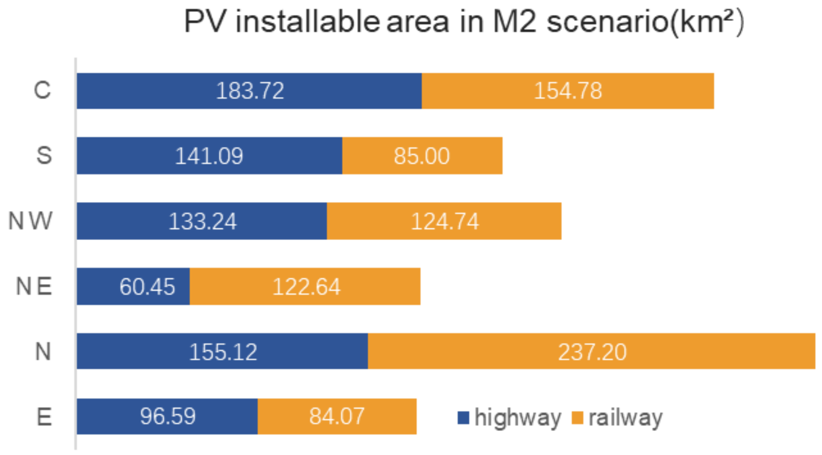

Supplementary Figure 3. Potential installable roadside PV areas by region in the M2 scenario. Blue bars show the roadside PV installable area for highways in each region under the M2 scenario (km^2), and orange bars show the roadside PV installable area for railways in each region under the M2 scenario (km^2). E, east; NE, northeast; N, north; NW, northwest; S, south; C, central. PV, photovoltaic.

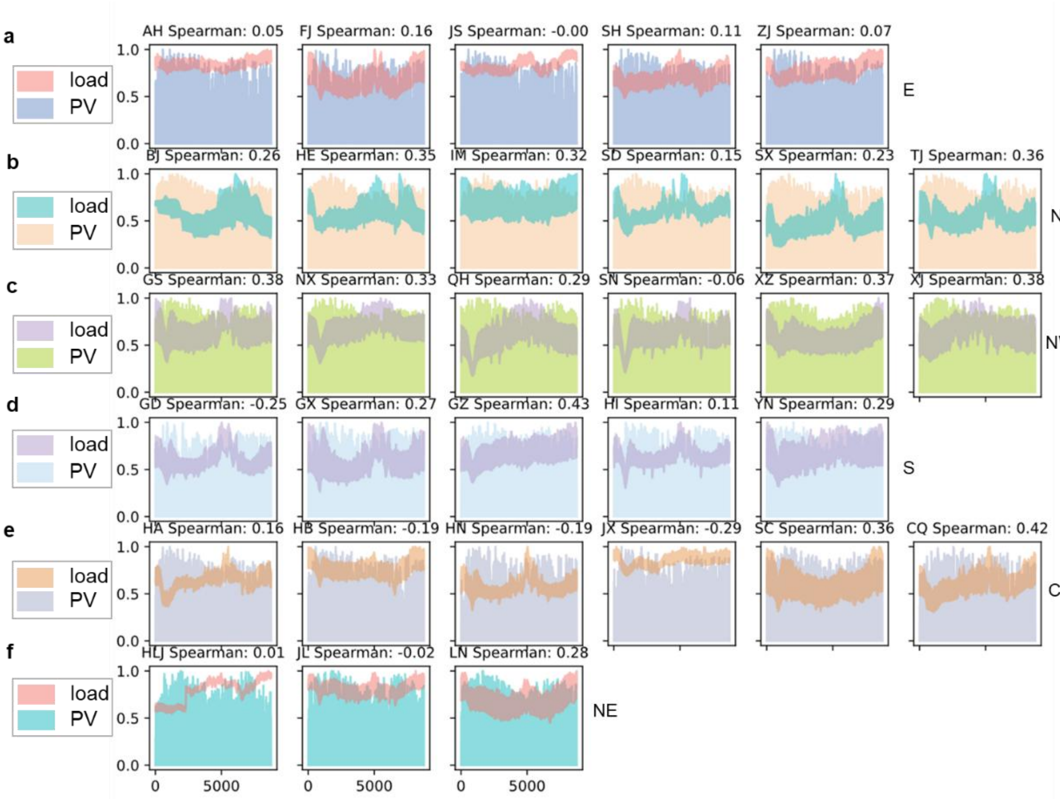

Supplementary Figure 4. Standardized 8,760-hour curves of roadside PV output and load by province. a-f, Standardized hourly curves of roadside PV generation and transportation electricity load for provinces in eastern China (**a**), northern China (**b**), northwestern China (**c**), southern China (**d**), central China (**e**) and northeastern China (**f**). In each provincial panel, the two coloured curves show the hourly annual load curve and the hourly roadside PV generation curve for the corresponding province. Both roadside PV generation and electricity load are normalized within each province to enable comparison of their temporal profiles. The Spearman coefficient shown in each panel is used as a descriptive rank-correlation metric to quantify the temporal alignment between hourly roadside PV generation and transportation electricity load. E, east; NE, northeast; N, north; NW, northwest; S, south; C, central. PV, photovoltaic; AH, Anhui; BJ, Beijing; CQ, Chongqing; FJ, Fujian; GD, Guangdong; GS, Gansu; GX, Guangxi; GZ, Guizhou; HA, Henan; HB, Hubei; HE, Hebei; HI, Hainan; HLJ, Heilongjiang; HN, Hunan; IM, Inner Mongolia; JL, Jilin; JS, Jiangsu; JX, Jiangxi; LN, Liaoning; NX, Ningxia; QH, Qinghai; SC, Sichuan; SD, Shandong; SH, Shanghai; SN, Shaanxi; SX, Shanxi; TJ, Tianjin; XJ, Xinjiang; XZ, Xizang; YN, Yunnan; ZJ, Zhejiang.

Supplementary Table 1. Description of highway data fields.

Field	Description
Osm_id	Unique identifier of the highway in the OpenStreetMap database
Code	Code representing the highway type
Fclass	Functional classification of the highway
Name	Name of the highway
Ref	Highway reference number
Oneway	One-way attribute of the highway
Maxspeed	Maximum speed limit of the highway
Layer	Layer or elevation level where the highway is located
Bridge	Indicates whether the segment is a bridge
Tunnel	Indicates whether the segment is a tunnel
Fclass_cn	Functional classification of the highway in Chinese
Type	Type of the highway
Coordinates	Latitude and longitude coordinates of the highway

Supplementary Table 2. Description of railway data fields.

Field	Description
Osm_id	Unique identifier of the railway in the OpenStreetMap database
Code	Code representing the railway type
Fclass	Functional classification of the railway
Name	Name of the railway
Layer	Layer or elevation level where the railway is located
Bridge	Indicates whether the segment is a bridge
Tunnel	Indicates whether the segment is a tunnel
Coordinates	Latitude and longitude coordinates of the railway

Supplementary Table 3. Description of meteorological data fields.

Field	Description
T2M	Temperature at 2 meters above ground
QV2M	Specific humidity at 2 meters above ground
RH2M	Relative humidity at 2 meters above ground
PS	Atmospheric pressure

WS10M	Wind speed at 10 meters above ground
WD10M	Wind direction at 10 meters above ground
ALLSKY_SFC_SW_DWN	All-sky surface shortwave downward irradiance
CLRSKY_SFC_SW_DWN	Clear-sky surface shortwave downward irradiance
CLRSKY_SFC_PAR_TOT	Total clear-sky surface photosynthetically active radiation
ALLSKY_KT	All-sky clearness index
ALLSKY_SRF_ALB	All-sky surface albedo
ALLSKY_SFC_UVA	All-sky surface UVA irradiance
ALLSKY_SFC_UVB	All-sky surface UVB irradiance
ALLSKY_SFC_UV_INDEX	All-sky surface ultraviolet index
SZA	Integrated solar zenith angle

Supplementary Table 4. Regional Division Scheme.

Region	Provinces
Northeast (NE)	Heilongjiang, Jilin, Liaoning
North China (N)	Beijing, Tianjin, Hebei, Inner Mongolia, Shanxi, Shandong
Northwest (NW)	Shaanxi, Gansu, Qinghai, Ningxia, Xinjiang, Xizang
East China (E)	Anhui, Jiangsu, Shanghai, Zhejiang, Fujian
Central China (C)	Henan, Sichuan, Chongqing, Hubei, Jiangxi, Hunan
South China (S)	Guangdong, Guangxi, Yunnan, Guizhou, Hainan

Supplementary Table 5. Latitude and longitude of provincial administrative centers.

Province	Longitude (°)	Latitude (°)
Anhui	117.13	31.93
Beijing	116.72	39.90
Fujian	119.30	26.07
Gansu	103.85	36.06
Guangdong	113.26	23.13
Guangxi	108.37	22.82
Guizhou	106.58	26.58
Hainan	110.20	20.04

Hebei	114.51	38.04
Henan	113.63	34.75
Heilongjiang	126.54	45.80
Hubei	114.27	30.60
Hunan	112.94	28.23
Jilin	125.32	43.82
Jiangsu	118.80	32.06
Jiangxi	115.86	28.68
Liaoning	123.46	41.68
Inner Mongolia	111.75	40.84
Ningxia	106.23	38.49
Qinghai	101.78	36.62
Shandong	117.12	36.65
Shanxi	112.55	37.87
Shaanxi	108.94	34.34
Shanghai	121.47	31.23
Sichuan	104.07	30.57
Tianjin	117.20	39.09
Xizang	91.17	29.65
Xinjiang	87.62	43.83
Yunnan	102.83	24.88
Zhejiang	120.21	30.25
Chongqing	106.55	29.56

Supplementary Table 6. Annual electricity consumption and carbon emissions in the transportation sector by province.

Name	Label	Region	Electricity Consumption in Transportation Sector (TWh)	Actual Carbon Emissions from Transportation Sector (Mt)
Anhui	AH	East (E)	6.18	20.37
Beijing	BJ	North (N)	6.15	18.98
Chongqing	CQ	Central (C)	3.58	17.16

Fujian	FJ	East (E)	4.83	25.08
Guangdong	GD	South (S)	16.31	59.19
Gansu	GS	Northwest (NW)	7.14	8.35
Guangxi	GX	South (S)	5.10	18.04
Guizhou	GZ	South (S)	4.42	18.36
Henan	HA	Central (C)	9.42	34.58
Hubei	HB	Central (C)	7.12	40.29
Hebei	HE	North (N)	13.06	14.16
Hainan	HI	South (S)	0.82	6.40
Heilongjiang	HLJ	Northeast (NE)	3.27	16.59
Hunan	HN	Central (C)	6.93	33.12
Inner Mongolia	IM	North (N)	5.52	16.74
Jilin	JL	Northeast (NE)	2.98	11.33
Jiangsu	JS	East (E)	11.83	45.87
Jiangxi	JX	Central (C)	5.08	18.09
Liaoning	LN	Northeast (NE)	6.75	35.67
Ningxia	NX	Northwest (NW)	1.32	3.56
Qinghai	QH	Northwest (NW)	1.16	4.77
Sichuan	SC	Central (C)	8.47	29.80
Shandong	SD	North (N)	15.43	35.79
Shanghai	SH	East (E)	5.81	45.28
Shaanxi	SN	Northwest (NW)	9.27	11.96
Shanxi	SX	North (N)	8.70	14.88
Tianjin	TJ	North (N)	4.80	8.79
Xinjiang	XJ	Northwest (NW)	4.91	17.83
Xizang	XZ	Northwest (NW)	NA	NA
Yunnan	YN	South (S)	4.42	1.24
Zhejiang	ZJ	East (E)	9.95	2.22

Supplementary Table 7. Parameter settings for roadside photovoltaic installation scenarios.

Scenario ID	Installation Mode	Type	Usable Width per Side (m)

1	Conservative Installation	Railway		3
		Highway	Expressway	1
			National Highway	0.5
			Provincial Highway	0.25
2	Moderate Installation	Railway		4
		Highway	Expressway	1.5
			National Highway	0.75
			Provincial Highway	0.5
3	Optimistic Installation	Railway		5
		Highway	Expressway	2
			National Highway	1
			Provincial Highway	0.75

Supplementary Table 8. Parameter settings for roadside photovoltaic installable capacity scenarios.

Scenario ID	Tracking Mode	Installation Mode	Power Generation Efficiency (%)
1	Fixed	Conservative Installation	20
2			24
3			28
4		Moderate Installation	20
5			24
6			28
7		Optimistic Installation	20
8			24
9			28
10	Tilted Single-Axis Tracking	Conservative Installation	20
11			24
12			28
13		Moderate Installation	20
14			24
15			28
16		Optimistic Installation	20

17			24
18			28
19	Dual-Axis Tracking	Conservative Installation	20
20			24
21			28
22		Moderate Installation	20
23			24
24			28
25		Optimistic Installation	20
26			24
27			28

Supplementary Table 9. Parameter settings for roadside photovoltaic power generation scenarios.

Scenario ID	Tracking Mode	Installation Mode	Power Generation Efficiency (%)	Tilt Angle Setting (°)
1	Fixed	Conservative Installation	20	optimal-10
2				optimal
3				optimal+10
4			24	optimal-10
5				optimal
6				optimal+10
7			28	optimal-10
8				optimal
9				optimal+10
10		Moderate Installation	20	optimal-10
11				optimal
12				optimal+10
13			24	optimal-10
14				optimal
15				optimal+10
16			28	optimal-10
17				optimal
18				optimal+10
19		Optimistic Installation	20	optimal-10
20				optimal
21				optimal+10
22			24	optimal-10

23				optimal
24				optimal+10
25			28	optimal-10
26				optimal
27				optimal+10
28	Tilted Single-Axis Tracking	Conservative Installation	20	optimal-10
29				optimal
30				optimal+10
31			24	optimal-10
32				optimal
33				optimal+10
34			28	optimal-10
35				optimal
36				optimal+10
37		Moderate Installation	20	optimal-10
38				optimal
39				optimal+10
40			24	optimal-10
41				optimal
42				optimal+10
43			28	optimal-10
44				optimal
45				optimal+10
46		Optimistic Installation	20	optimal-10
47				optimal
48				optimal+10
49			24	optimal-10
50				optimal
51				optimal+10
52			28	optimal-10
53				optimal
54				optimal+10
55	Dual-Axis Tracking	Conservative Installation	20	\
56			24	\
57			28	\
58		Moderate Installation	20	\
59			24	\
60			28	\
61		Optimistic Installation	20	\
62			24	\
63			28	\

Supplementary Table 10. Regional comparison of roadside photovoltaic potential installed capacity. W1, conservative deployment with 20% module efficiency; W2, moderate deployment with 24% module efficiency; W3, optimistic deployment with 28% module efficiency.

Tracking Methods	Region	PV Installed Capacity (GW)		
		W1	W2	W3
Fixed-tilt	E	9.94	16.84	25.39
	N	16.32	27.34	40.95
	NE	5.80	9.63	14.35
	NW	11.40	19.39	29.28
	S	16.03	27.56	41.86
	C	19.78	33.73	51.02
	total	79.27	134.49	202.84
Single-axis tracking	E	5.74	9.73	14.66
	N	8.30	13.91	20.83
	NE	2.64	4.38	6.53
	NW	5.98	10.18	15.38
	S	10.03	17.23	26.17
	C	11.61	19.79	29.93
	total	44.29	75.22	113.50
Dual-axis tracking	E	5.35	9.06	13.67
	N	7.66	12.82	19.21
	NE	2.46	4.04	6.03
	NW	5.52	9.38	14.18
	S	9.23	15.87	24.11
	C	10.70	18.25	27.60
	total	40.91	69.43	104.79

Supplementary Table 11. Regional comparison of roadside photovoltaic generation potential in main scenarios. W1, conservative deployment with 20% module efficiency; W2, moderate deployment with 24% module efficiency; W3, optimistic deployment with 28% module efficiency.

Tracking Methods	Region	PV Generation (TWh)		
		W1	W2	W3
Fixed-tilt	E	10.54	17.85	26.90
	N	21.94	36.74	55.01

	NE	7.91	13.13	19.57
	NW	16.47	28.03	42.34
	S	17.02	29.26	44.45
	C	19.74	33.68	50.95
	Total	93.62	158.70	239.23
Single-axis tracking	E	6.86	11.63	17.53
	N	13.26	22.20	33.25
	NE	4.33	7.19	10.71
	NW	10.37	17.66	26.68
	S	11.61	19.96	30.33
	C	12.85	21.92	33.16
	Total	59.28	100.56	151.66
Dual-axis tracking	E	6.58	11.16	16.82
	N	12.89	21.56	32.29
	NE	4.25	6.99	10.41
	NW	10.15	17.28	26.12
	S	11.24	19.33	29.36
	C	12.36	21.08	31.90
	Total	57.47	97.39	146.90

Supplementary Table 12. Regional comparison of roadside photovoltaic carbon mitigation potential in main scenarios. W1, conservative deployment with 20% module efficiency; W2, moderate deployment with 24% module efficiency; W3, optimistic deployment with 28% module efficiency.

**Tracking Methods**	**Region**	**Carbon Mitigation Potential (Mt)**		
		**W1**	**W2**	**W3**
Fixed-tilt	E	6.62	11.20	16.87
	N	19.98	33.45	50.07
	NE	6.79	11.28	16.81
	NW	7.74	13.08	19.68
	S	6.70	11.49	17.43
	C	8.98	15.29	23.11
	total	56.80	95.78	143.97
Single-axis tracking	E	4.30	7.27	10.96
	N	12.05	20.17	30.19
	NE	3.73	6.19	9.22
	NW	4.73	8.00	12.04

	S	4.56	7.82	11.86
	C	5.84	9.95	15.03
	total	35.20	59.39	89.31
Dual-axis tracking	E	4.12	6.97	10.50
	N	11.72	19.59	29.34
	NE	3.66	6.02	8.97
	NW	4.62	7.80	11.75
	S	4.40	7.56	11.46
	C	5.62	9.58	14.48
	total	34.15	57.52	86.50

Supplementary Table 13. The number of grids corresponding to different resolutions.

grid resolution	number of grids	grid resolution	number of grids
0.5° ×0.5°	4133	9° ×9°	24
1° ×1°	1071	10° ×10°	22
2° ×2°	301	11° ×11°	18
3° ×3°	144	12° ×12°	18
4° ×4°	91	13° ×13°	15
5° ×5°	63	14° ×14°	13
6° ×6°	47	15° ×15°	14
7° ×7°	36	16° ×16°	12
8° ×8°	31	17° ×17°	9

Supplementary Note 1. Data Sources and Processing

The network data were obtained from the official website of the open-source mapping platform OpenStreetMap (OSM), including shapefile datasets of China’s railway and highway networks in 2022. The network data are divided into highway data and railway data. Preliminary cleaning was performed on both datasets using QGIS, during which bridge and tunnel segments were excluded. Further processing steps were as follows: for the railway system, data such as metro lines, light rail, and cable cars were removed, retaining only the primary usable railway data. For the highway system, bicycle lanes, connecting roads, and similar data were excluded, retaining three categories of usable highways: expressways, national highways (excluding expressways), and

provincial highways.

Based on the cleaned usable data, the highway and railway networks covered in this study encompass all provinces and municipalities of mainland China. The railway dataset contains over 300,000 segments, while the highway dataset contains over 640,000 segments. According to name-based statistics, the dataset covers more than 3,231 railways (with some unnamed railway segments) and more than 13,783 highways (with some unnamed highway segments).

The meteorological data were sourced from the NASA POWER project platform, which provides global datasets on solar energy, meteorology, and climate. The dataset includes files corresponding to 4,133 grid points uniformly distributed across mainland China in 2022. The meteorological data files include variables such as irradiance, temperature, humidity, and solar position. The spatial resolution is 0.5°×0.5°, and the temporal resolution is 1 hour. The number of grid points per province ranges from 19 (Hainan) to 568 (Inner Mongolia), and the number of coordinate points per municipality ranges from 5 (Tianjin) to 28 (Chongqing). The specific meteorological indicators used in this study are listed in the following table.

To facilitate regional analysis and summarization, the country was divided into six major regions based on the existing grid partitioning method. The latitude and longitude coordinates of the administrative centers of each province were obtained from the Gaode Map API. The annual electricity consumption and carbon emissions data for the transportation, warehousing, and postal services sectors in each province were sourced from the latest 2021 dataset provided by the China Carbon Accounting Database (CEADs) [1].

The carbon reduction potential for each province was estimated by multiplying the provincial roadside PV generation potential by the corresponding average provincial carbon emission factor, which influences the spatial distribution of roadside PV carbon reduction potential (Supplementary Figure 5). Xizang was excluded from the carbon reduction potential analysis because of the absence of provincial carbon emission factor data, and was therefore included only in analyses of roadside PV generation potential. The national carbon emission factor generally exhibits a decreasing trend from north to south, indicating that roadside photovoltaics hold substantial carbon reduction value in northern and northeastern regions where thermal power generation accounts for a large proportion of the electricity mix.

We provide a clear summary table comparing major open-source radiation datasets

(Supplementary Table 14) explicitly highlighting their spatial resolution, temporal continuity, data availability, and key meteorological variables.

As illustrated in the table, we provide a comparative summary of selected open-source solar radiation datasets. Among these, NASA POWER offers global coverage and continuous hourly temporal resolution, making it particularly suitable for national or continental-scale planning and scenario assessments. ERA5 has finer spatial resolution and a richer set of meteorological variables, making it suitable for detailed climate–energy coupling analyses, though with greater computational overhead and data-processing requirements. The NSRDB provides solar radiation data at half-hourly or hourly intervals primarily for the United States (CONUS), making it ideal for site-specific photovoltaic studies and model validation, though global coverage is partial. Himawari-8 satellite-derived radiation datasets provide fine spatial (~2–5 km) and temporal (10-minute intervals) resolutions within the Asia-Pacific region, offering clear advantages for detailed local-scale analyses, such as city-level or corridor-level studies.. However, Himawari-8 datasets require more complex data processing, including cloud screening, interpolation, and gap-filling, leading to higher computational demands. Considering the national-scale objective of our study, we selected NASA POWER as the primary data source in this work.

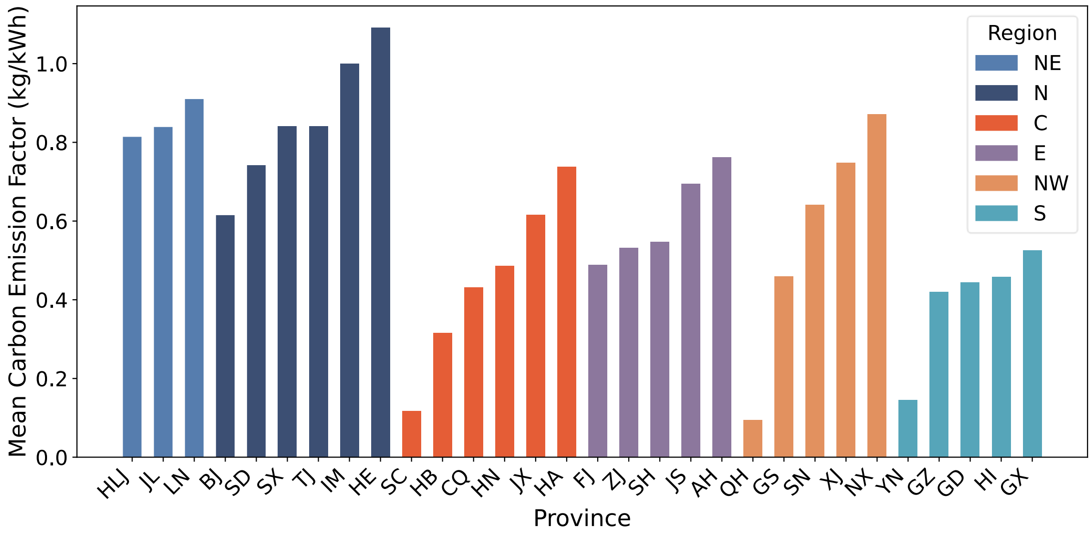

Supplementary Figure 5. Provincial average carbon emission factors. Bar chart showing the mean carbon emission factor for each province (kg/kWh). Different colours indicate the six regions. E, east; NE, northeast; N, north; NW, northwest; S, south; C, central. PV, photovoltaic; AH, Anhui; BJ, Beijing; CQ, Chongqing; FJ, Fujian; GD,

Guangdong; GS, Gansu; GX, Guangxi; GZ, Guizhou; HA, Henan; HB, Hubei; HE, Hebei; HI, Hainan; HLJ, Heilongjiang; HN, Hunan; IM, Inner Mongolia; JL, Jilin; JS, Jiangsu; JX, Jiangxi; LN, Liaoning; NX, Ningxia; QH, Qinghai; SC, Sichuan; SD, Shandong; SH, Shanghai; SN, Shaanxi; SX, Shanxi; TJ, Tianjin; XJ, Xinjiang; XZ, Xizang; YN, Yunnan; ZJ, Zhejiang.

Supplementary Table 14. Comparison of selected open-source radiation datasets. GHI, global horizontal irradiance; DNI, direct normal irradiance; DHI, diffuse horizontal irradiance; CONUS, contiguous United States.

Data Source	Spatial Resolution	Temporal Continuity	Data Availability	Key Meteorological Variables
NASA POWER (used here)	0.5°×0.5° (~55 km)	Hourly, globally continuous (no gaps)	Open-source, global	Solar irradiance (GHI, DNI, DHI), temperature, wind, humidity
ERA5 (ECMWF Reanalysis) [2]	~0.25° × 0.25° (~28 km)	Hourly, globally continuous (no gaps)	Open-source, global	Solar irradiance (GHI), temperature, wind, pressure, precipitation
NSRDB (NREL, USA) [3]	~4 km (CONUS), ~10 km (Global)	Half-hourly or hourly, mostly continuous (gap-filled)	Open-source, primarily US/global partial coverage	Solar irradiance (GHI, DNI, DHI), cloud cover, meteorology
Himawari-8 Satellite [4]	~2–5 km (Asia-Pacific region)	10-min intervals, intermittent data gaps (cloud masking)	Partially open (processed products), Asia-Pacific only	Cloud cover, surface irradiance (GHI, DNI)
Surface Observations (ground stations)	Point measurements (station-level)	Variable (typically hourly), gaps possible	Limited & variable	Direct irradiance measurements, detailed weather variables

Supplementary Note 2. Photovoltaic Potential Estimation Method and Key Parameter Descriptions

Installed Capacity Estimation Assumptions

The potential installed capacity of roadside PV systems was estimated using supplementary equation (1):

$$P_E = 10 \times \frac{S_E}{S_B} \tag{1}$$

where P_E denotes the potential installation capacity. S_E and S_B denote the

Deployable area (m²), as derived in the previous calculation and the Land area required for a 10 MW PV system (m²), respectively. The specific parameter settings for S_B are based on the "*Land quota of photovoltaic power station project*" (TD/T 1075-2023).

In this study, monocrystalline silicon photovoltaic panels, which are more commonly used, were assumed by default. Therefore, in the baseline scenario (M2), photovoltaic panels with a power generation efficiency of 24% and a tilted single-axis tracking system were selected. The corresponding land use index for the photovoltaic array was applied, and interpolation based on latitude was performed to better capture the impact of highway latitude on land use.

Tilted Surface Irradiance Calculation

The tilted surface irradiance (GTI) was derived from global horizontal irradiance (GHI), direct normal irradiance (DNI), and diffuse horizontal irradiance (DHI) using the DIRINT model [5]. DNI was first estimated from GHI, and DHI was then calculated using supplementary equation (2):

$$GHI_t = DHI_t + DNI_t \times \cos\theta_{Z,t} \tag{2}$$

Tilted surface irradiance was then calculated using supplementary equation (3)-(5) [6,7]:

$$GTI_t = \max\left(DNI_t \times \cos\alpha_t, 0\right) + GHI_t \times f_{albedo,t} \times \frac{1-\cos\beta_t}{2} + DHI_t \times M_t \tag{3}$$

$$M_t = \left[\frac{1+\cos\beta_t}{2}\right] \times \left[1 + F_t \times \sin^3\left(\frac{\beta_t}{2}\right)\right] \times \left[1 + F_t \times \cos^2\alpha_t \times \sin^3\theta_{Z,t}\right] \tag{4}$$

$$F_t = 1 - \left(\frac{DHI_t}{GHI_t}\right)^2 \tag{5}$$

where GHI_t, DNI_t, and DHI_t denote the global horizontal, direct normal, and diffuse horizontal irradiance at time t, respectively. Additional parameters include the solar zenith angle ($\theta_{Z,t}$) panel tilt angle (β_t), albedo factor ($f_{albedo,t}$), and incidence angle (α_t), shown as supplementary equation (6):

$$\alpha_t = \arccos\left[\cos\beta_t \times \cos\theta_{z,t} + \sin\beta_t \times \sin\theta_{z,t} \times \cos\left(\theta_{A,t} - \lambda_t\right)\right] \tag{6}$$

where $\theta_{A,t}$ is the solar azimuth angle, and λ_t is the PV array azimuth angle, which aligns with the solar azimuth under single-axis tracking.

Tilt Angle Settings

For the optimal tilt angles used in this study, we first collected existing optimal tilt angle data for fixed photovoltaic systems across cities and counties in China, covering more than 900 regions [8]. Subsequently, each grid point was matched to the geographically nearest region with known optimal tilt angle values based on latitude and

longitude.

Supplementary Note 3. Scenario Settings and Parameter Matrix

Roadside Photovoltaic Installation Scenario Settings

Based on the cross-sectional characteristics of Chinese highways, this study defined three installation scenarios for the photovoltaic system on both sides of different highways: a conservative installation scenario, a moderate installation scenario, and an optimistic installation scenario [9]. In each scenario, the available installation width on one side of both highways and railways was specified. For highways, the available width was categorized into three types: expressways, national highways, and provincial highways.

Roadside Photovoltaic Installed Capacity Scenario Settings

When evaluating the potential installed capacity of roadside photovoltaic systems, two key factors were considered: the available roadside area and the photovoltaic land use index. The available roadside area is affected by the installation width assumptions described above, while the photovoltaic land use index depends on the tracking system and the power conversion efficiency of the photovoltaic panels. Accordingly, we set 27 installation scenarios based on three variables: photovoltaic tracking system, installation width, and power conversion efficiency.

- Photovoltaic tracking system: Three types were considered: fixed, tilted single-axis tracking, and dual-axis tracking systems. The land requirement per unit capacity increases in this order.
- Installation width: As previously described, this was divided into conservative, moderate, and optimistic scenarios.
- Power conversion efficiency: This refers to the ability of photovoltaic modules to convert solar energy into electricity. Lower efficiency requires more land to achieve the same installed capacity. According to the *Technical Specifications for Photovoltaic Power Generation Efficiency*, the initial efficiency of monocrystalline silicon photovoltaic modules should not be lower than 17.8%. Based on laboratory data and real-world project performance, we set three efficiency levels: 20%, 24%, and 28%.

Roadside Photovoltaic Power Generation Potential Scenario Settings

When assessing the power generation potential of roadside photovoltaic systems, we further considered the influence of tilt angles on the capacity factor, expanding the scenarios established for installed capacity. The matched optimal tilt angle data at each grid point range from 12° to 54°. Therefore, for both fixed and tilted single-axis tracking systems, we established three tilt angle scenarios: optimal tilt angle ±10°. In total, 63 power generation scenarios were calculated in this study.

Supplementary Note 4. Analysis of Key Influencing Factors

Relationship Between Photovoltaic Installation Proportion and Transportation Self-Sufficiency in Different Regions

To further analyze the matching degree between transportation electricity demand and local photovoltaic resources across different regions, we estimated the required photovoltaic installation proportions under the condition of achieving complete self-sufficiency in transportation electricity consumption, based on the maximum photovoltaic generation scenario.

Specifically, we used the annual transportation electricity consumption of each province as the demand-side baseline and compared it with the roadside photovoltaic potential in each region to calculate the theoretical photovoltaic installation proportion required to achieve full transportation electricity self-sufficiency. The results are shown in Supplementary Figure 6.

The eastern and northern regions generally face the greatest difficulty in achieving transportation electricity self-sufficiency. This is mainly due to the highly developed transportation systems in these regions, which result in large electricity demands, while the highway resources available for photovoltaic installation are relatively limited. Additionally, several eastern coastal provinces are constrained by lower effective solar irradiance, further exacerbating the challenge of achieving transportation electricity self-sufficiency.

In contrast, the difficulty is lower in the northeastern and central regions, which have more abundant highway space and greater photovoltaic installation potential. Moreover, several western and southern provinces, such as Qinghai, Xinjiang, Hainan, and Yunnan, can theoretically achieve transportation electricity self-sufficiency with minimal photovoltaic installation proportions, providing a strong foundation for regional energy export and the construction of deeply integrated transportation–

photovoltaic systems.

Comparison Between the Averaging Method and the Proposed Matching Method

In addition to the provincial capital-point scenario discussed in the main text, we also evaluated the error and distribution of the averaging method. The averaging method refers to calculating the mean power generation sequence of all grid points within a province and using this mean sequence to represent the entire province's 8,760-hour roadside photovoltaic power generation profile.

The error percentages and distributions generated by the averaging method, in comparison to those produced by the matching method proposed in this study, are shown in Supplementary Figure 7 and Supplementary Figure 8.

For the averaging method, the use of the mean generation sequence across all grid points within a province reduces geographically and meteorologically induced regional errors. The resulting error distribution is more symmetric, and the mean error is smaller.

Sensitivity analysis of provincial PV generation errors with varying meteorological grid resolutions

We conducted a systematic sensitivity analysis to examine how varying the meteorological grid resolution from the baseline of 0.5°×0.5° to coarser grids (ranging from 1°×1° up to 17°×17°) influences our provincial-level PV generation estimates (Supplementary Figure 9).

We assume that finer-resolution meteorological grids provide more detailed spatial representation of local climatic conditions and therefore serve as a useful reference for PV potential estimates. Thus, we use the 0.5°×0.5° setting as the baseline reference for this sensitivity analysis. As illustrated in Supplementary Figure 9, estimation errors systematically increase as the meteorological grid resolution becomes coarser, indicating that PV assessments are sensitive to spatial input resolution. Notably, while the maximum national-level relative error remains relatively modest (up to 5.7%), provincial-level errors are substantially greater, reaching as high as 76.6%. Regionally, Southern China (S) generally exhibits lower and more stable relative errors, whereas Central China (C) demonstrates higher and more volatile errors across varying grid resolutions. Small provinces (e.g., Beijing, Shanghai, Tianjin, and Hainan) consistently show elevated and fluctuating errors due to their limited geographic extent, which intensifies sensitivity to minor shifts in meteorological grid points at coarser resolutions. Such

provinces inherently have fewer representative grid points at lower spatial resolutions, making their estimates particularly susceptible to spatial misalignment and localized meteorological variability.

Additionally, we identified pronounced extreme errors in provinces characterized not only by small area but also by uneven road-network distributions. For example, Chongqing, Ningxia, and particularly Sichuan, exhibit pronounced errors under coarser resolutions. Sichuan, notably located at the boundary of distinct solar resource zones and characterized by an extremely uneven distribution of transportation infrastructure, shows pronounced sensitivity starting from grid resolutions as coarse as 10°×10° degrees. Conversely, provinces with larger land areas and relatively homogeneous distributions of both transportation networks and solar resources (e.g., Inner Mongolia, Zhejiang, Heilongjiang) exhibit greater robustness, maintaining consistently low estimation errors (approximately 2%) even at moderately coarse resolutions (up to approximately 9°×9° degrees). Interestingly, despite extensive land areas, provinces in Northwest China demonstrate sensitivity similar to smaller provinces, primarily due to uneven transport infrastructure distribution and large latitudinal spans, causing substantial variability in local solar radiation.

These results indicate that the suitability of spatial meteorological resolution is closely tied to provincial land area, spatial homogeneity of infrastructure, and local variability in solar resource distribution. Our chosen baseline of 0.5°×0.5° resolution emerges as a well-balanced compromise, maintaining computational efficiency while effectively minimizing spatial aggregation biases. For future practical deployments and detailed local-scale assessments, adopting finer meteorological, such as satellite-derived datasets, or integrating detailed local analyses, such as LiDAR-based solar resource mapping, would help capture localized meteorological variability and infrastructure characteristics. Such practices are particularly useful in smaller provinces or in regions with heterogeneous infrastructure distribution, where they can help reduce estimation errors and support more reliable infrastructure planning and PV project implementation.

Sensitivity of provincial PV generation estimates to meteorological inputs and resolution coarsening

We note that the NASA POWER meteorological product used in the main analysis is publicly available at a maximum spatial resolution of 0.5°. An important

methodological question is therefore whether using this native 0.5° product could materially affect the estimated provincial annual roadside PV generation potentials, either due to grid resolution or due to dataset-specific construction.

To examine the sensitivity of provincial estimates to using the native 0.5° NASA POWER product, we compared provincial annual PV generation potentials computed using native NASA POWER (0.5°×0.5°) with those computed using native ERA5 (0.25°×0.25°) under an identical downstream pipeline (same variable extraction, PV conversion procedure, and solar-position calculation via pvlib). Supplementary Figure 10 provides a nationwide sensitivity check on whether a native 0.5° product is sufficiently representative. Using native ERA5 (0.25°) as a reference under an identical downstream pipeline, the comparison between native NASA POWER (0.5°) and native ERA5 (0.25°) shows percent-level differences with clear regional structure. As shown in panels (a) and (c), deviations are predominantly negative for many provinces (NASA POWER yielding lower provincial annual PV generation potentials than ERA5), with the national aggregate offset on the order of a few percent (panel c, dashed line). Regionally, larger negative offsets are concentrated in complex-terrain/high-elevation regions (e.g., Northwest and parts of Central China), reaching approximately ~5% at the macro-region level, whereas Northeast China exhibits a positive offset (panel c). At the provincial level, deviations can approach ~10% for some provinces (panel a). These patterns suggest that a native 0.5° product may miss sub-grid variability in certain settings, particularly where spatial gradients and terrain complexity are stronger; accordingly, province-level estimates should be interpreted with appropriate caution.

For reference only, Supplementary Figure 10 also includes a within-ERA5 resolution comparison between native ERA5 (0.25°) and an ERA5 representation at 0.5° obtained via consistent re-gridding/subsampling (panels (b) and (d)), which contextualizes the marginal impact of grid resolution within a fixed dataset. As expected, this resolution-only comparison yields much smaller deviations at the provincial annual aggregation scale (maximum absolute difference 0.248% and ~60% of provinces below 0.1% in absolute deviation). Consistent with the reviewer's comment, this within-ERA5 resolution comparison is not used to validate the physical adequacy of the native NASA POWER product; rather, it provides a magnitude reference under a fixed product.

Taken together, Supplementary Figure 10 supports two clarifications for interpreting the main analysis. First, the within-ERA5 resolution comparison (0.25° versus an ERA5 0.5° representation obtained via consistent re-gridding/subsampling) provides a

resolution-sensitivity context only and does not establish native-dataset adequacy. Second, the native NASA POWER–ERA5 comparison shows that differences associated with native meteorological inputs at 0.5° versus 0.25° can be material at the provincial annual scale, especially in complex-terrain regions; we therefore disclose this sensitivity and discuss its implications in the revised manuscript.

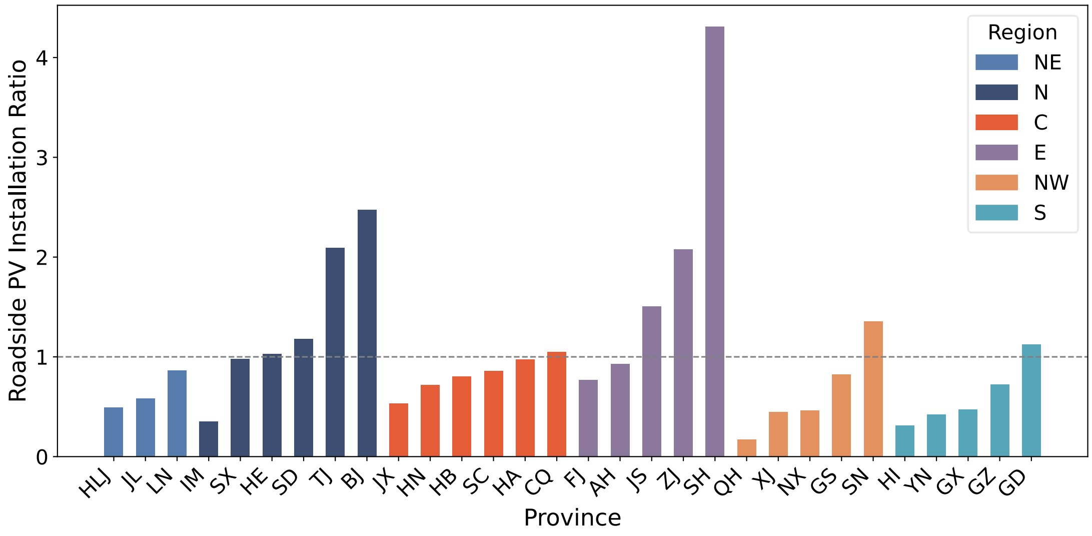

Supplementary Figure 6. Required roadside PV installation proportions for transportation electricity self-sufficiency by province. Bar chart showing the roadside photovoltaic installation ratio required for each province to achieve transportation electricity self-sufficiency under the deployment scenario with the maximum roadside PV generation. The horizontal dashed line at y = 1 marks the threshold installation ratio required to satisfy the self-sufficiency target, corresponding to full deployment of the available roadside PV resources. Different colours indicate the six regions. E, east; NE, northeast; N, north; NW, northwest; S, south; C, central. PV, photovoltaic; AH, Anhui; BJ, Beijing; CQ, Chongqing; FJ, Fujian; GD, Guangdong; GS, Gansu; GX, Guangxi; GZ, Guizhou; HA, Henan; HB, Hubei; HE, Hebei; HI, Hainan; HLJ, Heilongjiang; HN, Hunan; IM, Inner Mongolia; JL, Jilin; JS, Jiangsu; JX, Jiangxi; LN, Liaoning; NX, Ningxia; QH, Qinghai; SC, Sichuan; SD, Shandong; SH, Shanghai; SN, Shaanxi; SX, Shanxi; TJ, Tianjin; XJ, Xinjiang; XZ, Xizang; YN, Yunnan; ZJ, Zhejiang.

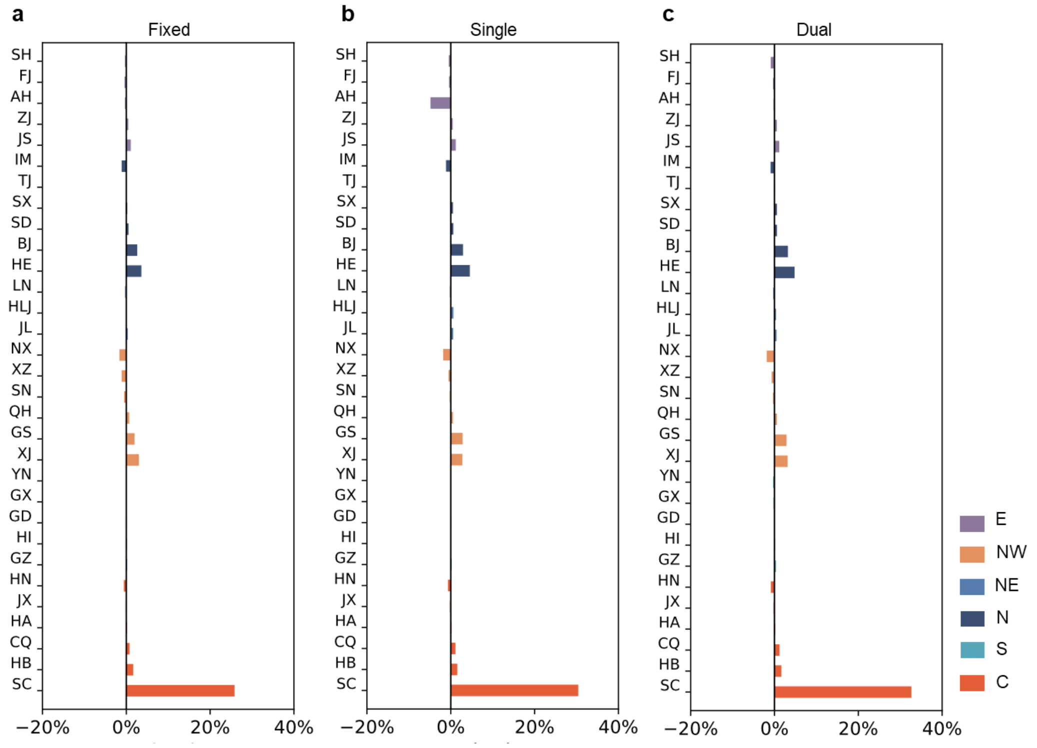

Supplementary Figure 7. Error proportions of roadside PV output from the averaging method compared to the matching method. a-c, Relative errors in provincial roadside photovoltaic power generation estimated using the averaging method relative to the matching method under fixed-tilt (**a**), single-axis tracking (**b**) and dual-axis tracking (**c**) configurations. Each horizontal bar represents the relative error for one province, calculated as (estimated − reference)/reference, where the reference scenario adopts the matching method and the estimated scenario adopts the averaging method. Positive and negative values indicate overestimation and underestimation, respectively. Colours denote the six regions. E, east; NE, northeast; N, north; NW, northwest; S, south; C, central. PV, photovoltaic; AH, Anhui; BJ, Beijing; CQ, Chongqing; FJ, Fujian; GD, Guangdong; GS, Gansu; GX, Guangxi; GZ, Guizhou; HA, Henan; HB, Hubei; HE, Hebei; HI, Hainan; HLJ, Heilongjiang; HN, Hunan; IM, Inner Mongolia; JL, Jilin; JS, Jiangsu; JX, Jiangxi; LN, Liaoning; NX, Ningxia; QH, Qinghai; SC, Sichuan; SD, Shandong; SH, Shanghai; SN, Shaanxi; SX, Shanxi; TJ, Tianjin; XJ, Xinjiang; XZ, Xizang; YN, Yunnan; ZJ, Zhejiang.

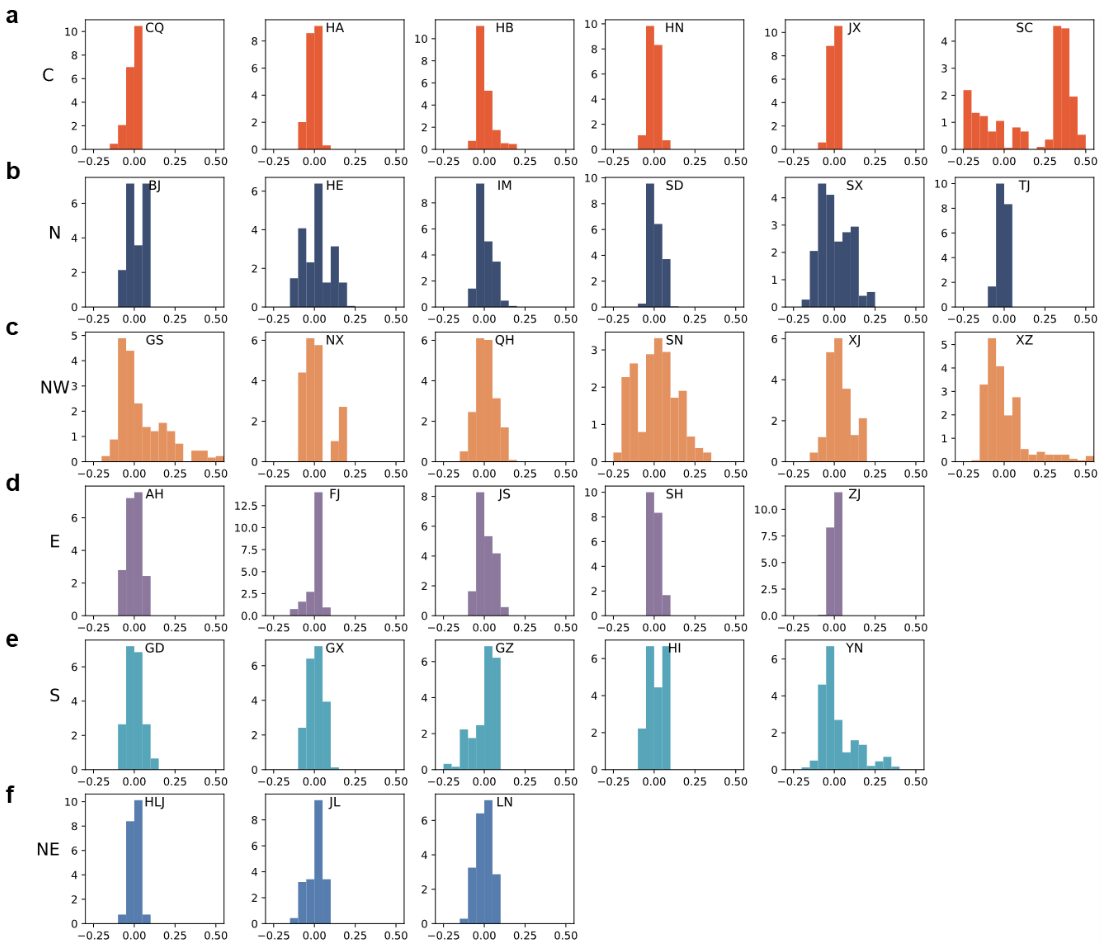

Supplementary Figure 8. Frequency distribution histogram of roadside photovoltaic output errors using the averaging method. a-f, Frequency distributions of relative errors in annual roadside photovoltaic generation for fixed-tilt systems, comparing the averaging method with the matching method, for provinces in central China (**a**), northern China (**b**), northwestern China (**c**), eastern China (**d**), southern China (**e**) and northeastern China (**f**). Each provincial panel shows the histogram of relative errors in annual roadside PV generation, where the relative error is calculated by comparing the provincial estimate from the averaging method against that from the matching method. E, east; NE, northeast; N, north; NW, northwest; S, south; C, central. PV, photovoltaic; AH, Anhui; BJ, Beijing; CQ, Chongqing; FJ, Fujian; GD, Guangdong; GS, Gansu; GX, Guangxi; GZ, Guizhou; HA, Henan; HB, Hubei; HE, Hebei; HI, Hainan; HLJ, Heilongjiang; HN, Hunan; IM, Inner Mongolia; JL, Jilin; JS, Jiangsu; JX, Jiangxi; LN, Liaoning; NX, Ningxia; QH, Qinghai; SC, Sichuan; SD, Shandong; SH, Shanghai; SN, Shaanxi; SX, Shanxi; TJ, Tianjin; XJ, Xinjiang; XZ, Xizang; YN, Yunnan; ZJ, Zhejiang.

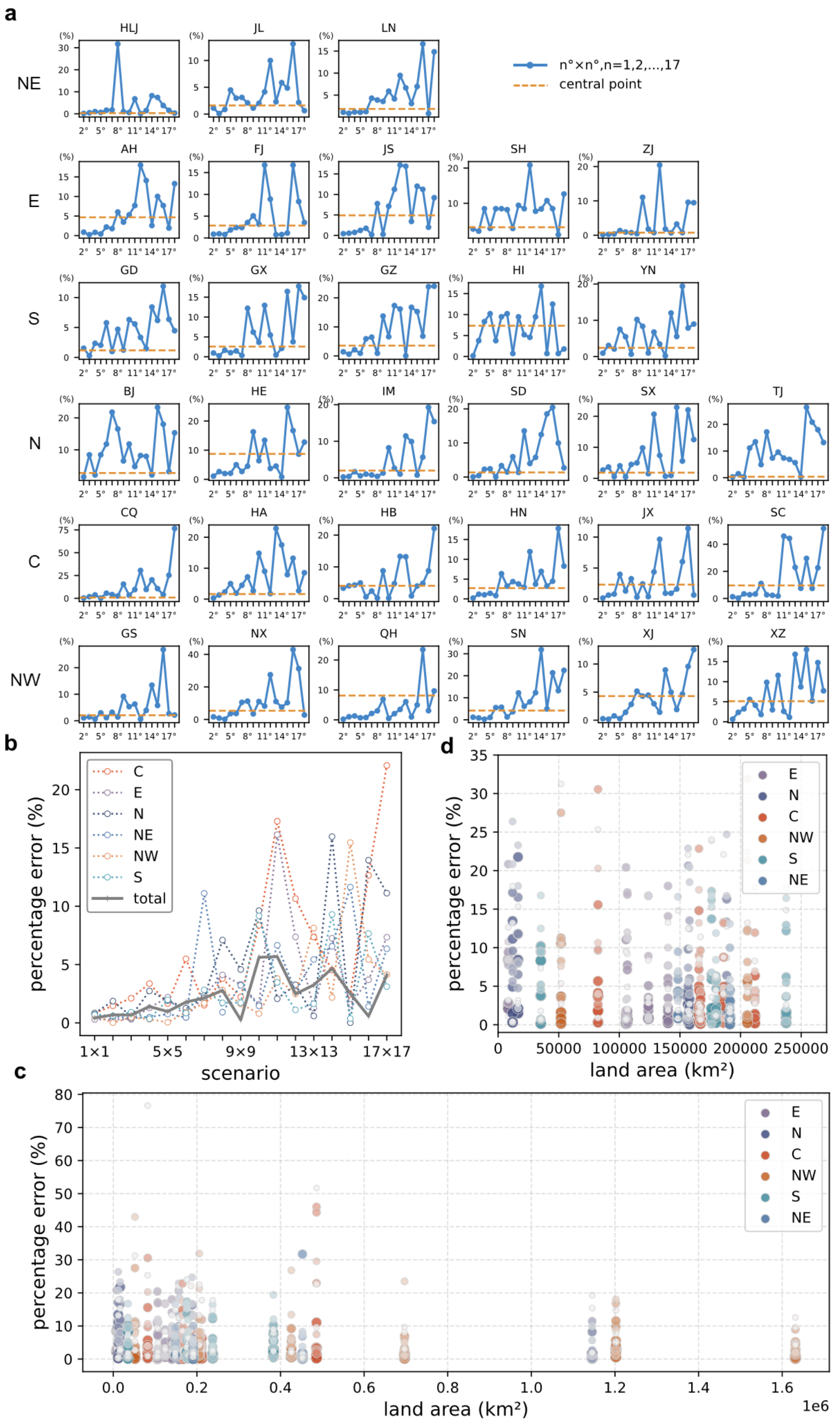

Supplementary Figure 9. Sensitivity analysis of provincial PV generation errors

with varying meteorological grid resolutions. a, Relative error in PV generation estimates across China's provinces, calculated from meteorological grid resolutions ranging from 1°×1° to 17°×17° (blue lines). The orange dashed lines represent errors from using only the provincial capital city for calculation. **b**, Relative error percentages of predicted roadside PV potentials at regional and national scales under various spatial resolutions. The grey solid line indicates the national-level results, while colored dashed lines denote the regional-level results. **c**, Scatter plot showing the relationship between provincial percentage errors and provincial land area under different grid resolutions. Each vertical set of dots represents a province, with dot shade and size indicating six groups of grid resolutions: ①1°×1° to 3°×3°, ②4°×4° to 6°×6°, ③7°×7° to 9°×9°, ④10°×10° to 12°×12°, ⑤13°×13° to 15°×15°, and ⑥16°×16°, 17°×17°, and the provincial capital-point scenario. Darker and larger dots at the bottom indicate finer resolutions. d, Zoom-in view of panel c, focusing on densely clustered points (land areas ≤250,000 km², errors ≤35%). Note: Increased fluctuations at coarser resolutions in panel a arise from fewer and unevenly distributed grid points, leading to greater variability in estimates, especially in smaller provinces. E, east; NE, northeast; N, north; NW, northwest; S, south; C, central. PV, photovoltaic; AH, Anhui; BJ, Beijing; CQ, Chongqing; FJ, Fujian; GD, Guangdong; GS, Gansu; GX, Guangxi; GZ, Guizhou; HA, Henan; HB, Hubei; HE, Hebei; HI, Hainan; HLJ, Heilongjiang; HN, Hunan; IM, Inner Mongolia; JL, Jilin; JS, Jiangsu; JX, Jiangxi; LN, Liaoning; NX, Ningxia; QH, Qinghai; SC, Sichuan; SD, Shandong; SH, Shanghai; SN, Shaanxi; SX, Shanxi; TJ, Tianjin; XJ, Xinjiang; XZ, Xizang; YN, Yunnan; ZJ, Zhejiang.

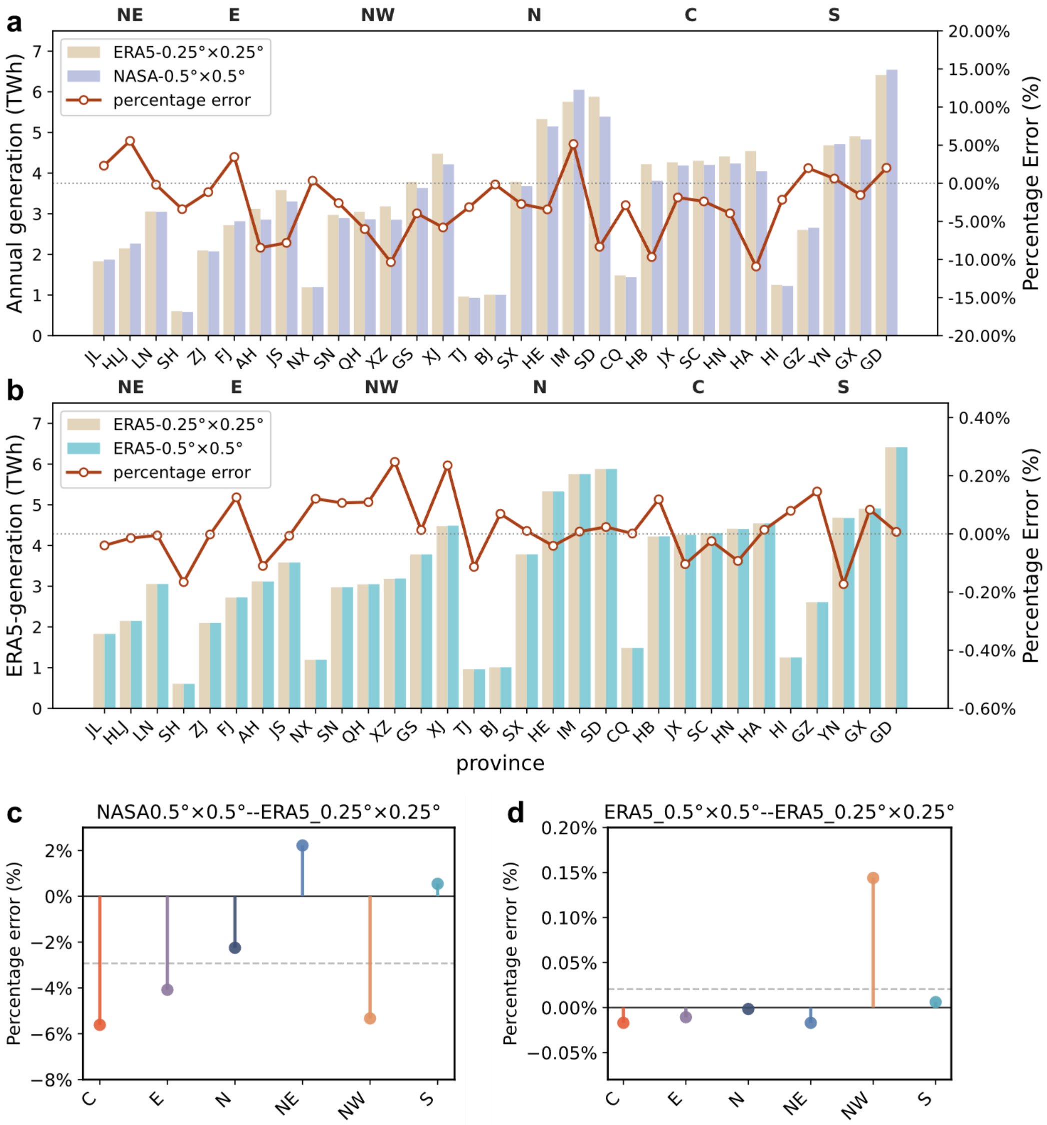

Supplementary Figure 10. Sensitivity of provincial PV generation estimates to meteorological inputs and resolution coarsening. a–b, Province-level annual roadside PV generation potentials and corresponding relative errors. Bars show provincial annual generation computed using three meteorological inputs: ERA5 0.25°×0.25°, ERA5 0.5°×0.5° (within-ERA5 coarsened setting), and NASA POWER 0.5°×0.5°. The line indicates the relative error (%) of each compared case relative to the ERA5 0.25°×0.25° baseline. **c–d**, Macro-region summaries (NE, N, NW, E, C, S) of the relative error (%) for each comparison, where (**c**) corresponds to NASA POWER 0.5°×0.5° vs ERA5 0.25°×0.25°, and (**d**) corresponds to ERA5 0.5°×0.5° vs ERA5 0.25°×0.25°. The dashed line indicates the national-scale relative error. E, east; NE, northeast; N, north; NW, northwest; S, south; C, central. PV, photovoltaic; AH, Anhui; BJ, Beijing; CQ, Chongqing; FJ, Fujian; GD, Guangdong; GS, Gansu; GX, Guangxi; GZ, Guizhou; HA,

Henan; HB, Hubei; HE, Hebei; HI, Hainan; HLJ, Heilongjiang; HN, Hunan; IM, Inner Mongolia; JL, Jilin; JS, Jiangsu; JX, Jiangxi; LN, Liaoning; NX, Ningxia; QH, Qinghai; SC, Sichuan; SD, Shandong; SH, Shanghai; SN, Shaanxi; SX, Shanxi; TJ, Tianjin; XJ, Xinjiang; XZ, Xizang; YN, Yunnan; ZJ, Zhejiang.

Supplementary Note 5. Investment cost estimation method for roadside PV projects

To clearly evaluate the investment scale and economic feasibility of roadside photovoltaic projects, we have developed a simple and transparent parametric cost estimation framework. The costs are explicitly divided into three main categories:

PV equipment cost, C_{pv}

This includes all investment related to PV system hardware and construction, such as PV modules, mounting structures, foundational electrical components (e.g., basic inverter and DC-side wiring), and site installation (excluding grid connection or reinforcement costs). Based on authoritative industry references (IEA PVPS and IRENA), we propose a reasonable cost range of approximately 0.50–0.80 USD/W [10,11].

Technical adaptation and interconnection cost, C_{ta}

This covers additional technical adaptation and interconnection infrastructure specifically required for roadside PV installations, such as DC–DC voltage step-up equipment or transformers, metering, protection, monitoring systems, and dedicated AC-side grid interconnection equipment. According to the NREL 2023 cost benchmarking report, electrical balance-of-system (BOS/EBOS) and inverter components contribute to system costs and have recently exhibited an increasing trend. Considering additional roadside-specific interface adaptations, we estimate this incremental cost at approximately 5–15% of system costs, equivalent to 0.03–0.10 USD/W [12]. Note this category is distinct from C_{pv}, as it specifically covers interface adaptation rather than primary PV equipment.

Grid reinforcement cost, C_{grid}

This refers to the additional costs incurred by the grid to accommodate new roadside PV capacity, including feeder reinforcement (conductors, voltage regulation,

protective relays), transformer expansions, and new or extended grid lines. The grid reinforcement cost is calculated using supplementary equation (7):

$$C_{\text{grid}} = k \cdot P_{pv} \cdot C_g \tag{7}$$

where the expansion factor k is defined in supplementary equation (8):

$$k = \frac{\max(P_{pv} - H, 0)}{P_{pv}} \tag{8}$$

Here P_{pv} represents the planned installed PV capacity (MW), and H represents the current grid hosting capacity (MW). The expansion factor k directly quantifies the proportion of additional grid reinforcement required. Specifically, k=0 means no grid reinforcement is needed, providing the easiest and most economical scenario. In contrast, k=100% indicates no available grid hosting capacity, thus requiring maximum grid reinforcement investment. We adopt a conservative unit grid reinforcement cost range of approximately 0.15–0.50 USD/W, considering typical international benchmarks for urban distribution network upgrades [13].

Thus, the total investment cost is expressed in supplementary equation (9):

$$C_{total} = C_{pv} + C_{ta} + C_{grid} \tag{9}$$

Supplementary Figure 11 presents the upper and lower boundaries of total investment costs for roadside PV deployment under varying grid expansion factors k, reflecting different levels of grid reinforcement requirements. The total investment cost is explicitly decomposed into three categories: PV equipment cost, grid-connection cost, and grid reinforcement cost. The shaded bands indicate the range of total investment resulting from uncertainty in individual cost components. A higher grid expansion factor k implies greater grid reinforcement requirements and thus a higher overall investment cost.

It should be emphasized that this cost estimation framework is designed to provide order-of-magnitude estimates and comparative scenario evaluations, clarifying economic scale and feasibility under different deployment conditions. However, it is based on linear simplifications of grid reinforcement costs, which in practice may have nonlinear or step-wise cost characteristics, especially when involving substation expansions or new grid construction. Furthermore, estimates of hosting capacity H are based on typical feeder limits or regional load characteristics as conservative proxies, which may differ from actual grid conditions influenced by real network structures and operational strategies. The unit cost ranges (PV equipment, technical adaptation, grid reinforcement) are based on typical industry references and may fluctuate according to

specific market conditions, construction practices, and local grid characteristics. The scope of this cost estimation framework is explicitly limited to initial capital investments, excluding taxation, financing, or project financial returns (such as LCOE or IRR). Therefore, while the framework provides useful national-scale technical-economic potential analyses and scenario comparisons, detailed feasibility studies based on actual local conditions, engineering designs, and specific grid data should be performed prior to real-world implementations.

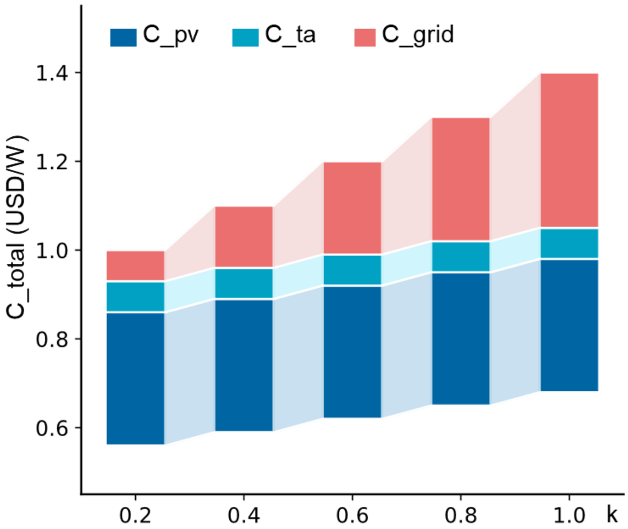

Supplementary Figure 11. Estimated investment cost ranges of roadside PV projects under varying grid expansion scenarios. Different coloured shaded blocks represent the three components of total investment cost: PV equipment cost (C_pv), technical adaptation and interconnection cost (C_ta), and grid reinforcement cost (C_grid). The height of each shaded block indicates the variation range of the corresponding cost component under each k value. k is the grid expansion factor, representing the share of additional grid reinforcement required to integrate the planned PV capacity. PV, photovoltaic.

Supplementary References

1. Xu, J., Guan, Y., Oldfield, J., Guan, D., & Shan, Y. China carbon emission accounts 2020-2021. *Appl. Energy* **360**, 122837 (2024).
2. Hersbach, H. et al. The ERA5 global reanalysis. *Q. J. R. Meteorol. Soc.* **146**, 1999–2049 (2020).

3. Sengupta, M. et al. The National Solar Radiation Database (NSRDB). *Renew. Sustain. Energy Rev.* **89**, 51–60 (2018).
4. Bessho, K. et al. An introduction to Himawari-8/9—Japan's new-generation geostationary meteorological satellites. *J. Meteorol. Soc. Jpn.* **94**, 151–183 (2016).
5. Ineichen, P., Perez, R., Seals, R., Maxwell, E. & Zelenka, A. Dynamic global-to-direct irradiance conversion models. *ASHRAE Trans.* **98**, 354–369 (1992).
6. Lu, N. & Qin, J. Optimization of tilt angle for PV in China with long-term hourly surface solar radiation. *Renew. Energy* **229**, 120741 (2024).
7. Klucher, T. M. Evaluation of models to predict insolation on tilted surfaces. *Sol. Energy* **23**, 111–114 (1979).
8. Polaris Solar Photovoltaic Network. Quick reference table for optimal instal lation angle, power generation, and annual utilization hours of photovoltaic power stations in various provinces and cities in China. https://guangfu.bjx.com.cn/news/20200708/1087109.shtml (2020).
9. Cheng, P., Liu, W. Q., Ma, J., Zhang, L. & Jia, L. Solar-powered rail transportation in China: Potential, scenario, and case. *Energy* **245**, 123221 (2022).
10. IEA PVPS. *Snapshot of Global Photovoltaic Markets 2024* (IEA Photovoltaic Power Systems Programme, 2024). https://iea-pvps.org
11. IRENA. *Renewable Power Generation Costs in 2023* (International Renewable Energy Agency, 2024). https://www.irena.org
12. Feldman, D., Ramasamy, V., Fu, R., Ramdas, A. & Margolis, R. *U.S. Solar Photovoltaic System and Energy Storage Cost Benchmarks, With Minimum Sustainable Price Analysis: Q1 2023* (National Renewable Energy Laboratory, NREL/TP-6A20-87303, 2023). https://www.nrel.gov
13. EPRI. *Impact Factors, Methods, and Considerations for Calculating and Applying Hosting Capacity* (Electric Power Research Institute, Palo Alto, CA, 2018). https://www.epri.com
