# Supplementary material for: National-level assessment of infrastructure-coupled roadside solar energy toward transportation decarbonization in China
Source: Nat Commun. 2026 Jun 2;17:7095. doi: 10.1038/s41467-026-73872-w (PMC13392230; doi:10.1038/s41467-026-73872-w)
Supplement: Supplementary file 2 — Reporting Summary [file 41467_2026_73872_MOESM2_ESM.pdf]

nature portfolio

Corresponding author(s): Ming Zhou, Jie Song

Last updated by author(s): Apr 23, 2026

Reporting Summary

Nature Portfolio wishes to improve the reproducibility of the work that we publish. This form provides structure for consistency and transparency in reporting. For further information on Nature Portfolio policies, see our Editorial Policies and the Editorial Policy Checklist.

Statistics

For all statistical analyses, confirm that the following items are present in the figure legend, table legend, main text, or Methods section.

n/a	Confirmed	
☐	☒	The exact sample size (n) for each experimental group/condition, given as a discrete number and unit of measurement
☐	☒	A statement on whether measurements were taken from distinct samples or whether the same sample was measured repeatedly
☐	☒	The statistical test(s) used AND whether they are one- or two-sided<br>*Only common tests should be described solely by name; describe more complex techniques in the Methods section.*
☒	☐	A description of all covariates tested
☒	☐	A description of any assumptions or corrections, such as tests of normality and adjustment for multiple comparisons
☐	☒	A full description of the statistical parameters including central tendency (e.g. means) or other basic estimates (e.g. regression coefficient) AND variation (e.g. standard deviation) or associated estimates of uncertainty (e.g. confidence intervals)
☐	☒	For null hypothesis testing, the test statistic (e.g. F, t, r) with confidence intervals, effect sizes, degrees of freedom and P value noted<br>*Give P values as exact values whenever suitable.*
☒	☐	For Bayesian analysis, information on the choice of priors and Markov chain Monte Carlo settings
☒	☐	For hierarchical and complex designs, identification of the appropriate level for tests and full reporting of outcomes
☐	☒	Estimates of effect sizes (e.g. Cohen's d, Pearson's r), indicating how they were calculated

Our web collection on statistics for biologists contains articles on many of the points above.

Software and code

Policy information about availability of computer code

Data collection	*Provide a description of all commercial, open source and custom code used to collect the data in this study, specifying the version used OR state that no software was used.*
Data analysis	All analyses were performed using custom Python code in Python 3.9. Spatial coupling between road points and gridded meteorological fields was implemented via a KD-tree nearest-neighbor matching algorithm, assigning each segmented road point to its nearest meteorological grid point. The PV resource-to-generation conversion and aggregation procedures follow the workflow described in the Methods and Supplementary Information. The custom analysis code is available at the GitHub repository: https://github.com/wuzyncepu/Revealing-infrastructure-coupled-solar-resources-for-transportation-decarbonization.

For manuscripts utilizing custom algorithms or software that are central to the research but not yet described in published literature, software must be made available to editors and reviewers. We strongly encourage code deposition in a community repository (e.g. GitHub). See the Nature Portfolio guidelines for submitting code & software for further information.

Data

Policy information about availability of data

All manuscripts must include a data availability statement. This statement should provide the following information, where applicable:

- Accession codes, unique identifiers, or web links for publicly available datasets
- A description of any restrictions on data availability
- For clinical datasets or third party data, please ensure that the statement adheres to our policy

The transportation network data used in this study were obtained from Open-StreetMap (https://www.openstreetmap.org/). Hourly meteorological data were obtained from the NASA Prediction Of Worldwide Energy Resources (POWER) project through the Data Access Viewer (https://power.larc.nasa.gov/data-access-viewer/). Provincial elec-tricity consumption and carbon emission data for the transportation sector were obtained from the China Carbon Accounting Database (CEADs) (https://www.ceads.net/data/). Additional data supporting the findings of this study are available within the paper and its Supplementary Information. No restrictions apply to the public datasets used in this study beyond the terms of use of the original data providers. Source data are provided with this paper.

Research involving human participants, their data, or biological material

Policy information about studies with human participants or human data. See also policy information about sex, gender (identity/presentation), and sexual orientation and race, ethnicity and racism.

Reporting on sex and gender	Not applicable. This study does not involve human participants, individual-level human data, or biological materials.
Reporting on race, ethnicity, or other socially relevant groupings	Not applicable. This study does not involve human participants or human demographic attributes.
Population characteristics	Not applicable. No human participants or individual-level population data were used.
Recruitment	Not applicable. No human participants were recruited.
Ethics oversight	Not applicable. This study does not involve human participants, human data, or biological materials; therefore ethics approval was not required.

Note that full information on the approval of the study protocol must also be provided in the manuscript.

Field-specific reporting

Please select the one below that is the best fit for your research. If you are not sure, read the appropriate sections before making your selection.

☐ Life sciences ☐ Behavioural & social sciences ☒ Ecological, evolutionary & environmental sciences

For a reference copy of the document with all sections, see nature.com/documents/nr-reporting-summary-flat.pdf

Ecological, evolutionary & environmental sciences study design

All studies must disclose on these points even when the disclosure is negative.

Study description	This study performs a national-scale, infrastructure-coupled environmental assessment of roadside photovoltaic deployment potential and associated carbon mitigation in China. It integrates geospatial transportation-corridor data with gridded meteorological inputs to estimate deployable capacity, annual electricity generation, carbon-reduction potential, and power-system impacts under multiple deployment scenarios, and compares the proposed high-resolution method with a province-level benchmark.
Research sample	The study uses existing datasets rather than biological samples. The "sample" consists of China's national railway and highway corridors (480,019 km) represented as geospatial line features, meteorological grid cells covering the study domain (e.g., NASA POWER at 0.5°×0.5°), and provincial administrative units (31 provinces) for aggregation and reporting.
Sampling strategy	No probabilistic sampling of organisms or participants was performed. We used full-coverage national corridor datasets and gridded meteorological products for the study domain. Road segments were processed and matched to meteorological grid cells using a KD-tree nearest-neighbor scheme; provincial/national estimates were obtained by aggregating along the full corridor network and across all relevant grid cells. Sample sizes are therefore determined by the spatial coverage and resolution of the underlying datasets.
Data collection	Transportation-corridor geospatial data were obtained from OpenStreetMap and preprocessed using QGIS v.3.32.0 and custom Python scripts, including data cleaning, segmentation, and exclusion of non-target categories such as bridge and tunnel segments. Hourly meteorological variables used for photovoltaic estimation were obtained from the NASA POWER project. Provincial transportation-sector electricity-consumption and carbon-emission data were obtained from the China Emission Accounts and Datasets (CEADs). Data preprocessing and analysis were implemented by the authors using custom Python code.
Timing and spatial scale	The analysis covers the national extent of China and is conducted across multiple spatial scales, including road-segment level

Timing and spatial scale	matching, meteorological grid cells (~0.5° × 0.5°), provincial aggregation (31 provinces), and six macro-regions. Meteorological inputs have hourly temporal resolution (8,760 hours per year) and were aggregated to annual estimates for reporting. The study period and temporal coverage follow the publicly available meteorological datasets used in the analysis (as specified in the Methods/ Supplementary Information).
Data exclusions	Data exclusions were applied only during preprocessing to ensure relevance and data quality. For example, non-target road/rail categories were removed, and bridge/tunnel segments were excluded to avoid double counting and to focus on deployable corridor surfaces. No additional exclusions were applied to the aggregated provincial/national estimates beyond the scenario definitions described in the manuscript and Supplementary Information.
Reproducibility	The computational workflow is fully reproducible. The full source code, processing scripts, and instructions to reproduce key results are publicly available via the repository/DOI stated in the Code Availability section, and the underlying numerical values for display items are provided in the Source Data file.
Randomization	Not applicable. This study is a computational assessment based on existing geospatial infrastructure and meteorological datasets; no organisms/participants were allocated to experimental groups.
Blinding	Not applicable. No participant-based data collection was conducted, and blinding is not relevant to this computational study.

Did the study involve field work? ☐ Yes ☒ No

Reporting for specific materials, systems and methods

We require information from authors about some types of materials, experimental systems and methods used in many studies. Here, indicate whether each material, system or method listed is relevant to your study. If you are not sure if a list item applies to your research, read the appropriate section before selecting a response.

Materials & experimental systems

n/a	Involved in the study
☒	☐ Antibodies
☒	☐ Eukaryotic cell lines
☒	☐ Palaeontology and archaeology
☒	☐ Animals and other organisms
☒	☐ Clinical data
☒	☐ Dual use research of concern
☒	☐ Plants

Methods

n/a	Involved in the study
☒	☐ ChIP-seq
☒	☐ Flow cytometry
☒	☐ MRI-based neuroimaging

Plants

Seed stocks	Not applicable. No plant materials or seed stocks were used in this study.
Novel plant genotypes	Not applicable. This study did not generate or use any plant genotypes.
Authentication	Not applicable. No plant materials were used, therefore no authentication procedures were required.
