# Supplementary material for: National-level assessment of infrastructure-coupled roadside solar energy toward transportation decarbonization in China
Source: Nat Commun. 2026 Jun 2;17:7095. doi: 10.1038/s41467-026-73872-w (PMC13392230; doi:10.1038/s41467-026-73872-w)
Supplement: Supplementary file 3 — Transparent Peer Review file [file 41467_2026_73872_MOESM3_ESM.pdf]

nature portfolio

Peer Review File

National-level assessment of infrastructure-coupled roadside solar energy toward transportation decarbonization in China

Corresponding Author: Dr Jianxiao Wang

This file contains all reviewer reports in order by version, followed by all author rebuttals in order by version.
Attachments originally included by the reviewers as part of their assessment can be found at the end of this file

Version 0:

Reviewer comments:

Reviewer #1

(Remarks to the Author)
This manuscript presents a well-structured assessment of the potential for DSPV deployment along China's national transportation network. The integration of detailed infrastructure data with hourly radiation inputs through a spatial matching framework addresses a relatively underexplored area. The study is relevant to ongoing discussions on infrastructure-integrated energy systems and low-carbon transport strategies. That said, several areas could benefit from further clarification, elaboration, or restructuring to maximize the impact and coherence of the work. My specific suggestions are as follows:

1. Introduction
The second paragraph of the introduction attributes the research focus on rooftop PV primarily to technical and structural constraints in other domains. While these are valid factors, the predominance of rooftop PV also stems from broader institutional and societal drivers—such as policy visibility, urban prioritization, and public engagement. In contrast, transportation corridor PV has remained underexplored not only due to spatial complexity, but also because it falls outside conventional energy planning frameworks. I suggest rephrasing this paragraph to more clearly reflect that this study does more than solve a technical challenge—it brings overdue attention to a strategically overlooked sector and reframes transport infrastructure as an active player in national decarbonization agendas.

2. Section 2.3
Although the manuscript already provides a valuable comparison between the proposed KD-tree-based approach (using station-derived radiation data) and traditional province-level aggregation, it may be worthwhile to briefly discuss how this method compares with an alternative raster-based approach that uses satellite-derived high-resolution surface radiation data. A concise comparison in terms of spatial accuracy, temporal continuity, and computational scalability could further clarify the rationale for the chosen method and highlight its advantages—particularly for nationwide infrastructure assessments that require stable hourly coverage and efficient integration with vector-based transport networks.

3. Section 2.6
The method section describes a rich scenario space of 27 installation configurations and 63 deployment scenarios. However, in the results, these scenarios are only briefly reflected as uncertainty ranges. I recommend expanding Section 2.6 into a more structured analysis of these scenarios, showing how key parameters—such as tracker type, deployment density, and tilt angle—affect outcomes like generation, carbon mitigation, and grid flexibility. This would clarify the distinction between baseline assumptions and design variability, while also reinforcing the empirical basis for the deployment recommendations made in the discussion.

4. Discussion
The discussion is structurally sound but remains overly general. While it outlines several future directions, it lacks grounding in specific policy frameworks, relevant literature, and quantitative references. In addition, the discussion does not draw on insights from existing studies in related fields, nor does it assess the potential impact or priority of each proposed pathway. Incorporating these elements could substantially enhance the strategic clarity and policy relevance of the conclusions.

(Remarks on code availability)

Reviewer #2

(Remarks to the Author)
Review Report – Nature Communications
Manuscript Title:
Revealing infrastructure-coupled solar resources for transportation decarbonization: High-resolution assessment of roadside PV potential in China
Comments to the Authors
Thank you for the opportunity to review this impactful and timely manuscript. The study presents a novel, high-resolution, spatiotemporal assessment of roadside photovoltaic (PV) deployment potential across China’s vast transport corridors. By integrating national rail and highway networks with dense meteorological grids and introducing a clustering-based spatial matching algorithm, the study offers a scalable and computationally efficient method for infrastructure-coupled PV analysis.
1. Noteworthy Contributions and Significance
- The manuscript presents an innovative spatial clustering approach that balances resolution and computational load, outperforming traditional grid- or point-based methods.
- It delivers the first comprehensive national-scale estimate of roadside PV potential in China with specific installable capacity, generation potential, and associated CO_2 reduction.
- The study demonstrates strong spatial heterogeneity in deployment potential and identifies regional hotspots like North and Central China.
- The results offer practical guidance for the co-optimization of transport and energy planning particularly relevant given China’s dual carbon goals.

Overall, the manuscript makes a substantial contribution to infrastructure-integrated energy research and will be of interest to researchers in renewable energy planning, transport decarbonization, and geospatial modeling.
2. Methodological Soundness
- The proposed clustering algorithm is both computationally novel and pragmatically relevant.
- The data coupling between road infrastructure and meteorological grids is comprehensive.
- The assumptions in baseline, moderate, and ideal scenarios are clearly defined.
- However, the manuscript would benefit from additional clarification on the validation strategy of the model outputs. For example:
- Was any cross-validation, sensitivity analysis, or benchmarking done?
- How robust are results to changes in clustering granularity or meteorological anomalies?

Having developed LiDAR-driven solar potential assessments at the city scale (An integrated approach for 3D solar potential assessment at the city scale, https://doi.org/10.3390/rs15235616) and worked on energy-efficiency-driven retrofitting using parametric simulation (Enhancement of the energy performance of an existing building using a parametric approach, https://doi.org/10.1061/JLEED9.EYENG-45), I find the current study particularly relevant. The study’s emphasis on spatial heterogeneity and infrastructure-coupled constraints resonates strongly with challenges I’ve addressed in dense urban environments. I encourage the authors to consider how their clustering framework might be adapted to account for vertical obstructions and real-world features, such as vegetation or roadside structures, which can critically influence PV performance, or discussion and limitation section.
3. Data Interpretation and Conclusions
- The interpretation is generally clear and the conclusions are well-supported.
- The authors emphasize carbon reduction and electricity offsets effectively.
- However, the socio-technical implementation barriers (e.g., legal rights-of-way, shading from vehicles or trees, road safety zones) are not fully addressed in the discussion. These factors may influence actual deployability and should be acknowledged.

Informed by my previous research, I see an opportunity to expand discussion on how roadside PV systems may benefit from solar design optimization in terms of orientation, tilt, and obstruction modeling—factors that are foundational in simulation-based solar studies and BIM-integrated energy models.
4. Suggestions for Improvement
a. Title and Abstract:
- The current title is descriptive but could better highlight the scale and novelty.
Suggested edit: "High-resolution national assessment of roadside PV integration potential along China's transport corridors."
- In the abstract, consider adding a brief note on model validation or caveats.

b. Methods:
- Include more detail on:
- Choice and sensitivity of clustering parameters (number of clusters)
- Uncertainty quantification or potential data biases
- Software or tools used for implementation

c. Figures and Tables:
- Improve accessibility by using colorblind-safe palettes.

- Add a summary table comparing baseline, moderate, and ideal scenario results.

d. Limitations and Broader Implications:
- Include a paragraph on potential economic feasibility, land-use conflicts, or policy considerations.
- Discuss generalizability: Can the method be extended to other countries with different road network topologies?

5. Reproducibility and Transparency
- The manuscript would benefit from a Data and Code Availability section, in line with Nature Communications' guidelines.
- Consider uploading source code or pseudocode for the clustering algorithm as supplementary material if not uploaded.

6. Structure and Organization
While the manuscript covers essential components, its overall structure would benefit from clearer segmentation following a more standardized scientific article format. Organizing content explicitly into sections such as "Introduction," "Methods," "Results," "Discussion," and "Conclusion" would enhance clarity, reader navigation, and alignment with broader publishing conventions in high-impact journals.

Conclusion
This is a well-crafted, policy-relevant, and technically sound manuscript. With minor revisions particularly around model validation, implementation context, and reproducibility it will make a valuable contribution to the literature on infrastructure-embedded renewable energy systems.

Recommendation: Minor revision.

Reviewer: Hassan Waqas

(Remarks on code availability)

Reviewer #3

(Remarks to the Author)
Abstract:
The statement "This output meets approximately 50.3% of the current electricity demand in the transportation sector and enables an annual carbon reduction of 33.62–143.97 $MtCO_2$, equivalent to 4.89–20.96% of transportation-related emissions" may be misleading. The authors should clarify which type of transportation demand is being referred to (e.g., electric vehicles, railways, maritime, etc.). Additionally, it should be stated whether efficiency losses and charging losses have been considered in the calculations.

Introduction:
The research question should be presented more explicitly. After the section addressing the knowledge gap, it would strengthen the manuscript to outline its structure. This is especially important given the unconventional order of chapters in the current version.

Formatting:

Line 121: indentation requires correction.

More broadly, indentation and formatting should be revised throughout the manuscript for consistency.

Equation 1:

The equation needs to be explained in more detail.

The authors should reference scientific literature that has adopted a similar strategy.

Why was 10 MW chosen as the reference size?

Have safety or correction coefficients been applied to account for module unavailability, distances between modules, and other practical constraints?

Scenario Configurations (Line 639):

The definition of scenarios should be supported by literature references or additional discussion.

Why were these specific scenarios chosen?

Were other potential scenarios discarded, and if so, for what reasons?

Grid Infrastructure Considerations:
It is not clear how the authors accounted for grid connection feasibility. Even with sufficient space and favorable irradiance, very remote areas may lack grid access, especially at the required connection capacities. The authors should clarify how

such infrastructure limitations were addressed in the study.

Line 340:

The terms “peak-to-valley differences” and “fluctuation mileage” should be better defined.

The inclusion of equations would improve clarity.

It is not immediately evident how the load profile is incorporated into the analysis—this should be explained in detail.

Line 414:
The current phrasing implies causality. Since correlation does not imply causation, the sentence should be revised accordingly.

Economic Considerations:
The manuscript would benefit significantly from an economic estimation of the required investments, both for power generation and the associated grid infrastructure. Even a rough estimate would provide valuable context on the feasibility and scale of the proposed solutions.

(Remarks on code availability)

Version 1:

Reviewer comments:

Reviewer #1

(Remarks to the Author)
The manuscript has improved a lot after the previous round of revisions. However, regarding the validation of the meteorological data resolution (specifically related to Comments R1.2 and R2.2), one critical issue remains unresolved that necessitates further clarification before publication.

While the authors have provided a sensitivity analysis in Supplementary Note 5.3 demonstrating that estimation errors increase as grid resolution coarsens from 0.5° to 17°, the current validation remains incomplete. The analysis treats the 0.5° NASA POWER dataset as a baseline without quantifying the potential deviation of this baseline itself.

Given the authors’ demonstration that "estimation errors systematically increase as the meteorological grid resolution becomes coarser", it is logically inferred that the 0.5° resolution may still retain non-negligible biases compared to finer-scale data （e.g., <0.1°）, particularly in regions with complex terrain or high infrastructure density. The justification relying solely on "computational efficiency" and "temporal continuity" is insufficient to dismiss the need for accuracy benchmarking at the sub-0.5° scale.

To ensure the robustness of the national-scale assessment, the authors should quantify the uncertainty of their 0.5° baseline. This does not require re-running the entire national model, but a comparative validation against a higher-resolution dataset (e.g., ERA5 at ~0.25° or satellite-derived irradiance) should be conducted for representative sample regions. This is essential to demonstrate that the 0.5° resolution provides a convergent and reliable estimate rather than merely a computationally convenient one.

(Remarks on code availability)

Reviewer #2

(Remarks to the Author)

(Remarks on code availability)
It's better to check with the other reviewer, who is more expert in codes and the running of codes.

Reviewer #3

(Remarks to the Author)
-

(Remarks on code availability)

Version 2:

Reviewer comments:

Reviewer #1

(Remarks to the Author)
The authors' ERA5-based analysis suffers from a fundamental logical circularity. By deriving coarse-resolution fields through spatial aggregation of finer-resolution data, the experiment primarily tests the internal mathematical consistency of spatial averaging, rather than the physical representativeness of inherently coarse-resolution datasets. In this design, high-resolution information is effectively leaked into the synthesized coarse fields.

Because the fine-to-coarse aggregation is itself a form of spatial smoothing, the analysis evaluates whether replacing a known fine-scale field with its spatial mean alters the results. This is not equivalent to the original question of whether native coarse-resolution meteorological data are sufficient. The central concern remains whether the original 0.5° dataset (e.g., NASA POWER), by construction, has already lost critical sub-grid variability associated with local topography, cloud processes, or microclimatic conditions.

The reported 0.0205% difference is therefore a predictable outcome of the smoothing-based experimental design and does not constitute evidence that the original 0.5° resolution is adequate. At a minimum, this methodological limitation should be explicitly acknowledged and the associated conclusions moderated. Ideally, validation against independently generated higher-resolution datasets or ground-based observations—particularly in topographically complex regions—would be required to meaningfully assess the magnitude of sub-grid biases.

(Remarks on code availability)

Version 3:

Reviewer comments:

Reviewer #1

(Remarks to the Author)
The revised manuscript now provides additional evidence that substantially strengthens the treatment of the resolution-sensitivity issue, and I consider the manuscript suitable for publication.

(Remarks on code availability)

Open Access This Peer Review File is licensed under a Creative Commons Attribution 4.0 International License, which permits use, sharing, adaptation, distribution and reproduction in any medium or format, as long as you give appropriate credit to the original author(s) and the source, provide a link to the Creative Commons license, and indicate if changes were

made.
In cases where reviewers are anonymous, credit should be given to 'Anonymous Referee' and the source.
The images or other third party material in this Peer Review File are included in the article’s Creative Commons license, unless indicated otherwise in a credit line to the material. If material is not included in the article’s Creative Commons license and your intended use is not permitted by statutory regulation or exceeds the permitted use, you will need to obtain permission directly from the copyright holder.
To view a copy of this license, visit https://creativecommons.org/licenses/by/4.0/

Manuscript: NCOMMS-25-57254-T

Authors: Zhaoyuan Wu, Jianxiao Wang, Lanyi Wei, Bo Li, Lin Chen, Lu Zhang, Daniel M. Kammen, Gengyin Li, Ming Zhou, Jie Song

Title: High-resolution national assessment of infrastructure coupled roadside solar energy toward transportation decarbonization

Contents

Reviewer 1 Comments to the Authors 2

Comment R1.1: 2

Comment R1.2: 3

Comment R1.3: 6

Comment R1.4: 8

Reviewer 2 Comments to the Authors 12

Comment R2.1: 12

Comment R2.2: 12

Comment R2.3: 17

Comment R2.4: 19

Comment R2.5: 20

Comment R2.6: 23

Comment R2.7: 25

Comment R2.8: 26

Comment R2.9: 27

Comment R2.10: 30

Reviewer 3 Comments to the Authors 31

Comment R3.1: 31

Comment R3.2: 32

Comment R3.3: 33

Comment R3.4: 33

Comment R3.5: 34

Comment R3.6: 37

Comment R3.7: 38

Comment R3.8: 40

Comment R3.9: 40

Manuscript: NCOMMS-25-57254-T

Authors: Zhaoyuan Wu, Jianxiao Wang, Lanyi Wei, Bo Li, Lin Chen, Lu Zhang, Daniel M. Kammen, Gengyin Li, Ming Zhou, Jie Song

Title: High-resolution national assessment of infrastructure coupled roadside solar energy toward transportation decarbonization

Response to Reviewers

We would like to thank the Reviewers for the insightful comments and for the thorough revision of the manuscript. We have carefully addressed all the comments and suggestions. The reviewers' comments are laid out below in *italicized font*, and the specific concerns have been numbered. These comments are all valuable and helpful for improving our article. According to the editor's and reviewers' comments, we have made extensive modifications to our manuscript, clarified our work's contributions, and provided comparisons against state-of-art methods to further verify the robustness and effectiveness of our approach. In this revised version, changes to our manuscript are all highlighted in BLUE. Point-by-point responses to comments of the three professional reviewers are listed below this letter.

Reviewer 1 Comments to the Authors

This manuscript presents a well-structured assessment of the potential for DSPV deployment along China's national transportation network. The integration of detailed infrastructure data with hourly radiation inputs through a spatial matching framework addresses a relatively underexplored area. The study is relevant to ongoing discussions on infrastructure-integrated energy systems and low-carbon transport strategies. That said, several areas could benefit from further clarification, elaboration, or restructuring to maximize the impact and coherence of the work. My specific suggestions are as follows:

Comment R1.1:

1. Introduction

The second paragraph of the introduction attributes the research focus on rooftop PV primarily to technical and structural constraints in other domains. While these are valid factors, the predominance of rooftop PV also stems from broader institutional and societal drivers—such as policy visibility, urban prioritization, and public engagement. In contrast, transportation corridor PV has remained underexplored not only due to spatial complexity, but also because it falls outside conventional energy planning frameworks. I suggest rephrasing this paragraph to more clearly reflect that this study does more than solve a technical challenge—it brings overdue attention to a strategically overlooked sector and reframes transport infrastructure as an active player in national decarbonization agendas.

Response to Comment R1.1:

Thank you for this insightful comment.

We fully agree that the predominance of rooftop PV arises not only from technical or structural constraints but also from institutional, societal, and policy-driven factors. To clarify this broader context and more accurately position the strategic contribution of our work, we have rephrased the second paragraph of the Introduction. Specifically, we now highlight that transportation corridor PV has remained underexplored largely because transportation infrastructure typically lies outside conventional energy planning frameworks. Our study explicitly seeks to bring overdue attention to this strategically neglected sector, reframing transport infrastructure as an active component of national decarbonization efforts.

In the revised manuscript, the second paragraph of the Introduction is reorganized as follows:

Currently, the integration of renewable energy in land-based transportation systems primarily centers on the deployment of photovoltaic (PV) systems along roadside corridors. This approach capitalizes on underutilized land adjacent to transportation infrastructure, seamlessly integrating PV generation with existing facilities to enhance spatial efficiency. While distributed PV potential has been rigorously assessed across spatial scales, from urban to national and global levels, existing research predominantly focuses on rooftop PV installations.

…

This bias stems not only from technical and structural constraints but also from institutional and sociopolitical factors, including preferential policies, urban-centric energy planning, and public engagement in visible urban projects. In contrast, the deployment of PV systems along transportation corridors remains significantly underexplored, owing to spatial complexities and technical uncertainties, and more critically, the systemic exclusion of transportation infrastructure from conventional energy planning paradigms. By redefining transportation corridors as active contributors rather than passive components of national decarbonization strategies, this study aims to bridge this strategic oversight, providing a timely and systematic evaluation of an underexplored yet high-potential renewable energy frontier.

Comment R1.2:

2. Section 2.3

Although the manuscript already provides a valuable comparison between the proposed KD-tree-based approach (using station-derived radiation data) and traditional province-level aggregation, it may be worthwhile to briefly discuss how this method compares with an alternative raster-based approach that uses satellite-derived high-resolution surface radiation data. A concise comparison in terms of spatial accuracy, temporal continuity, and computational

scalability could further clarify the rationale for the chosen method and highlight its advantages—particularly for nationwide infrastructure assessments that require stable hourly coverage and efficient integration with vector-based transport networks.

Response to Comment R1.2:

Thank you very much for this insightful suggestion. We fully agree with your point that higher-resolution raster-based datasets, such as satellite-derived high-resolution surface radiation products, could potentially enhance spatial accuracy. Indeed, one key advantage of our proposed method framework is its adaptability and flexibility to incorporate various data sources, including satellite-based datasets through appropriate downscaling or spatial aggregation approaches.

In the current study, we employed the widely used NASA POWER dataset because of its reliable and continuous hourly temporal coverage, stable performance in national-scale studies, and widespread acceptance in existing literature. Additionally, our core methodological contribution lies not primarily in the data itself, but rather in our novel spatial matching framework, which efficiently balances spatial accuracy, temporal continuity, and computational scalability, particularly suitable for extensive, nationwide infrastructure assessments.

Following your recommendation, we have made the following specific modifications in the revised manuscript:

1) We have now included a concise comparative discussion in Section 2.3, explicitly outlining the advantages and limitations of our KD-tree-based spatial matching method compared to raster-based alternatives.

Beyond province-level aggregation and point-wise mapping, an alternative approach is a raster-based pipeline using satellite-derived high-resolution surface radiation data. Raster-based methods typically offer finer nominal spatial resolution, allowing detailed capture of local gradients in solar radiation; however, they can introduce retrieval biases over complex terrain, adjacency effects due to heterogeneous land-cover types, and intermittent temporal gaps caused by cloud-masking processes. Furthermore, raster-vector overlay operations at hourly resolution significantly increase computational complexity at the national scale. In contrast, our proposed KD-tree spatial matching method, employing NASA POWER hourly radiation data, ensures continuous temporal coverage without gaps, providing robust stability for annual assessments and long-term scenario analysis. Additionally, by reducing hourly spatial computations to a one-time nearest-neighbor assignment, our KD-tree approach substantially decreases computational runtime and memory demands while maintaining sufficient spatial accuracy for corridor-level infrastructure planning. Thus, our method effectively balances spatial accuracy, temporal continuity, and computational scalability for nationwide assessments. A detailed summary table comparing these approaches is available in Supplementary Information.

2) We provide a clear summary table comparing major open-source radiation datasets,

explicitly highlighting their spatial resolution, temporal continuity, and computational requirements in **Supplementary Note 1.5 Comparison of Selected Open-source Radiation Datasets**:

Table S7 Comparison of selected open-source radiation datasets

Data Source	Spatial Resolution	Temporal Continuity	Data Availability	Key Meteorological Variables
NASA POWER (used here) **[1]**	0.5°×0.5° (~55 km)	Hourly, globally continuous (no gaps)	Open-source, global	Solar irradiance (GHI, DNI, DHI), temperature, wind, humidity
ERA5 (ECMWF Reanalysis) [2]	~0.25° × 0.25° (~28 km)	Hourly, globally continuous (no gaps)	Open-source, global	Solar irradiance (GHI), temperature, wind, pressure, precipitation
NSRDB (NREL, USA) [3]	~4 km (CONUS), ~10 km (Global)	Half-hourly or hourly, mostly continuous (gap-filled)	Open-source, primarily US/global partial coverage	Solar irradiance (GHI, DNI, DHI), cloud cover, meteorology
Himawari-8 Satellite [4]	~2–5 km (Asia-Pacific region)	10-min intervals, intermittent data gaps (cloud masking)	Partially open (processed products), Asia-Pacific only	Cloud cover, surface irradiance (GHI, DNI)
Surface Observations (ground stations)	Point measurements (station-level)	Variable (typically hourly), gaps possible	Limited & variable	Direct irradiance measurements, detailed weather variables

As illustrated in the table above, we provide a comparative summary of selected open-source solar radiation datasets. Among these, NASA POWER offers global coverage and continuous hourly temporal resolution, making it particularly suitable for national or continental-scale planning and scenario assessments. ERA5 features higher spatial resolution and a richer set of meteorological variables, commonly employed for detailed climate-energy coupling analyses, though involving higher computational overhead and data processing. The NSRDB provides high-resolution solar radiation data at half-hourly or hourly intervals primarily for the United States (CONUS), making it ideal for site-specific photovoltaic studies and model validation, though global coverage is partial. Himawari-8 satellite-derived radiation datasets represent extremely high spatial (~2–5 km) and temporal (10-minute intervals) resolutions within the Asia-Pacific region, offering significant advantages for detailed local-scale analyses (e.g., city-level or corridor-level studies). However, Himawari-8 datasets require more complex data processing, including cloud screening, interpolation, and gap-filling, leading to higher computational demands. Considering the national-scale objective of our study, we selected NASA POWER as the primary data source in this work.

Comment R1.3:

3. Section 2.6

The method section describes a rich scenario space of 27 installation configurations and 63 deployment scenarios. However, in the results, these scenarios are only briefly reflected as uncertainty ranges. I recommend expanding Section 2.6 into a more structured analysis of these scenarios, showing how key parameters—such as tracker type, deployment density, and tilt angle—affect outcomes like generation, carbon mitigation, and grid flexibility. This would clarify the distinction between baseline assumptions and design variability, while also reinforcing the empirical basis for the deployment recommendations made in the discussion.

Response to Comment R1.3:

Thank you very much for highlighting these critical points.

Thank you for this valuable recommendation. To fully address your comment, we have substantially expanded Section 2.6 into a structured and systematic analysis of the evaluated PV deployment scenarios. Specifically, compared to the previous version that primarily described scenario outcomes as general uncertainty ranges, the revised section now explicitly analyzes how critical parameters—including tracker type (fixed-tilt, tilted single-axis, dual-axis), spatial deployment scenarios (M1–M3), and tilt-angle deviations—systematically influence key outcomes such as installed capacity, electricity generation, carbon mitigation, and grid flexibility performance.

We have added a completely revised version of Fig. 8, clearly visualizing regional sensitivities and quantitative impacts of these parameters, including two newly considered operational metrics: the annual sum of daily peak-to-valley net load fluctuations and thermal plant ramping mileage. This structured sensitivity analysis explicitly highlights regional disparities in the effectiveness of tracking methods, particularly demonstrating substantial grid flexibility improvements in Southern and Central China when employing tilted single-axis tracking systems.

In the revised manuscript, the expanded Section 2.6 is shown as follows.

2.6 Structured Analysis of Roadside PV Deployment Scenarios

To systematically understand how critical design parameters affect roadside PV deployment potential, we conducted a structured sensitivity analysis considering tracker type (fixed-tilt, tilted single-axis, dual-axis) and spatial deployment rules (M1–M3, defined previously in Section 2.2). Collectively, these factors determine roadside PV system's installed capacities, electricity generation potential, carbon mitigation benefits, and grid flexibility performance across China's transportation corridors. The results are presented in Fig. 8.

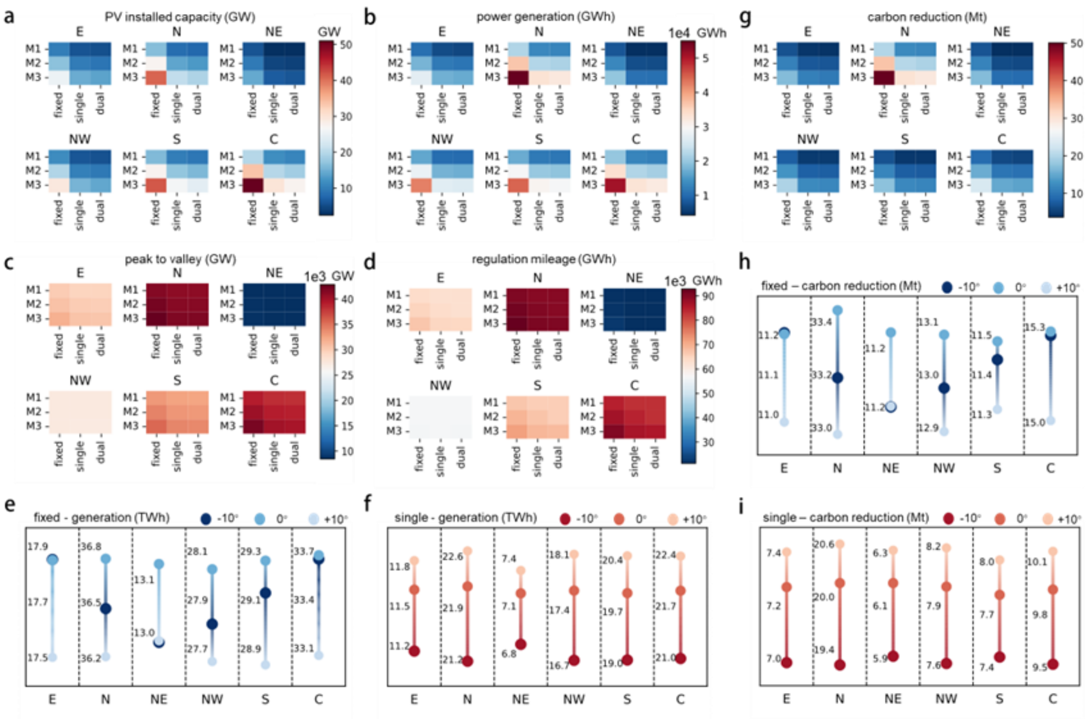

Fig. 8 | Sensitivity of tracker type and deployment rules (M1–M3) across macro-regions. Heatmaps (a–d) illustrate effects of tracker types (fixed-tilt, tilted single-axis, dual-axis; x-axis) and deployment scenarios M1–M3 on installable capacity (GW) (a), annual generation (TWh yr^{-1}) (b), annual sum of daily peak-to-valley net load spans (MW) (c), annual sum of daily thermal ramp mileage (MW·h) (d), and annual CO_2 abatement ($MtCO_2$ yr^{-1}) (g). for six macro-regions (E, N, NE, NW, S, C). Dot-range plots (e, f, h, i) summarize variability across deployment rules (M1–M3; markers from light to dark) for annual generation (TWh yr^{-1}) (e, f) and annual CO_2 abatement ($MtCO_2$ yr^{-1}) (h, i), for fixed-tilt (e, h) and tilted single-axis (f, i) tracker types, respectively. The baseline scenario (single-axis tracking, 24% module efficiency, moderate width/M2) balances technical performance, deployability, and practical realism. Full numeric details are provided in Supplementary Information 3.2 and 3.3.

Our analyses indicate that fixed-tilt PV systems consistently offer the highest installed capacities and annual generation potentials nationwide due to their efficient land utilization and simplified structural requirements. As shown in Fig. 8a and Fig. 8b, fixed-tilt configurations achieve approximately 90% greater installable capacity and roughly 60% higher annual generation compared to dual-axis tracking systems. Although tracking methods, particularly tilted single-axis, can enhance annual electricity output through improved solar alignment, their larger land footprints, higher structural complexity, and increased installation costs often outweigh these yield gains in constrained corridor scenarios. In particular, tilted single-axis systems offer only modest incremental gains, about 8% in installed capacity and 3-5% in annual generation, relative to fixed-tilt systems, suggesting limited justification for their widespread deployment in densely packed transportation corridors. However, tilted single-axis tracking

demonstrates significant operational benefits in grid flexibility, particularly in regions with dense transportation networks and pronounced solar variability, such as Southern and Central China. Specifically, single-axis tracking markedly reduces annual peak-to-valley net load fluctuations and thermal power plant ramping mileage compared to fixed-tilt systems (Fig. 8c–d). These operational improvements enhance grid stability and integration capability, illustrating a critical advantage for tracking systems in managing intermittent renewable generation.

Regional performance disparities are shaped by latitude-dependent solar incidence angles and corridor spatial constraints. In high-latitude areas, such as Northeast China, fixed-tilt systems exhibit the greatest relative advantages due to steeper optimal tilt angles and lower winter sun elevations. Conversely, low-latitude provinces such as Guangdong and Yunnan, characterized by consistently high solar angles and limited roadside areas, exhibit minimal performance differences between fixed-tilt and tracking systems. Midlatitude regions, including North and Central China, present favorable conditions balancing solar availability and spatial flexibility, reinforcing fixed-tilt systems' overall advantages in capacity deployment and electricity generation.

Further sensitivity analyses of tilt angle deviations reveal asymmetric impacts across regions. For fixed-tilt configurations, deviations of +10° from optimal angles result in greater generation losses compared to -10° deviations, particularly in mid- and low-latitude regions. This asymmetry arises from flatter tilt angles better aligning with dominant summer sun positions, suggesting slight under-tilting could be advantageous. In contrast, high-latitude regions exhibit more symmetrical losses with both positive and negative tilt deviations due to steeper baseline angles and balanced seasonal solar paths. Evaluations of tilted single-axis systems, which currently derive their tilt angles from fixed-tilt optimal designs, indicate room for further optimization. All analyzed scenarios underperform relative to theoretical maxima, notably in high-irradiance regions such as Northwest China, where optimized tilt angles could enhance generation by more than 5%. These insights underscore the importance of region-specific geometric optimizations, especially given that even small percentage improvements can translate into significant absolute benefits in large-scale deployments.

Comment R1.4:

4. Discussion

The discussion is structurally sound but remains overly general. While it outlines several future directions, it lacks grounding in specific policy frameworks, relevant literature, and quantitative references. In addition, the discussion does not draw on insights from existing studies in related fields, nor does it assess the potential impact or priority of each proposed pathway.

Incorporating these elements could substantially enhance the strategic clarity and policy relevance of the conclusions.

Response to Comment R1.4:

Thank you very much for this constructive and insightful suggestion. We fully acknowledge your concerns regarding the generality of the original discussion and have made substantial revisions to enhance its strategic clarity, policy relevance, and empirical grounding.

Specifically, we have revised the discussion section by clearly referencing specific policy frameworks and integrating insights from existing international studies related to infrastructure-integrated photovoltaics. For instance, we explicitly referenced China's recent policy documents, including the "2024–2025 Energy Conservation and Carbon Reduction Action Plan," the guidelines jointly issued by ten ministries for promoting energy and transportation integration (April 2025), and the newly released "Green-power Direct Connection" policy (May 2025). These policies directly support the deployment of roadside photovoltaics (PV) along transportation corridors, providing strong justification for our recommended regional deployment strategies.

Moreover, we have integrated comparative insights from existing international research and practices, explicitly mentioning the European Union's "Solar Highways" concept and successful projects like the Rijkswaterstaat's EU LIFE project on integrated PV-noise barriers, and the U.S. Federal Highway Administration's Right-of-Way Alternative Use Framework, which facilitated the Massachusetts Department of Transportation's (MassDOT) successful highway PV deployments. Additionally, experiences from Switzerland's Federal Roads Office (ASTRA) and Austria's ASFINAG in market-driven PV deployment along highways were incorporated to provide clear comparative benchmarks and enrich the strategic relevance of our recommendations.

Furthermore, we now explicitly assess the potential impacts and prioritize future research pathways. Drawing on previous studies and policy experiences, we propose immediate prioritization of demand-driven scenarios in densely populated and economically active regions, such as Eastern and Southern China, where integration of roadside PV with energy storage and charging infrastructure offers significant near-term impact. In contrast, resource-driven regions, notably Northwest and North China, characterized by abundant solar resources and available land, are identified for strategic medium-to-long-term priority deployment, including the exploration of large-scale roadside PV combined with green-power direct connections and other linear infrastructure (transmission corridors, high-speed rails, and pipelines).

The specific modifications in the revised manuscript are shown as follows.

The region-specific deployment strategies proposed here closely align with China's recent renewable-energy policies and infrastructure development plans. The Chinese government has explicitly encouraged the integration of photovoltaic systems with transportation infrastructure,

emphasizing their coordinated deployment with electric vehicle charging facilities and local renewable energy utilization [5][6]. Additionally, recent policy developments facilitating direct renewable energy supply to targeted consumers have further strengthened support for roadside PV projects [7]. Such policy support provides clear guidance and implementation frameworks for roadside PV expansion. Based on regional resource conditions and energy demand profiles, North and Northwest China, with ample roadside space and superior solar irradiation, present opportunities for large-scale roadside PV deployment. In contrast, Eastern and Southern China, facing constrained land availability, should prioritize roadside PV coupled with storage and charging facilities to optimize local renewable energy utilization, while Central China might adopt a hybrid approach balancing generation capacity and grid flexibility needs. Insights from international experiences and existing studies also provide clear benchmarks and strategic guidance for roadside PV deployment in China. The European Union's "Solar Highways" initiative, exemplified by the Rijkswaterstaat's EU LIFE project, has successfully demonstrated integrated PV noise barriers along highways [8][9]. In the United States, the Federal Highway Administration's Right-of-Way Alternative Use Framework enabled the Massachusetts Department of Transportation's successful public-private partnerships for roadside PV installations [10]-[12]. Furthermore, highway operators like Switzerland's ASTRA and Austria's ASFINAG have successfully promoted market-driven PV projects along highways, accumulating valuable technical standards and viable business models [13]-[15]. These international policy practices and project experiences offer valuable benchmarks for China. Concurrently, China is rapidly enhancing its technical standards and policy frameworks for roadside PV deployment, including ongoing standardization efforts by the National Development and Reform Commission, ensuring critical technical support and policy guarantees for scaling up roadside PV.

Building on these insights and policy contexts, we clearly identify potential impacts and prioritize future research pathways. In the near term, densely populated, economically active regions in Eastern and Southern China with intensive energy demand but limited land availability should prioritize integrated roadside PV deployment combined with energy storage and charging infrastructure. Studies addressing dynamic factors, such as climate change impacts, electrification trends, and sector coupling (e.g., logistics, cold chains, urban mobility)—should be prioritized in these regions to effectively meet growing local renewable energy needs and infrastructure demands. In the medium-to-long term, regions with abundant solar resources and available land, particularly Northwest and North China, are suitable for higher-standard, large-scale roadside PV deployments, including studies on integrating green-power direct connection schemes and cross-regional transmission networks. Additionally, exploring synergies between roadside PV and other linear infrastructures (such as transmission corridors, high-speed railways, and pipelines) in resource-rich regions represents a strategically significant long-term research direction. Clearly defining these priorities based on demand-driven and resource-

driven contexts allows for more precise and effective deployment strategies, supporting China's targeted, economically feasible energy transition. However, practical deployment of roadside PV also faces several important constraints. Economic feasibility remains a critical consideration, particularly regarding upfront investment costs, the cost-effectiveness of grid infrastructure upgrades, and long-term maintenance expenditures. Land-use conflicts, such as competition with agriculture, ecological conservation areas, or urban development, represent additional complexities requiring careful spatial planning and coordinated policy management. Furthermore, while our analytical framework is broadly applicable, extending this methodology to other countries with different road network topologies would require adaptations to account for region-specific infrastructure standards, data availability, and regulatory contexts. Addressing these economic, land-use, and generalizability challenges through future research will be critical to facilitating broader adoption and successful implementation of infrastructure-coupled renewable energy systems.

Reviewer 2 Comments to the Authors

Thank you for the opportunity to review this impactful and timely manuscript. The study presents a novel, high-resolution, spatiotemporal assessment of roadside PV deployment potential across China's vast transport corridors. By integrating national rail and highway networks with dense meteorological grids and introducing a KD-tree spatial matching algorithm, the study offers a scalable and computationally efficient method for infrastructure-coupled PV analysis.

Comment R2.1:

1. Noteworthy Contributions and Significance

- The manuscript presents an innovative spatial clustering approach that balances resolution and computational load, outperforming traditional grid- or point-based methods.

- It delivers the first comprehensive national-scale estimate of roadside PV potential in China with specific installable capacity, generation potential, and associated CO_2 reduction.

- The study demonstrates strong spatial heterogeneity in deployment potential and identifies regional hotspots like North and Central China.

- The results offer practical guidance for the co-optimization of transport and energy planning particularly relevant given China's dual carbon goals.

Overall, the manuscript makes a substantial contribution to infrastructure-integrated energy research and will be of interest to researchers in renewable energy planning, transport decarbonization, and geospatial modeling.

Response to Comment R2.1:

Thank you very much for your positive and encouraging feedback. We greatly appreciate your recognition of our study's contribution and relevance.

Comment R2.2:

2. Methodological Soundness

- The proposed clustering algorithm is both computationally novel and pragmatically relevant.

- The data coupling between road infrastructure and meteorological grids is comprehensive.

- The assumptions in baseline, moderate, and ideal scenarios are clearly defined.

- However, the manuscript would benefit from additional clarification on the validation strategy of the model outputs. For example:

- Was any cross-validation, sensitivity analysis, or benchmarking done?

- How robust are results to changes in clustering granularity or meteorological anomalies?

Having developed LiDAR-driven solar potential assessments at the city scale (An integrated approach for 3D solar potential assessment at the city scale, https://doi.org/10.3390/rs15235616) and worked on energy-efficiency-driven retrofitting using parametric simulation (Enhancement of the energy performance of an existing building using a parametric approach, https://doi.org/10.1061/JLEED9.EYENG-4546), I find the current study particularly relevant. The study's emphasis on spatial heterogeneity and infrastructure-coupled constraints resonates strongly with challenges I've addressed in dense urban environments. I encourage the authors to consider how their clustering framework might be adapted to account for vertical obstructions and real-world features, such as vegetation or roadside structures, which can critically influence PV performance, or discussion and limitation section.

Response to Comment R2.2:

Thank you very much for highlighting these critical methodological considerations. To clearly address your suggestions, we have structured our responses as follows:

1) Impact of meteorological grid resolution:

We fully agree on the importance of evaluating how meteorological grid granularity affects our estimation results. To explicitly address this point, we conducted a systematic sensitivity analysis to examine how varying the meteorological grid resolution from the baseline of 0.5°×0.5° to coarser grids (ranging from 1°×1° up to 17°×17°) influences our provincial-level PV generation estimates. The results, clearly presented in a newly added figure in the Supplementary Information, demonstrate that coarser grid resolutions substantially increase local estimation errors, particularly in smaller provinces. However, our selected baseline resolution (0.5°×0.5°) effectively balances accuracy and computational feasibility, resulting in robust and stable estimates across all regions.

Regarding benchmarking, to our knowledge, this study represents the first high-resolution, nationwide assessment specifically focused on roadside PV deployment potential, annual electricity generation, and carbon mitigation capacity across all regions in China. Currently, no clearly established external benchmark exists for direct comparison in this unique application. Therefore, the sensitivity analyses presented (including the aforementioned grid resolution analysis) primarily quantify deviations from our baseline scenario (0.5°×0.5° grid resolution) rather than comparing with external benchmarks. We explicitly clarified this limitation in the revised manuscript to ensure transparency.

In the revised manuscript, we have expanded the Supplementary Information (SI §S5.3) to provide a detailed sensitivity analysis examining how meteorological grid resolution affects PV generation estimates, shown as follows.

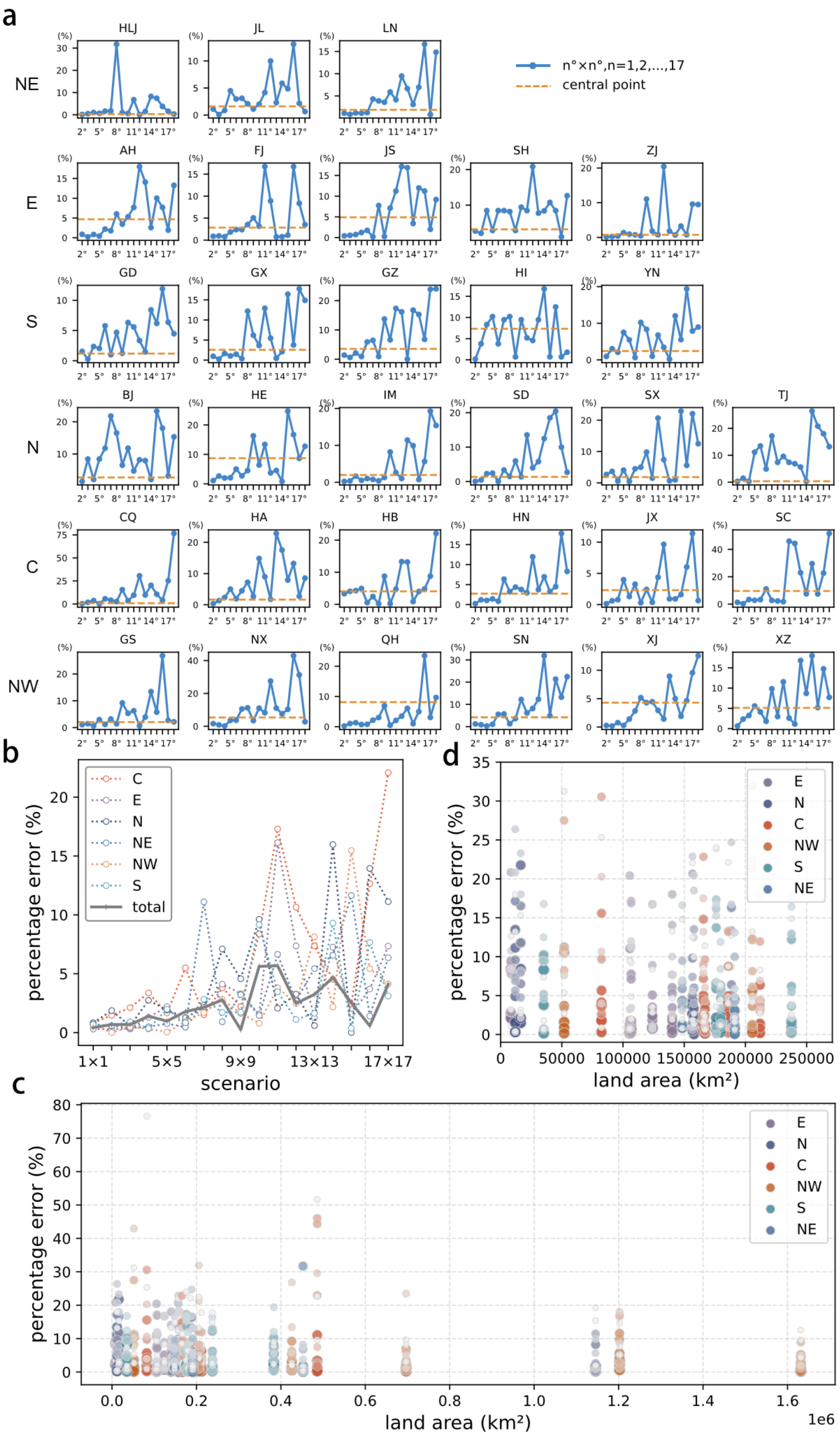

Fig. S14 Sensitivity analysis of provincial PV generation errors with varying meteorological grid resolutions. a, Relative error in PV generation estimates across China's provinces, calculated from meteorological grid resolutions ranging from 1°×1° to 17°×17° (blue lines). The orange

dashed lines represent errors from using only the provincial administrative center point for calculation. b, Relative error percentages of predicted roadside PV potentials at regional and national scales under various spatial resolutions. The grey solid line indicates the national-level results, while colored dashed lines denote the regional-level results. c, Scatter plot showing the relationship between provincial percentage errors and provincial land area under different grid resolutions. Each vertical set of dots represents a province, with dot shade and size indicating six groups of grid resolutions: ①1°×1° to 3°×3°, ②4°×4° to 6°×6°, ③7°×7° to 9°×9°, ④10°×10° to 12°×12°, ⑤13°×13° to 15°×15°, and ⑥16°×16°, 17°×17°, and the central-point scenario. Darker and larger dots at the bottom indicate finer resolutions. d, Zoom-in view of panel c, focusing on densely clustered points (land areas ≤250,000 km², errors ≤35%). Note: Increased fluctuations at coarser resolutions in panel a arise due to fewer and unevenly distributed grid points, causing significant variability in estimates, especially in smaller provinces.

Table S14 The number of grids corresponding to different resolutions

Grid resolution	Number of grids	Grid resolution	Number of grids
0.5°×0.5°	4133	9°×9°	24
1°×1°	1071	10°×10°	22
2°×2°	301	11°×11°	18
3°×3°	144	12°×12°	18
4°×4°	91	13°×13°	15
5°×5°	63	14°×14°	13
6°×6°	47	15°×15°	14
7°×7°	36	16°×16°	12
8°×8°	31	17°×17°	9

We assume that higher-resolution meteorological grids provide more accurate PV potential estimates due to their finer spatial representation of local climatic conditions. Thus, we select the 0.5°×0.5° meteorological grid scenario as the ground truth for this analysis. As illustrated in Fig. S14, estimation errors systematically increase as the meteorological grid resolution becomes coarser, highlighting the significant sensitivity of PV assessments to spatial input resolution. Notably, while the maximum national-level relative error remains relatively modest (up to 5.7%), provincial-level errors are substantially greater, reaching as high as 76.6%. Regionally, Southern China (S) generally exhibits lower and more stable relative errors, whereas Central China (C) demonstrates significantly higher and more volatile errors across varying grid resolutions. Small provinces (e.g., Beijing, Shanghai, Tianjin, and Hainan) consistently show elevated and fluctuating errors due to their limited geographic extent, which intensifies sensitivity to minor shifts in meteorological grid points at coarser resolutions. Such provinces inherently have fewer representative grid points at lower spatial resolutions, making their estimates particularly susceptible to spatial misalignment and localized meteorological variability.

Additionally, we identified pronounced extreme errors in provinces characterized not only by small area but also by significantly uneven road-network distributions. For example, Chongqing, Ningxia, and particularly Sichuan, exhibit pronounced errors under coarser resolutions.

Sichuan, notably located at the boundary of distinct solar resource zones and characterized by an extremely uneven distribution of transportation infrastructure, demonstrates exceptionally high sensitivity starting from grid resolutions as coarse as 10°×10° degrees. Conversely, provinces with larger land areas and relatively homogeneous distributions of both transportation networks and solar resources (e.g., Inner Mongolia, Zhejiang, Heilongjiang) exhibit greater robustness, maintaining consistently low estimation errors (approximately 2%) even at moderately coarse resolutions (up to approximately 9°×9° degrees). Interestingly, despite extensive land areas, provinces in Northwest China demonstrate sensitivity similar to smaller provinces, primarily due to uneven transport infrastructure distribution and large latitudinal spans, causing substantial variability in local solar radiation.

These results highlight a crucial methodological insight: the suitability of spatial meteorological resolution is intricately tied to provincial land area, spatial homogeneity of infrastructure, and local variability in solar resource distribution. Our chosen baseline of 0.5°×0.5° resolution emerges as a well-balanced compromise, maintaining computational efficiency while effectively minimizing spatial aggregation biases. For future practical deployments and detailed local-scale assessments, adopting finer meteorological data (e.g., high-resolution satellite-derived datasets) or integrating detailed local analyses (e.g., LiDAR-based solar resource mapping) will be critical for accurately capturing localized meteorological variability and infrastructure characteristics. Such practices are particularly essential in smaller provinces, or regions characterized by highly heterogeneous infrastructure distribution, to effectively minimize estimation errors and guide more reliable infrastructure planning and PV project implementation.

2) Comprehensive sensitivity analysis of key parameters:

We have explicitly conducted a detailed sensitivity analysis to systematically evaluate how key model parameters influence our results. Specifically, we examined four major categories of parameters: (a) **tracking method** (fixed-tilt, tilted single-axis tracking, dual-axis tracking), (b) **deployment density** (conservative, moderate, optimistic), (c) **spatial width scenarios** (M1–M3, representing variations in roadside available area), and (d) **tilt angles** (optimal angle, ±10° deviations). By systematically combining these parameter variations, we generated a comprehensive set of 63 distinct scenarios (as clearly documented in Supplementary Information Note 3), evaluating their effects on three primary outcomes: **installed PV capacity**, **annual electricity generation**, and **carbon mitigation potential**.

Specifically, the detailed scenario configurations and parameter combinations are explicitly documented in **Supplementary Information Note 3 (Scenario Settings and Parameter Matrix)**, including clearly defined scenario boundaries and assumptions. Additionally, we con-

ducted an in-depth sensitivity analysis focused on the influence of different PV tracking technologies and deployment widths across China's six macro-regions (East, North, Northeast, Northwest, South, Central). These detailed regional sensitivity results are clearly presented in the newly added **Figure 8** of the revised manuscript, which explicitly visualizes the impact of varying parameters on installed capacity, annual generation, and grid operational indicators such as peak-to-valley load differences and thermal ramping mileage.

3) Vertical obstructions and site-specific considerations:

Thank you for highlighting the importance of accounting for vertical obstructions (e.g., vegetation, roadside structures, buildings), which critically influence actual PV performance. We fully acknowledge this limitation explicitly in our revised Discussion section, clarifying that our assessment provides a theoretical upper bound of roadside PV potential, representing relatively optimistic resource estimates without shading considerations. Inspired by your suggested references—such as LiDAR-based solar potential assessments at the city scale (https://doi.org/10.3390/rs15235616) and parametric modeling for urban energy performance enhancement (https://doi.org/10.1061/JLEED9.EYENG-4546), we further recommended that future site-specific studies incorporate detailed obstruction modeling methods (e.g., LiDAR or field surveys) to refine estimates of actual generation potential.

In the revised manuscript, we have explicitly addressed the uncertainty and biases introduced by local vertical obstructions and site-specific factors in Methods (Section 5.6), as shown below:

…

2) Local shading effects not explicitly modeled: Due to the national-scale and high-resolution nature of this study, explicit modeling of local shading factors, including vegetation, roadside structures, buildings, safety barriers, and dynamic shading from vehicles, poses significant methodological challenges. Such local-scale shading and obstruction effects require extensive, site-specific data and significantly increase computational complexity, making nationwide modeling currently infeasible. Consequently, our estimates represent theoretical upper bounds of roadside PV potential, providing relatively optimistic resource assessments without considering shading losses. Actual photovoltaic generation at specific project sites may therefore be lower than the technical estimates presented here. To overcome these limitations, future project-level analyses should explicitly incorporate detailed obstruction-aware workflows, such as LiDAR-based 3D solar potential mapping [16] or parametric design optimization methods [17], to precisely quantify and mitigate shading-related performance losses.

Comment R2.3:

3. Data Interpretation and Conclusions

- The interpretation is generally clear and the conclusions are well-supported.

- The authors emphasize carbon reduction and electricity offsets effectively.

- However, the socio-technical implementation barriers (e.g., legal rights-of-way, shading from vehicles or trees, road safety zones) are not fully addressed in the discussion. These factors may influence actual deployability and should be acknowledged.

Informed by my previous research, I see an opportunity to expand discussion on how roadside PV systems may benefit from solar design optimization in terms of orientation, tilt, and obstruction modeling—factors that are foundational in simulation-based solar studies and BIM-integrated energy models.

Response to Comment R2.3:

Thank you very much for this insightful suggestion. We fully agree that socio-technical barriers, such as legal rights-of-way restrictions, shading impacts from vehicles, vegetation, or other structures, and road safety considerations, critically influence the practical implementation of roadside PV systems. Following your recommendation, we have explicitly addressed these socio-technical barriers within our revised Discussion section. Moreover, we have emphasized the importance of detailed solar design optimization, including panel orientation, tilt angle refinement, and comprehensive shading and obstruction modeling, as key elements for maximizing system performance and economic viability. We clearly indicate that future project-level analyses and feasibility studies should explicitly incorporate these detailed considerations to ensure successful implementation.

In the revised manuscript, we expanded the discussion section to explicitly acknowledge and discuss socio-technical implementation barriers, shown as follows.

…

However, practical deployment of roadside PV also faces several important constraints. Economic feasibility remains a critical consideration, particularly regarding upfront investment costs, the cost-effectiveness of grid infrastructure upgrades, and long-term maintenance expenditures. Land-use conflicts, such as competition with agriculture, ecological conservation areas, or urban development, represent additional complexities requiring careful spatial planning and coordinated policy management. Socio-technical barriers present significant challenges to the practical deployment of PV systems along transportation corridors. These include legal limitations on rights-of-way, dynamic shading effects from vegetation, vehicular traffic, and adjacent structures, as well as road safety buffer zones that limit available installation space. Additionally, maximizing system performance and economic returns necessitates rigorous solar design optimization, encompassing precise panel orientation, site-specific tilt angle calibration, and comprehensive shading/obstruction modeling. Future studies should integrate these socio-technical factors and detailed designs into project feasibility assessments and planning frame-

works, ensuring comprehensive evaluation of deployment viability. Furthermore, while our analytical framework is broadly applicable, extending this methodology to other countries with different road network topologies would require adaptations to account for region-specific infrastructure standards, data availability, and regulatory contexts. Addressing these economic, land-use, socio-technical, and generalizability challenges through future research will be critical to facilitating broader adoption and successful implementation of infrastructure-coupled renewable energy systems.

Comment R2.4:

4. Suggestions for Improvement

a. Title and Abstract:

- The current title is descriptive but could better highlight the scale and novelty.

Suggested edit: "High-resolution national assessment of roadside PV integration potential along China's transport corridors."

- In the abstract, consider adding a brief note on model validation or caveats.

Response to Comment R2.4:

Thank you very much for your valuable suggestion regarding the title and abstract. Following your recommendation, we have revised the title to better emphasize both the **national scale** and the **novelty** of our study. Specifically, we adopted your suggested wording ("High-resolution national assessment") to directly highlight the spatial scope and methodological resolution. Meanwhile, to more explicitly underline our key innovation, the integration of roadside PV deployment with transportation infrastructure and its significant implications for transportation decarbonization, we slightly refined the phrasing to:

"High-resolution national assessment of infrastructure-coupled roadside solar energy toward transportation decarbonization"

Additionally, we agree that mentioning model validation or caveats briefly in the abstract can enhance clarity. We have now added a concise statement about the model validation approach and key caveats to ensure readers are appropriately informed upfront. **The revised Abstract is shown as follows:**

Integrating renewable energy into land-based transportation infrastructure represents a promising pathway for decarbonization. However, existing studies have not yet systematically quantified the national potential of roadside photovoltaic (PV) deployment in terms of generation capability and the associated carbon reduction, particularly for extensive systems, such as highways and railways. Additionally, assessments often rely on geographical partitioning and fail to incorporate high-resolution meteorological data with geospatial road layouts, which is

required for accurate estimation. This study develops a high-resolution spatiotemporal assessment model that couples 480,019 km of national railways and highways with 4,133 meteorological grids. A KD-tree spatial matching algorithm is proposed to balance computational efficiency and estimation accuracy, offering significant advantages over both geographical partitioning methods and computationally intensive point-by-point mapping. The analysis reveals that roadside PV systems in China's transportation corridors could support 40.91-202.84 GW of installable capacity, generating 57.5-239.2 TWh annually across scenarios. This output meets approximately 50.3% of the current electricity demand in the transportation sector and enables an annual carbon reduction of 33.62–143.97 MtCO_2, equivalent to 4.89-20.96% of transportation-related emissions. The results further reveal strong spatial heterogeneity. Regions such as North China and Central China offer the highest deployment potential because of dense networks and high irradiation, whereas Northwest China, despite its low demand, can act as a solar export hub. Model robustness is supported by sensitivity analyses, and key uncertainties related to spatial data resolution and infrastructure constraints are acknowledged. These findings offer scientific guidance for regionalized infrastructure planning and the co-optimization of transportation and energy systems.

Comment R2.5:

b. Methods:

- Include more detail on:

- Choice and sensitivity of clustering parameters (number of clusters)

- Uncertainty quantification or potential data biases

- Software or tools used for implementation

Response to Comment R2.5:

We appreciate your invaluable suggestions and comments. As you recommended, we have addressed your three specific points: 1) choice and sensitivity of spatial matching parameters (number of clusters), 2) uncertainty quantification and potential data biases, and 3) software and tools used, in detail as follows:

1. Choice and sensitivity of spatial matching parameters (number of clusters)

Thank you for pointing out the need to clarify our methodological approach. To clarify, the method used in this study is not a clustering method requiring the selection of a number of clusters. Instead, we employed a nearest-neighbor matching approach based on a KD-Tree algorithm, directly associating each road segment with its closest meteorological grid point from a predefined set of 4,133 grid points across China. Thus, the number of meteorological grid points (4,133) was determined solely by the fixed resolution (~0.5°) of the NASA POWER dataset, and was not determined by a clustering procedure.

Given this methodological context, there is no clustering step or associated clustering parameter (such as the "number of clusters") involved. Nevertheless, **we conducted detailed comparative analyses in response to Comment R2.2**, explicitly evaluating differences in results obtained from varying meteorological data resolutions (e.g., raster-based high-resolution vs. KD-tree matching), and these detailed comparisons are presented clearly in the Supplementary Information.

In the revised manuscript, we have clarified our methodological approach at the beginning of Section 5.2, as follows:

5.2 Matching Road Points with Meteorological Grid Points

To account for spatial variability in resource conditions along transportation corridors, we developed a spatial matching method using a KD-Tree nearest-neighbor algorithm, directly associating each road point with the nearest meteorological grid point. Road infrastructure data from OpenStreetMap were first segmented into discrete spatial points at defined intervals (e.g., 1 km segments), and then matched spatially with a set of 4,133 meteorological grid points covering China, derived from the NASA POWER dataset (spatial resolution ~0.5°, approximately 50 km). Each segmented road point was individually assigned meteorological parameters, such as solar irradiance, temperature, and other hourly meteorological conditions, from its nearest meteorological grid point, based on geographical proximity determined via KD-Tree nearest-neighbor search. This method involves no clustering step or explicit determination of cluster numbers, ensuring computational efficiency and spatial precision at a national scale. The detailed steps of this matching algorithm are as follows:

2. Uncertainty quantification or potential data biases

Thank you very much for emphasizing the importance of explicitly addressing uncertainty and potential data biases. To directly respond to your suggestion, we have added a dedicated subsection (**Methods 5.6: Uncertainty quantification and potential data biases**) clearly outlining the primary sources of uncertainty and data biases, which include:

5.6 Uncertainty quantification and potential data biases

Several potential sources of uncertainty and data biases should be noted in interpreting the results of this study:

1) Meteorological data resolution and spatial aggregation effects: The NASA POWER meteorological dataset used in this analysis has a spatial resolution of approximately 0.5° (roughly 50 km). This relatively coarse resolution may not adequately capture localized variations in solar irradiance, particularly due to local cloud cover dynamics or complex terrain features. Such limitations could introduce local-scale estimation biases in the assessment of photovoltaic generation potential. Additionally, spatial aggregation effects, systematically analyzed within our study, may further result in localized inaccuracies, especially in areas characterized

by dense road networks or significant geographical variability.

2) Local shading effects not explicitly modeled: Due to the national-scale and high-resolution nature of this study, explicit modeling of local shading factors, including vegetation, roadside structures, buildings, safety barriers, and dynamic shading from vehicles, poses significant methodological challenges. Such local-scale shading and obstruction effects require extensive, site-specific data and significantly increase computational complexity, making nationwide modeling currently infeasible. Consequently, our estimates represent theoretical upper bounds of roadside PV potential, providing relatively optimistic resource assessments without considering shading losses. Actual photovoltaic generation at specific project sites may therefore be lower than the technical estimates presented here. To overcome these limitations, future project-level analyses should explicitly incorporate detailed obstruction-aware workflows, such as LiDAR-based 3D solar potential mapping [16] or parametric design optimization methods [17], to precisely quantify and mitigate shading-related performance losses.

3) Infrastructure data accuracy: The road infrastructure data utilized in this study, sourced from OpenStreetMap (OSM), are widely accessible and frequently used but may have varying completeness and accuracy across regions. Such variability can affect the precision of estimated deployable areas and photovoltaic installation capacities along road networks.

Although these factors introduce some local-level uncertainties, they are unlikely to significantly impact the key findings and national-level conclusions of this study—that substantial photovoltaic deployment potential exists within China's transportation infrastructure. Future research and project-level implementation efforts should incorporate higher-resolution satellite-derived irradiance datasets, site-specific measurements, and detailed local shading analyses to further mitigate these uncertainties and enhance the accuracy of deployment strategies.

3. Software or tools used for implementation

In this paper, our analytical framework comprises three primary computational modules:

1) Roadside Resource Assessment: Estimating available roadside areas and determining potential installable photovoltaic capacity.

2) Solar Resource Simulation: Calculating plane-of-array irradiance and optimal panel efficiency based on site-specific tilt angles.

3) Hourly Generation Calculation: Combining estimated installed capacities with hourly solar resource data to compute hourly power output throughout the year (8760 hours).

All analyses and calculations were implemented using Python (version 3.9). Specifically, we employed widely-used scientific computing libraries such as NumPy, Pandas, and SciPy for data processing and analysis; PVlib for solar irradiance modeling and performance calculations; and Geopy for geospatial data handling and distance computations. **We have made all source**

code, detailed documentation, and example datasets publicly accessible through our GitHub repository:

https://github.com/wuzyncepu/Revealing-infrastructure-coupled-solar-resources-for-transportation-decarbonization.

This repository clearly provides separate Python scripts for each analytical module, detailed instructions on input/output formats, and datasets to facilitate replication, validation, and further methodological transparency.

Comment R2.6:

c. Figures and Tables:

- Improve accessibility by using colorblind-safe palettes.

- Add a summary table comparing baseline, moderate, and ideal scenario results.

Response to Comment R2.6:

Thank you for highlighting these important points. To improve accessibility and clarity, we have revised the color schemes used in our figures to ensure they are colorblind-friendly, thus enhancing readability for all readers.

Additionally, we have included a clear and concise summary table in the Supplementary Information that explicitly compares key results (installed capacity, annual generation, and carbon reduction potential) across baseline, moderate, and ideal deployment scenarios.

The added summary table is shown as follows.

Table S11 Regional comparison of roadside PV potential installed capacity

Tracking Methods	Region	PV Installed Capacity (GW)		
		M1	M2	M3
Fixed-tilt	E	9.94	16.84	25.39
	N	16.32	27.34	40.95
	NE	5.80	9.63	14.35
	NW	11.40	19.39	29.28
	S	16.03	27.56	41.86
	C	19.78	33.73	51.02
	total	79.27	134.49	202.84
Single-axis tracking	E	5.74	9.73	14.66
	N	8.30	13.91	20.83
	NE	2.64	4.38	6.53
	NW	5.98	10.18	15.38
	S	10.03	17.23	26.17
	C	11.61	19.79	29.93
	total	44.29	75.22	113.50

Dual-axis tracking	E	5.35	9.06	13.67
	N	7.66	12.82	19.21
	NE	2.46	4.04	6.03
	NW	5.52	9.38	14.18
	S	9.23	15.87	24.11
	C	10.70	18.25	27.60
	total	40.91	69.43	104.79

Table S12 Regional comparison of roadside PV generation potential

Tracking Methods	Region	PV Generation (TWh)		
		M1	M2	M3
Fixed-tilt	E	10.54	17.85	26.90
	N	21.94	36.74	55.01
	NE	7.91	13.13	19.57
	NW	16.47	28.03	42.34
	S	17.02	29.26	44.45
	C	19.74	33.68	50.95
	Total	93.62	158.70	239.23
Single-axis tracking	E	6.86	11.63	17.53
	N	13.26	22.20	33.25
	NE	4.33	7.19	10.71
	NW	10.37	17.66	26.68
	S	11.61	19.96	30.33
	C	12.85	21.92	33.16
	Total	59.28	100.56	151.66
Dual-axis tracking	E	6.58	11.16	16.82
	N	12.89	21.56	32.29
	NE	4.25	6.99	10.41
	NW	10.15	17.28	26.12
	S	11.24	19.33	29.36
	C	12.36	21.08	31.90
	Total	57.47	97.39	146.90

Table S13 Regional comparison of roadside PV carbon mitigation potential

Tracking Methods	Region	Carbon Mitigation Potential (Mt)		
		M1	M2	M3
Fixed-tilt	E	6.62	11.20	16.87
	N	19.98	33.45	50.07

	NE	6.79	11.28	16.81
	NW	7.74	13.08	19.68
	S	6.70	11.49	17.43
	C	8.98	15.29	23.11
	total	56.80	95.78	143.97
Single-axis tracking	E	4.30	7.27	10.96
	N	12.05	20.17	30.19
	NE	3.73	6.19	9.22
	NW	4.73	8.00	12.04
	S	4.56	7.82	11.86
	C	5.84	9.95	15.03
	total	35.20	59.39	89.31
Dual-axis tracking	E	4.12	6.97	10.50
	N	11.72	19.59	29.34
	NE	3.66	6.02	8.97
	NW	4.62	7.80	11.75
	S	4.40	7.56	11.46
	C	5.62	9.58	14.48
	total	34.15	57.52	86.50

Comment R2.7:

d. Limitations and Broader Implications:

- Include a paragraph on potential economic feasibility, land-use conflicts, or policy considerations.

- Discuss generalizability: Can the method be extended to other countries with different road network topologies?

Response to Comment R2.7:

Thank you for these important suggestions. We have addressed your recommendations by explicitly adding a concise discussion within the manuscript's "Discussion" section regarding potential economic feasibility, land-use conflicts, and relevant policy considerations. Specifically, we now clearly acknowledge potential economic constraints, such as investment costs associated with roadside PV infrastructure and necessary grid upgrades, as well as potential conflicts arising from competing land-use demands (e.g., agriculture, ecological conservation, urban development).

Additionally, we explicitly address the broader generalizability of our method by discussing its applicability to other regions or countries with different road network topologies. We clarify that the proposed spatial matching framework is flexible and can be adapted readily to

diverse contexts, provided suitable geospatial data and meteorological inputs are available. We also highlight necessary adjustments or considerations that may be required when extending this approach internationally, such as differences in infrastructure standards, policy frameworks, or market conditions.

The specific revisions made in the Discussion section are as follows:

…

However, the practical deployment of roadside PV systems also confronts multifaceted important constraints. Economic feasibility remains a critical consideration, encompassing critical dimensions including upfront investment costs, the cost-effectiveness of grid infrastructure upgrades, and long-term maintenance expenditures. Land-use conflicts, such as competition with agriculture, ecological conservation areas, or urban development, introduce additional layers of complexity requiring careful spatial planning and coordinated policy management. Socio-technical barriers, including legal rights-of-way limitations, potential shading from vegetation, vehicles, or nearby structures, and road safety buffer zones, further complicate practical deployment. Additionally, detailed solar design optimization, addressing panel orientation, precise tilt angle settings, and comprehensive shading or obstruction modeling, is crucial for maximizing system performance and economic returns. Future site-specific studies should explicitly incorporate these socio-technical factors and detailed design optimizations into project feasibility assessments and planning. Furthermore, while our analytical framework is broadly applicable, extending this methodology to other countries with different road network topologies would require adaptations to account for region-specific infrastructure standards, data availability, and regulatory contexts. Addressing these economic, land-use, socio-technical, and generalizability challenges through future research will be critical to facilitating broader adoption and successful implementation of infrastructure-coupled renewable energy systems.

Comment R2.8:

5. Reproducibility and Transparency

- The manuscript would benefit from a Data and Code Availability section, in line with Nature Communications' guidelines.

- Consider uploading source code or pseudocode for the clustering algorithm as supplementary material if not uploaded.

Response to Comment R2.8:

Thank you very much for highlighting the importance of reproducibility and transparency. We have already made our source code publicly available via GitHub. The full Python codebase, including the KD-tree spatial matching algorithm and associated data-processing scripts developed for this study, can be accessed at the following public repository:

https://github.com/wuzyncepu/Revealing-infrastructure-coupled-solar-resources-for-transportation-decarbonization.

We have explicitly referenced this GitHub repository in a newly added "Code availability" section of the manuscript, clearly fulfilling the code transparency requirements.

Data availability

The network data is available on the official website of the open-source mapping platform OpenStreetMap (OSM) [18]. The meteorological data is available on the NASA POWER project platform [1]. Scenario setting parameters and the main computational results are provided in detail in the Supplementary Information. Additional supporting datasets, including regional classifications and carbon emission baselines, are also provided in the Supplementary Information.

Code availability

The main source code developed for this study, including the KD-tree spatial matching algorithm and the associated data-processing scripts necessary to reproduce the reported results, is publicly available at the following GitHub repository: **https://github.com/wuzyncepu/Revealing-infrastructure-coupled-solar-resources-for-transportation-decarbonization**. The repository is openly accessible without restriction.

Comment R2.9:

6. Structure and Organization

While the manuscript covers essential components, its overall structure would benefit from clearer segmentation following a more standardized scientific article format. Organizing content explicitly into sections such as "Introduction," "Methods," "Results," "Discussion," and "Conclusion" would enhance clarity, reader navigation, and alignment with broader publishing conventions in high-impact journals.

Response to Comment R2.9:

Thank you for your valuable suggestion regarding manuscript structure and clarity. We have carefully followed your advice and explicitly reorganized the manuscript to align with the standardized scientific format typically recommended by high-impact journals. Specifically, we have clearly segmented the content into distinct sections: Introduction, Methods, Results, Discussion, and Conclusion.

Previously, our "Discussion and Conclusion" sections were combined. In response to your recommendation, we have now separated them into two clearly defined sections:

3. Discussion

This study contributes to a deeper understanding of how spatial structure, infrastructure form, and solar resource distribution jointly shape the deployment potential and system impact of distributed PV in transportation systems. Three key structural mechanisms emerge. First, pronounced spatial decoupling exists between PV resource availability and transportation electricity demand, driven by the mismatch between solar irradiance gradients and freight activity concentrations. Second, estimation biases in PV potential are systematically linked to spatial aggregation effects, particularly in regions with concentrated or elongated road networks and steep irradiance gradients. Third, the choice of system configuration, such as the tracking method and tilt angle, interacts nonlinearly with local resource conditions, highlighting the need for deployment strategies that reflect both spatial heterogeneity and geometric sensitivity.

The region-specific deployment strategies proposed here closely align with China's recent renewable-energy policies and infrastructure development plans. The Chinese government has explicitly encouraged the integration of photovoltaic systems with transportation infrastructure, emphasizing their coordinated deployment with electric vehicle charging facilities and local renewable energy utilization [5][6]. Additionally, recent policy developments facilitating direct renewable energy supply to targeted consumers have further strengthened support for roadside PV projects [7]. Such policy support provides clear guidance and implementation frameworks for roadside PV expansion. Based on regional resource conditions and energy demand profiles, North and Northwest China, with ample roadside space and superior solar irradiation, present opportunities for large-scale roadside PV deployment. In contrast, Eastern and Southern China, facing constrained land availability, should prioritize roadside PV coupled with storage and charging facilities to optimize local renewable energy utilization, while Central China might adopt a hybrid approach balancing generation capacity and grid flexibility needs.

Insights from international experiences and existing studies also provide clear benchmarks and strategic guidance for roadside PV deployment in China. The European Union's "Solar Highways" initiative, exemplified by the Rijkswaterstaat's EU LIFE project, has successfully demonstrated integrated PV noise barriers along highways [8][9]. In the United States, the Federal Highway Administration's Right-of-Way Alternative Use Framework enabled the Massachusetts Department of Transportation's successful public-private partnerships for roadside PV installations [10]-[12]. Furthermore, highway operators like Switzerland's ASTRA and Austria's ASFINAG have successfully promoted market-driven PV projects along highways, accumulating valuable technical standards and viable business models [13]-[15]. These international policy practices and project experiences offer valuable benchmarks for China. Concurrently, China is rapidly enhancing its technical standards and policy frameworks for roadside PV deployment, including ongoing standardization efforts by the National Development and Reform Commission, ensuring critical technical support and policy guarantees for scaling up roadside PV.

Building on these insights and policy contexts, we clearly identify potential impacts and prioritize future research pathways. In the near term, densely populated, economically active regions in Eastern and Southern China with intensive energy demand but limited land availability should prioritize integrated roadside PV deployment combined with energy storage and charging infrastructure. Studies addressing dynamic factors, such as climate change impacts, electrification trends, and sector coupling (e.g., logistics, cold chains, urban mobility), should be prioritized in these regions to effectively meet growing local renewable energy needs and infrastructure demands. In the medium-to-long term, regions with abundant solar resources and available land, particularly Northwest and North China, are suitable for higher-standard, large-scale roadside PV deployments, including studies on integrating green-power direct connection schemes and cross-regional transmission networks. Additionally, exploring synergies between roadside PV and other linear infrastructures (such as transmission corridors, high-speed railways, and pipelines) in resource-rich regions represents a strategically significant long-term research direction. Clearly defining these priorities based on demand-driven and resource-driven contexts allows for more precise and effective deployment strategies, supporting China's targeted, economically feasible energy transition. However, practical deployment of roadside PV also faces several important constraints. Economic feasibility remains a critical consideration, particularly regarding upfront investment costs, the cost-effectiveness of grid infrastructure upgrades, and long-term maintenance expenditures. Land-use conflicts, such as competition with agriculture, ecological conservation areas, or urban development, represent additional complexities requiring careful spatial planning and coordinated policy management. Furthermore, while our analytical framework is broadly applicable, extending this methodology to other countries with different road network topologies would require adaptations to account for region-specific infrastructure standards, data availability, and regulatory contexts. Addressing these economic, land-use, and generalizability challenges through future research will be critical to facilitating broader adoption and successful implementation of infrastructure-coupled renewable energy systems.

4. Conclusion

This study demonstrates how spatially explicit, infrastructure-coupled energy modeling can inform the design of next-generation energy systems that are both low-carbon and spatially coherent. The proposed methodology offers a scalable template for harmonizing distributed renewables with built environments, particularly in geospatially complex and rapidly urbanizing regions. As countries pursue carbon neutrality alongside infrastructure expansion, embedding spatial intelligence into energy planning is critical to unlocking synergies, preventing integration bottlenecks, and maximizing system-level carbon efficiency. Taken together, this research provides a robust methodological foundation and a clear strategic perspective for embedding distributed solar deployment into national infrastructure systems, offering pathways

toward more flexible, regionally balanced, and carbon-efficient energy transitions.

Comment R2.10:

Conclusion: This is a well-crafted, policy-relevant, and technically sound manuscript. With minor revisions particularly around model validation, implementation context, and reproducibility it will make a valuable contribution to the literature on infrastructure-embedded renewable energy systems.

Response to Comment R2.10:

Thank you very much for your positive assessment and encouraging feedback regarding our manuscript. We appreciate your recognition of its policy relevance and technical robustness. In line with your suggestions, we have carefully addressed the recommended minor revisions, explicitly improving the manuscript in areas of model validation, clearly articulating the implementation context, and enhancing reproducibility.

Reviewer 3 Comments to the Authors

Comment R3.1:

Abstract:

The statement "This output meets approximately 50.3% of the current electricity demand in the transportation sector and enables an annual carbon reduction of 33.62–143.97 MtCO$_2$*, equivalent to 4.89–20.96% of transportation-related emissions" may be misleading. The authors should clarify which type of transportation demand is being referred to (e.g., electric vehicles, railways, maritime, etc.). Additionally, it should be stated whether efficiency losses and charging losses have been considered in the calculations.*

Response to Comment R3.1:

Thank you for highlighting this issue.

The transportation electricity demand referenced in our analysis specifically includes electricity consumption related to **electrified railway transportation (including high-speed and conventional railway)** and **road transportation infrastructure (e.g., electric vehicle charging)**.

The carbon emissions data for the transportation sector used in this study are sourced from the China Emission Accounts and Datasets (CEADs). This dataset calculates direct CO_2 emissions from final fossil fuel consumption across 17 fossil fuel types, explicitly excluding indirect emissions associated with electricity or heat consumption, as these indirect emissions are already accounted for at the generation side (power plants). The emissions figures therefore represent direct final energy consumption, naturally excluding considerations of efficiency or charging losses in electricity generation, transmission, and distribution.

In response to your comment, we have made two primary modifications in the revised manuscript to clearly define the scope of transportation electricity demand considered and explicitly address whether efficiency and charging losses were accounted for:

1) We have revised the Abstract explicitly as follows:

This output meets approximately 50.3% of the current electricity demand for electrified railway and road transportation infrastructure, and enables an annual carbon reduction of 33.62-143.97 $MtCO_2$, equivalent to 4.89-20.96% of transportation-related emissions (covering railway, road, waterway, aviation, pipeline, multimodal transportation, warehousing, and postal services). These emission estimates represent direct emissions from final energy consumption and do not include efficiency losses in electricity generation or vehicle charging processes.

2) We have added explicit clarification in the main text (Section 2.5) where transportation-related emissions and electricity demand are first introduced:

The transportation-sector electricity demand referenced here specifically includes electricity consumption associated with electrified railway systems (both high-speed and conventional railways) and road transportation infrastructure (e.g., electric vehicle charging along highways).

Maritime, aviation, and pipeline transportation are excluded from this analysis. Provincial transportation-related carbon emissions data used in this analysis are sourced from the China Emission Accounts and Datasets (CEADs), which provide direct CO_2 emissions from final fossil fuel consumption for the transportation, storage, and postal sectors, explicitly excluding indirect emissions from electricity or heat production [19]. Therefore, these emissions data represent direct final energy consumption, inherently excluding efficiency losses related to electricity generation, transmission, distribution, or vehicle charging processes.

Comment R3.2:

Introduction:

The research question should be presented more explicitly. After the section addressing the knowledge gap, it would strengthen the manuscript to outline its structure. This is especially important given the unconventional order of chapters in the current version.

Response to Comment R3.2:

Thank you for this helpful suggestion. We agree that explicitly stating the research question and clearly outlining the manuscript structure will strengthen the clarity and readability of our paper, particularly given its somewhat unconventional organization.

Accordingly, we have revised the end of the Introduction section to explicitly present our research question, directly following the identified knowledge gap. Additionally, we now provide a concise overview of the manuscript's structure, clearly describing the content and purpose of each subsequent section to guide the reader smoothly through the paper.

These revisions appear in the updated Introduction section of the manuscript, shown as follow.

In this context, this study explicitly asks: *What is the nationwide deployable capacity and carbon reduction potential of roadside PV systems along China's transportation corridors, and how does this potential vary spatially across regions?* To address this question, we propose a high-resolution spatiotemporal methodology that integrates meteorological data with transportation layouts on the basis of precise geographic information

…

The remainder of this paper is organized as follows: In Section 2, we first introduce our analytical framework and spatial matching approach, and then quantify the national and regional potential of roadside PV deployment. After comparing results derived from our high-resolution method against traditional aggregated methods, we explore how large-scale roadside PV deployment may impact regional net-load patterns and power system operation. We then estimate regional carbon mitigation potentials and further examine how system configuration choices (e.g., tracking methods and tilt angles) influence PV generation outcomes. In Section 3

and 4, we synthesize the key insights from our analysis, discuss their broader policy and planning implications, acknowledge study limitations, and outline avenues for future research. Finally, Section 5 provides comprehensive details on data, computational methods, and validation approaches, with additional sensitivity analyses, parameters, and datasets available in the Supplementary Information.

Comment R3.3:

Formatting:

Line 121: indentation requires correction.

More broadly, indentation and formatting should be revised throughout the manuscript for consistency.

Response to Comment R3.3:

Thank you for your suggestion. We have carefully checked and corrected the indentation issue at line 121. Furthermore, we have thoroughly reviewed and revised the manuscript to ensure consistent indentation and formatting throughout, aligning fully with the journal's formatting guidelines.

Comment R3.4:

Equation 1:

The equation needs to be explained in more detail.

The authors should reference scientific literature that has adopted a similar strategy.

Why was 10 MW chosen as the reference size?

Have safety or correction coefficients been applied to account for module unavailability, distances between modules, and other practical constraints?

Response to Comment R3.4:

Thank you for raising these important questions. Below, we provide detailed clarifications addressing each of your concerns regarding Equation (1):

1) Explanation of Equation (1) and Reference Source:

Equation (1) used in our study is based on the industry standard issued by China's Ministry of Land and Resources, specifically the document titled *"Land quota of photovoltaic power station project" (TD/T 1075-2023)* [20]. This official guideline provides a standard method to calculate land use area for PV installations, explicitly taking a 10 MW PV installation as the reference unit and specifying:

Land use area = Land area for 10 MW PV array × (Actual installed capacity / 10 MW)

Thus, the 10 MW size is used purely as a standardized reference point, rather than as a

constraint or explicit requirement on actual project scale. Its role is to provide a unified and scalable benchmark suitable for PV projects of varying capacities.

2) Justification for the Selection of the 10 MW Reference Size:

The use of a 10 MW reference scale aligns with common engineering practices and national land-management standards in China. It is widely adopted by policymakers and planners to simplify land allocation, permit comparison across projects, and facilitate streamlined approvals. This reference size is not arbitrary, but a practical convention commonly used in planning and regulatory documentation.

3) Inclusion of Safety or Correction Coefficients:

We acknowledge your important point regarding practical constraints such as module spacing, access paths, and module unavailability. However, these real-world constraints have already been implicitly accounted for within the standardized land-use indicators provided by the referenced governmental guideline. Specifically, the guideline's reference land area inherently includes standard assumptions for inter-module spacing, maintenance access, shading losses, and typical module availability constraints.

Given that the standardized reference already incorporates these practical factors, we did not separately introduce additional explicit correction coefficients. Furthermore, we recognize that roadside PV systems might differ slightly in layout from traditional ground-mounted PV plants. Nevertheless, our study focuses on assessing realistic deployment potential at a national scale, adhering explicitly to established regulatory guidelines to ensure practical feasibility and policy compliance, rather than detailed modeling of spatial configurations.

To clarify these points explicitly, we have added a detailed explanation immediately following Equation (1) in the revised Methods section.

Equation (1) is derived from the Chinese Ministry of Land and Resources' official industry standard *"Land quota of photovoltaic power station project" (TD/T 1075-2023)* [20], which uses a 10 MW PV installation as a reference unit to standardize land-use calculations across different project scales. Thus, the choice of a 10 MW reference capacity is intended solely as a standard benchmark rather than a limitation on actual installed capacities. Moreover, the reference land-area parameter inherently accounts for practical engineering constraints, including module spacing, maintenance paths, shading losses, and module availability. As such, additional explicit correction coefficients were not separately introduced in this study. This approach ensures realistic, feasible, and policy-compliant estimations of roadside PV deployment potential without necessitating detailed modeling of specific spatial arrangements.

Comment R3.5:

Scenario Configurations (Line 639):

The definition of scenarios should be supported by literature references or additional discussion.

Why were these specific scenarios chosen?

Were other potential scenarios discarded, and if so, for what reasons?

Response to Comment R3.5:

Thank you very much for raising these insightful points, which have helped us to clarify the rationale and robustness of our scenario definitions.

When constructing the scenario configurations, we primarily considered two key dimensions: available land resources and available solar resources, as these two dimensions fundamentally determine the output of roadside photovoltaic systems.

1) Land resource dimension: Available land for roadside PV deployment primarily depends on the physical width available alongside transportation corridors. Based on the typical cross-sectional characteristics of Chinese roads, we differentiated available roadside widths for railways and highways (including expressways, national roads, and provincial roads) into three representative scenarios—conservative, moderate, and optimistic—to reflect the variety and complexity of road infrastructure across China. This approach aims to capture realistic and diverse deployment conditions, ensuring the broad applicability and practical relevance of our analysis.

2) Solar resource dimension: Effective utilization of solar resources for roadside PV systems is strongly influenced by tracking methods and tilt angles. Thus, we incorporated multiple tracking configurations (fixed-tilt, single-axis tracking, and dual-axis tracking) and tilt-angle variations (optimal tilt angle ±10°) into our scenarios to comprehensively evaluate their impact on actual power generation. All chosen parameters for tracking methods and tilt angles were derived from published literature and international PV technology reports, ensuring the realism and credibility of our assumptions.

Furthermore, we recognized that PV installation capacity per unit land area is also influenced by PV module efficiency and tracking configurations under national land-use regulations. Thus, based on China's *"Land quota of photovoltaic power station project"* and *"Specification of photovoltaic power generation efficiency"*, combined with recent development trends in monocrystalline silicon PV module efficiency (covering both laboratory benchmarks and commercial applications), we defined conservative (20%), moderate (24%), and optimistic (28%) efficiency scenarios. These scenarios reflect mainstream commercial PV technologies currently available on the market.

The scenarios we established therefore reflect explicit research logic and multi-dimensional practical engineering considerations, clearly justifying why these specific scenarios were selected. To further clarify, we categorized the influencing factors according to their main roles:

1) Factors affecting PV installed capacity: PV tracking methods, roadside deployment

widths, and module efficiency.

2) **Factors affecting PV capacity factors:** PV tracking methods and tilt angles.

We have explicitly detailed the parameter settings, underlying assumptions, and specific numerical values for each scenario in the Supplementary Information (SI). Relevant supporting literature—including national road engineering standards, trends in monocrystalline silicon module efficiencies, and national PV efficiency guidelines—are clearly referenced in the SI tables, ensuring the rigor and transparency of our methodology.

Additionally, as you noted, we did consider other potentially optimistic scenarios (such as ultra-high efficiency modules exceeding 30%, or experimental tracking technologies). However, these were excluded from our analysis because they currently lack widespread commercial feasibility or do not align well with realistic roadside deployment conditions. Thus, the selected scenarios reflect a careful balance of practical operability, technological maturity, and policy compliance, and provide an inclusive set of reference configurations adaptable to varying geographical, policy, and economic contexts across China.

Importantly, to ensure transparency and methodological rigor, we have provided detailed scenario definitions, comprehensive parameter tables, numerical assumptions, and specific supporting literature references explicitly in the Supplementary Information (**Supplementary Note 3: Scenario Settings and Parameter Matrix**).

In response to your comment, we have significantly expanded the description of our scenario logic and justification in the revised Methods section

Scenario Configurations

We designed a comprehensive set of scenarios to capture realistic variations in roadside PV deployment potential across China. The scenarios systematically considered three critical dimensions influencing PV output: **available roadside land resources**, **PV tracking configurations and tilt angles**, and **module efficiency levels**.

1) Available roadside land resources were classified into conservative, moderate, and optimistic scenarios based on typical cross-sectional widths of transportation corridors, including railways and various classes of highways (expressways, national roads, provincial roads) [21][22].

2) Tracking configurations included fixed-tilt, single-axis, and dual-axis tracking methods, reflecting commercially available technologies. For tilt angles, we considered optimal angles and variations of ±10° to capture realistic installation conditions [23].

3) Module efficiency levels were set at conservative (20%), moderate (24%), and optimistic (28%) values based on current mainstream commercial monocrystalline silicon PV modules and national technical standards [24][25].

Combining these dimensions yielded a total of 27 installation scenarios and 63 generation scenarios, systematically covering a broad range of potential deployment outcomes. Detailed

parameter settings, numerical values, and specific scenario definitions (including representative minimum and maximum cases) are provided in the Supplementary Information (SI §S3).

Comment R3.6:

Grid Infrastructure Considerations:

It is not clear how the authors accounted for grid connection feasibility. Even with sufficient space and favorable irradiance, very remote areas may lack grid access, especially at the required connection capacities. The authors should clarify how such infrastructure limitations were addressed in the study.

Response to Comment R3.6:

Thank you very much for highlighting the critical issue regarding grid infrastructure feasibility. To clarify, this study focuses on evaluating the technical potential and carbon reduction benefits of roadside photovoltaic (PV) deployment at a national scale. We did not explicitly incorporate practical grid-connection constraints (such as substation proximity, feeder capacity limitations, voltage-level compatibility, or grid reinforcement requirements), primarily due to the limited availability of detailed nationwide geospatial grid infrastructure data and the substantial increase in complexity such detailed constraints would impose on a national-scale analysis.

We fully acknowledge that grid infrastructure constraints significantly influence the practical deployment feasibility and economic viability of roadside PV systems. Thus, actual project-level deployments will require rigorous, site-specific feasibility assessments that explicitly consider proximity to existing substations, available feeder capacities, voltage levels, and the potential costs associated with grid upgrades or extensions.

In the revised manuscript, we explicitly clarified this methodological scope at the end of Methods Section 5.6 by adding a dedicated paragraph ("Grid infrastructure considerations"), clearly outlining the distinction between the technical potential assessed in our study and the additional feasibility analyses necessary for practical implementation.

The specific revisions are as follows:

…

In addition to these data-related uncertainties, practical deployment of roadside PV systems also involves critical considerations regarding grid connection feasibility.

Grid infrastructure considerations: This study assesses roadside photovoltaic deployment primarily from the perspective of technical potential and system impacts, without explicitly incorporating grid-connection constraints such as proximity to substations, feeder capacity limitations, or required voltage levels. Consequently, the estimated results represent theoretical or technical potential, which may differ from practical or economically feasible deployment

scales due to existing grid infrastructure limitations. For practical implementation, detailed project-level analyses will be required to assess grid access conditions, including the spatial proximity of roads to existing substations and transmission lines, available substation and feeder headroom, and potential grid upgrade costs. An illustrative economic evaluation framework, detailing investment cost categories and parametric estimation methods, is provided in Supplementary Note 6.

Comment R3.7:

Line 340:

The terms "peak-to-valley differences" and "fluctuation mileage" should be better defined.

The inclusion of equations would improve clarity.

It is not immediately evident how the load profile is incorporated into the analysis—this should be explained in detail.

Response to Comment R3.7:

Thank you for highlighting this point. We agree that providing clear explanations of the selected metrics and explicitly describing how the load profile enters the analysis will significantly improve readability and clarity.

The two metrics, **daily peak-to-valley differences (PTV)** and **daily fluctuation mileage (MIL)**, were chosen because they reflect complementary aspects of power system flexibility challenges associated with variable PV generation. Specifically, **PTV** quantifies the maximum daily range between peak and minimum net load, thus indicating the magnitude of intra-day operational adjustments required. Meanwhile, **MIL** measures the cumulative magnitude of hourly load ramps within a day, effectively capturing the total operational burden placed on dispatchable generation and storage resources. Both metrics are calculated directly based on hourly net load profiles, defined explicitly as the regional hourly electricity demand minus the corresponding roadside PV generation, thereby clearly demonstrating how load profiles are integrated into our analysis.

In line with your suggestion, we have clarified the definitions and relevance of these two metrics at the beginning of Section 2.4. Specifically, we revised the section as follows:

To evaluate the impact of roadside PV deployment on the net load curves of power systems, this study explores the temporal characteristics of PV generation and their implications for the power system's supply-demand balance. The net load, defined as the hourly regional electricity demand minus the hourly PV generation, directly affects the flexibility requirements of the grid, influencing the operational demands for resources such as energy storage and thermal power

plants, as well as shaping the economic efficiency and integration capacity of PV systems. Specifically, two metrics are introduced to capture key aspects of the net load profile:

1) Daily peak-to-valley differences (PTV) quantify the range between the highest and lowest hourly net load within a day, reflecting the required daily ramping capacity and indicating the maximum intra-day operational stress on grid-balancing resources.

2) Daily fluctuation mileage (MIL) measures the cumulative absolute hourly ramps of net load over a day, capturing the overall variability and frequency of adjustments required from dispatchable resources, thus reflecting the intensity of operational flexibility demands.

These two metrics are chosen because they comprehensively characterize the operational challenges imposed by variable renewable generation on power system operation—both the magnitude of short-term fluctuations (PTV) and the total flexibility burden (MIL). The regional load profiles enter the analysis explicitly through the hourly calculation of net load, combining spatially resolved PV output data with actual hourly demand patterns. **A detailed description of how these metrics are computed is provided in the Methods section (Section 5.5)**. Fig. 6 illustrates the changes in these two metrics across different levels of roadside PV deployment and regional contexts, highlighting substantial variability in flexibility requirements driven by regional load and resource characteristics.

5.5 Impact of Roadside PV on Net Load Curves: Calculation Methods

Six PV deployment scenarios were defined (0%, 20%, 40%, 60%, 80%, and 100%), and hourly load and generation data were used to calculate the daily net load peak–valley difference (PTV) and daily fluctuation mileage (MIL) for each scenario. To further analyse the variations across scenarios, the differences in these two metrics between the deployment scenarios (20%, 40%, 60%, 80%, and 100%) and the baseline scenario (0% deployment) were calculated. The formulas are detailed below:

$$\begin{cases} PTV_{i,D} = max(L_{i,D,t}) - min(L_{i,D,t}) \\ MIL_{i,D} = \sum_{t=1}^{23} |L_{i,D,t+1} - L_{i,D,t}| \\ i = 0,1 \dots ,5; \ D = 1,2 \dots ,365; \ t = 1,2 \dots ,24 \end{cases} \tag{1.}$$

where $PTV_{i,D}$ (in GW) and $MIL_{i,D}$ (in GWh) denote the daily net load peak-to-valley difference and daily fluctuation mileage, respectively. $L_{i,D,t}$ denotes the net load for scenario i at hour t on day D.

The differences in the two metrics between the deployment scenarios and the baseline are calculated as follows:

$$\begin{cases} PTV_{i-0,D} = PTV_{i,D} - PTV_{0,D} \ \mathrm{i} = 1,2 \dots ,5 \\ MIL_{i-0,D} = MIL_{i,D} - MIL_{0,D} \ \mathrm{i} = 1,2 \dots ,5 \end{cases} \tag{2.}$$

where $PTV_{i-0,D}$ and $MIL_{i-0,D}$ represent the differences in the daily net load peak-to-valley

difference and thermal power adjustment mileage between scenario i and the baseline scenario (0% deployment) for day D.

Comment R3.8:

Line 414:

The current phrasing implies causality. Since correlation does not imply causation, the sentence should be revised accordingly.

Response to Comment R3.8:

Thank you for pointing out this issue. We fully agree that correlation alone does not imply causation, and our previous wording (page 15, line 414) may inadvertently suggest a causal relationship. In the revised manuscript, we have carefully adjusted the phrasing to emphasize correlation and association rather than causality.

The specific revision in Section 2.5 is as follows:

…

Correlation analysis indicates a strong positive association between PV generation potential and carbon reduction ($R^2 > 0.8$), especially in resource-rich northern provinces. Baseline transportation emissions show moderate correlation ($R^2 \approx 0.5$), whereas solar irradiance, though having weaker correlation overall ($R^2 \approx 0.3$), is particularly influential in low-emission regions, such as Northwest China, where high insolation is associated with substantial carbon mitigation even at modest-scale PV deployment. These findings highlight the value of region-specific carbon accounting: in high-emission, infrastructure-dense provinces, PV can serve as a bulk offset mechanism, while in resource-rich but low-demand areas, it represents a targeted decarbonization option with high marginal value.

Comment R3.9:

Economic Considerations:

The manuscript would benefit significantly from an economic estimation of the required investments, both for power generation and the associated grid infrastructure. Even a rough estimate would provide valuable context on the feasibility and scale of the proposed solutions.

Response to Comment R3.9:

Thank you very much for highlighting the importance of providing an economic context to clarify the feasibility and scale of the proposed roadside PV deployment. We fully agree that even rough economic estimations can substantially enhance readers' understanding of deployment practicality.

To explicitly address your suggestion, we have added a transparent, structured economic

evaluation framework to the revised **Supplementary Information (Supplementary Note 6)**. Specifically, we have clearly distinguished three categories of investment costs for roadside PV deployment: **1) PV equipment costs:** including modules, mounting structures, foundational electrical components (such as basic inverter and wiring), and civil engineering costs. **2) Technical adaptation costs:** specifically required for roadside PV installations, including DC–DC voltage step-up equipment, grid-side metering, monitoring, protection equipment, and dedicated electrical connection infrastructure. **3) Grid reinforcement costs:** covering grid-side investments necessary to accommodate newly added roadside PV capacity, including distribution feeder upgrades, transformer expansions, and potential line extensions or new grid construction.

In the revised SI part, we provide clear and transparent parametric formulas, recommended reference cost ranges, and illustrative figures. We also explicitly clarify the methodological limitations and boundaries of this economic evaluation approach, emphasizing that it provides order-of-magnitude estimations suitable for comparative scenario analyses rather than detailed project-level feasibility assessments.

Detailed descriptions and parameter explanations are presented clearly in Supplementary Note 6 of the revised manuscript, shown as follows.

Investment cost estimation method for roadside PV projects

To clearly evaluate the investment scale and economic feasibility of roadside photovoltaic projects, we have developed a simple and transparent parametric cost estimation framework. The costs are explicitly divided into three main categories:

1) PV equipment cost, C_{pv}

This includes all investment related to PV system hardware and construction, such as PV modules, mounting structures, foundational electrical components (e.g., basic inverter and DC-side wiring), and site installation (excluding grid connection or reinforcement costs). Based on authoritative industry references (IEA PVPS and IRENA), we propose a reasonable cost range of approximately 0.50–0.80 USD/W [26][27].

2) Technical adaptation and interconnection cost, C_{ta}

This covers additional technical adaptation and interconnection infrastructure specifically required for roadside PV installations, such as DC–DC voltage step-up equipment or transformers, metering, protection, monitoring systems, and dedicated AC-side grid interconnection equipment. According to the NREL 2023 cost benchmarking report, electrical balance-of-system (BOS/EBOS) and inverter components significantly contribute to system costs and have recently exhibited an increasing trend. Considering additional roadside-specific interface adaptations, we estimate this incremental cost at approximately 5–15% of system costs, equivalent to 0.03–0.10 USD/W [28]. Note this category is distinct from (1), as it specifically covers interface adaptation rather than primary PV equipment.

3) Grid reinforcement cost, C_{grid}

This refers to the additional costs incurred by the grid to accommodate new roadside PV capacity, including feeder reinforcement (conductors, voltage regulation, protective relays), transformer expansions, and new or extended grid lines. The calculation method is as follows:

$$C_{\text{grid}} = k \cdot P_{pv} \cdot C_g \tag{6}$$

where the expansion factor k is defined as:

$$k = \frac{\max(P_{pv} - H,\ 0)}{P_{pv}} \tag{7}$$

Here P_{pv} represents the planned installed PV capacity (MW), and $\boldsymbol{H}$ represents the current grid hosting capacity (MW). The expansion factor k directly quantifies the proportion of additional grid reinforcement required. Specifically, k=0 means no grid reinforcement is needed, providing the easiest and most economical scenario. In contrast, k=100% indicates no available grid hosting capacity, thus requiring maximum grid reinforcement investment. We adopt a conservative unit grid reinforcement cost range of approximately 0.15–0.50 USD/W, considering typical international benchmarks for urban distribution network upgrades [29].

Thus, the total investment cost is expressed as:

$$C_{\text{total}} = C_{pv} + C_{ta} + C_{grid} \tag{8}$$

The figure below presents the upper and lower boundaries of total investment costs for roadside PV deployment at varying grid expansion factors k, reflecting different levels of grid reinforcement requirements. Total investment cost is explicitly divided into three categories: 1) PV equipment costs, 2) technical adaptation and interconnection costs, and 3) grid reinforcement costs. The shaded bands indicate the ranges of total investment, reflecting uncertainties across individual cost components.

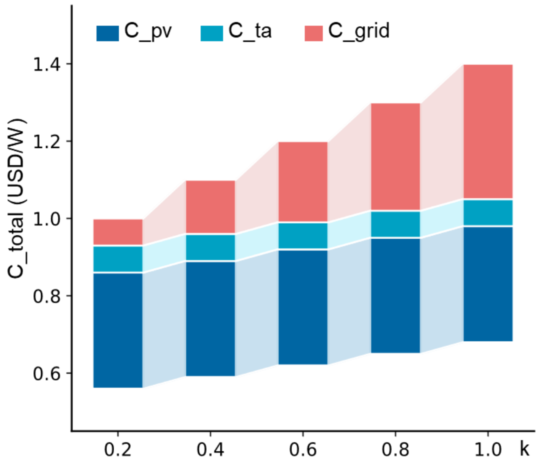

Fig. S15 Estimated investment cost ranges of roadside PV projects under varying grid expansion scenarios

It should be emphasized that this cost estimation framework is designed to provide order-of-magnitude estimates and comparative scenario evaluations, clarifying economic scale and

feasibility under different deployment conditions. However, it is based on linear simplifications of grid reinforcement costs, which in practice may have nonlinear or step-wise cost characteristics, especially when involving substation expansions or new grid construction. Furthermore, estimates of hosting capacity H are based on typical feeder limits or regional load characteristics as conservative proxies, which may differ from actual grid conditions influenced by real network structures and operational strategies. The unit cost ranges (PV equipment, technical adaptation, grid reinforcement) are based on typical industry references and may fluctuate according to specific market conditions, construction practices, and local grid characteristics. The scope of this cost estimation framework is explicitly limited to initial capital investments, excluding taxation, financing, or project financial returns (such as LCOE or IRR). Therefore, while the framework provides useful national-scale technical-economic potential analyses and scenario comparisons, detailed feasibility studies based on actual local conditions, engineering designs, and specific grid data should be performed prior to real-world implementations.

[1] Sparks, A. H. nasapower: a NASA POWER global meteorology, surface solar energy and climatology data client for R. Journal of Open Source Software 3, 1035 (2018).

[2] Hersbach, H. et al. The ERA5 global reanalysis. Quarterly Journal of the Royal Meteorological Society 146, 1999–2049 (2020).

[3] Sengupta, M. et al. The National Solar Radiation Data Base (NSRDB). Renewable and Sustainable Energy Reviews 89, 51–60 (2018).

[4] Bessho, K. et al. (2016). An Introduction to Himawari-8/9—Japan's New-Generation Geostationary Meteorological Satellites. Journal of the Meteorological Society of Japan, 94, 151–183.

[5] The State Council of the People's Republic of China. *Action Plan for Energy Conservation and Carbon Reduction (2024–2025).* (2024). Available at the State Council portal.

[6] Ministry of Transport of the People's Republic of China. *Guiding opinions on promoting the integration of transportation and energy* (news/policy release, Apr 2025). Ministry of Transport official website.

[7] National Development and Reform Commission & National Energy Administration. *Notice on promoting the orderly development of green-power direct connection* (May 2025). NDRC official website.

[8] Rijkswaterstaat (Netherlands). *Solar Highways—From motorway noise to clean energy* (project page).

[9] European Commission, LIFE Programme. *LIFE13-ENV/NL-000971 "Solar Highways"* (project database entry).

[10] Federal Highway Administration (FHWA). *Right-of-Way Use Agreements; 23 CFR §710.405* (current eCFR).

[11] FHWA—Value Capture. *Massachusetts Right-of-Way Solar Program (Case Study).*

[12] Massachusetts Department of Transportation (MassDOT). *Renewable Energy Projects—Highway ROW Solar Program.*

[13] ASTRA (Swiss Federal Roads Office). *Photovoltaics on National Roads—Programme/Call overview.*

[14] ASTRA. *Roads and Traffic 2023/2024—Developments, Facts and Figures* (2024).

[15] ASFINAG / IÖB-Innovationsplattform. *Photovoltaics on Noise Barriers—Challenge (English brief).*

[16] Waqas, H., Jiang, Y., Shang, J., Munir, I. & Khan, F.U. An integrated approach for 3D solar potential assessment at the city scale. *Remote Sensing* 15, 5616 (2023).

[17] Waqas, H., Shang, J., Munir, I., Ullah, S., Khan, R., Tayyab, M., Mousa, B. G., & Williams, S. Enhancement of the energy performance of an existing building using a parametric approach. *J. Energy Eng.* 149, 04022057 (2023)

[18] Mooney, P. & Minghini, M. A review of OpenStreetMap data. Mapping and the Citizen Sensor 37–59 (2017)

[19] Xu J, Guan Y, Oldfield J, et al. China carbon emission accounts 2020-2021. Applied Energy, 2024, 360: 122837.

[20] Ministry of Natural Resources of the People's Republic of China. Land quota of photovoltaic power station project. (TD/T 1075-2023) (Ministry of Natural Resources of the People's Republic of China, 2023).

[21] Ministry of Transport of the People's Republic of China. Technical Standards for Highway Engineering (JTG B01-2014) (Ministry of Transport of the People's Republic of China, 2014).

[22] National Railway Administration of the People's Republic of China. Code for Geotechnical Testing of Railway Engineering (TB 10098-2017) (National Railway Administration of the People's Republic of China, 2017).

[23] Nsengiyumva, W. *et al.* Recent advancements and challenges in Solar Tracking Systems (STS): A review. *Renew. Sustain. Energy Rev.* **81**, 250–279 (2018).

[24] National Standard of the People's Republic of China. Specification of photovoltaic power generation efficiency (GB/T 39857-2021) (National Standard of the People's Republic of China, 2021).

[25] Green, M. A. Silicon solar cells step up. Nat. Energy 8, 783–784 (2023).

[26] IEA PVPS. Snapshot of Global Photovoltaic Markets 2024. IEA Photovoltaic Power Systems Programme (2024). https://iea-pvps.org

[27] IRENA. Renewable Power Generation Costs in 2023. International Renewable Energy Agency (2024). https://www.irena.org

[28] Feldman, D., Ramasamy, V., Fu, R., Ramdas, A. & Margolis, R. U.S. Solar Photovoltaic System and Energy Storage Cost Benchmark: Q1 2023. National Renewable Energy Laboratory (NREL), NREL/TP-6A20-86891 (2023). https://www.nrel.gov

[29] EPRI. Hosting Capacity—A Summary of Methods, Practical Considerations, and Future Research. Electric Power Research Institute, 3002011008 (2019). https://www.epri.com

Manuscript: NCOMMS-25-57254-A

Authors: Zhaoyuan Wu, Jianxiao Wang, Lanyi Wei, Bo Li, Lin Chen, Lu Zhang, Daniel M. Kammen, Gengyin Li, Ming Zhou, Jie Song

Title: High-resolution national assessment of infrastructure coupled roadside solar energy toward transportation decarbonization

Response to Reviewers

We thank the Reviewers for their careful evaluation of our manuscript and for the constructive comments. We have revised the manuscript and Supplementary Information accordingly and believe that we have addressed all concerns raised. Below, the reviewers' comments are reproduced in italic and numbered for clarity, followed by our point-by-point responses. All changes in the revised manuscript are highlighted in **Blue**. Point-by-point responses to comments from the two reviewers are provided below.

Reviewer 1 Comments to the Authors

The manuscript has improved a lot after the previous round of revisions. However, regarding the validation of the meteorological data resolution (specifically related to Comments R1.2 and R2.2), one critical issue remains unresolved that necessitates further clarification before publication.

Comment R1.1:

While the authors have provided a sensitivity analysis in Supplementary Note 5.3 demonstrating that estimation errors increase as grid resolution coarsens from 0.5° to 17°, the current validation remains incomplete. The analysis treats the 0.5° NASA POWER dataset as a baseline without quantifying the potential deviation of this baseline itself.

Given the authors' demonstration that "estimation errors systematically increase as the meteorological grid resolution becomes coarser", it is logically inferred that the 0.5° resolution may still retain non-negligible biases compared to finer-scale data (e.g., <0.1°), particularly in regions with complex terrain or high infrastructure density. The justification relying solely on "computational efficiency" and "temporal continuity" is insufficient to dismiss the need for accuracy benchmarking at the sub-0.5 ° scale.

To ensure the robustness of the national-scale assessment, the authors should quantify the uncertainty of their 0.5° baseline. This does not require re-running the entire national model, but a comparative validation against a higher-resolution dataset (e.g., ERA5 at ~0.25° or satellite-derived irradiance) should be conducted for representative sample regions. This is essential to demonstrate that the 0.5° resolution provides a convergent and reliable estimate rather than merely a computationally convenient one.

Response to Comment R1.1:

We sincerely thank you for highlighting this critical and technically important issue.

We agree that, for a national-scale assessment using a **0.5° meteorological grid**, it is necessary to further verify the **convergence and reliability** of this resolution setting against finer-resolution references, and to quantify the uncertainty introduced by the **0.5° spatial approximation**.

To address this concern, we added an additional resolution-benchmarking analysis based on **ERA5 reanalysis in the revised manuscript (Supplementary Note 5.3 Sensitivity analysis of provincial PV generation errors with varying meteorological grid resolution)**.

We note that the NASA POWER meteorological product used in the main analysis is publicly available at a maximum spatial resolution of **0.5°**. Therefore, to further examine the impact of spatial resolution approximation at sub-0.5° scales and to test whether the **0.5°** setting already lies in a **convergent regime**, we used **ERA5** as an example and compared two resolution cases: **0.25°×0.25°** (native ERA5) versus **0.5°×0.5°** (consistently downsampled from the 0.25° fields). Specifically, ERA5 provides meteorological variables at 0.25°×0.25° resolution. To maintain full consistency with our main framework, we extracted the same key inputs and applied an identical PV resource-to-generation conversion workflow. Within the ERA5 dataset, we constructed two resolution settings: (1) directly using the native **0.25°** grid; and (2) generating a corresponding **0.5°** grid through consistent downsampling/aggregation of the 0.25° fields. Solar position parameters in both cases were produced using the same method (**pvlib**) to avoid introducing any confounding differences unrelated to resolution. Under identical data definitions and computational procedures, we therefore isolated and quantified the effect of spatial resolution alone. We computed roadside PV annual generation potentials under both resolution settings and aggregated them to the provincial level. The 0.25° case involves **15,335** grid points, whereas the 0.5° case involves **3,995** grid points. The provincial estimates and their relative differences are summarized in **Fig. S15**.

Building upon the coarse-resolution sensitivity results in Fig. S14, where estimation errors increase markedly as the meteorological grid becomes progressively coarser, with region-level responses differing across China (e.g., relatively stable errors in Southern China (S) but more volatile patterns in Central China (C), and heightened sensitivity for small or spatially heterogeneous provinces), the results in Fig. S15 show that provincial annual generation estimates are highly consistent between the two resolutions. The maximum absolute relative difference is **0.2476%** (XZ). Across all provinces, the **mean** and **median** absolute relative differences are **0.0778%** and **0.0792%**, respectively, with the **95th percentile** at approximately **0.204%**; moreover, **58.1%** of provinces exhibit an absolute relative difference below **0.1%**. At the national aggregate level, the total annual generation differs by only **~0.0205%** between the two resolution settings, indicating that the resolution approximation does not materially affect our national-scale conclusions.

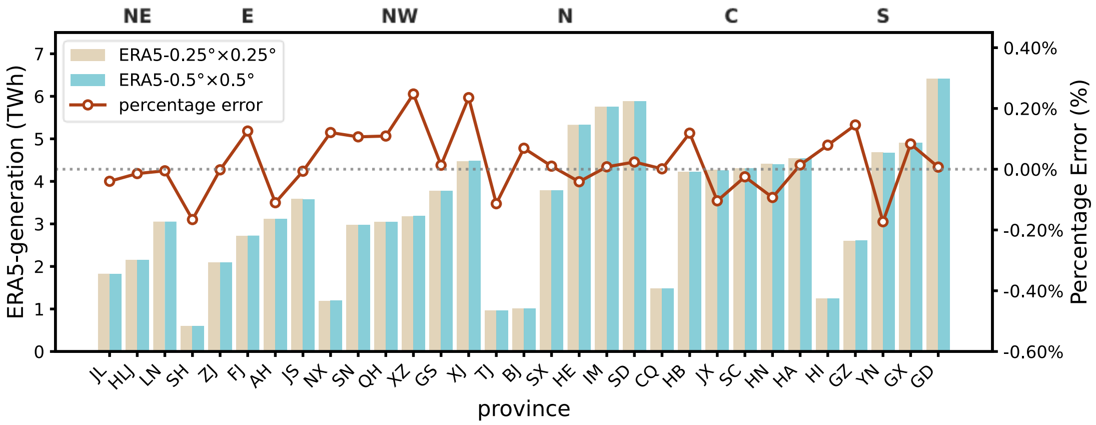

Fig. S15 | Impact of meteorological grid resolution on estimated PV generation potential. The bar charts show provincial annual PV generation potentials computed using ERA5 meteorological inputs at two spatial resolutions (0.25° × 0.25° and 0.5° × 0.5°). The line indicates the relative error (percentage) between the two estimates.

Importantly, the differences between the two resolution settings exhibit clear regional structure, consistent with the regional sensitivity patterns discussed for Fig. S14. For the "0.25° vs 0.5°" comparison, the average absolute relative differences by macro-region are **NE: 0.020%**, **N: 0.044%**, **C: 0.059%**, **E: 0.082%**, **S: 0.098%**, and **NW: 0.139%**. The Northwest (NW) shows the largest sensitivity even at this scale (with the largest provincial deviations occurring in XZ and XJ), consistent with its large latitudinal span and stronger spatial gradients in solar resource conditions. In contrast, Northeast China (NE) exhibits the smallest differences, reflecting comparatively smoother spatial fields at the provincial annual aggregation level. Notably, when grid resolution becomes very coarse, errors can be substantially amplified by the reduced number and representativeness of grid points, particularly for smaller provinces and provinces with highly heterogeneous infrastructure distributions, which helps explain the pronounced volatility observed for certain regions (e.g., C) in Fig. S14. Collectively, Fig. S14 and Fig. S15 jointly demonstrate that (i) excessively coarse meteorological grids can induce substantial provincial-level distortions, but (ii) the **0.5°** setting used in this study already lies in the **convergent regime** for national assessment and provincial annual aggregation, with limited marginal benefit from further refining resolution to **0.25°**.

We have incorporated this additional analysis and the corresponding statistics into **Supplementary Note 5.3 ("Sensitivity analysis of provincial PV generation errors with varying meteorological grid resolutions")**, and added **Fig. S15** to present the provincial comparison and relative error profile for transparent evaluation of the uncertainty range associated with the 0.5° resolution setting.

Reviewer 2 Comments to the Authors

Comment R2.1:

It's better to check with the other reviewer, who is more expert in codes and the running of codes

Response to Comment R2.1:

Thank you for the suggestion.

In line with the journal’s code availability policy, we have uploaded the full source code used in this study, together with the relevant input data and the scripts required to reproduce the key results (including the main figures and selected supplementary figures). The code and data are publicly accessible via the repository link/DOI provided in the revised Code Availability and Data Availability section. We have also added brief reproduction instructions (e.g., environment specifications and step-by-step run commands) to facilitate end-to-end reproducibility.

Comment R2.2:

I have carefully reviewed the revised manuscript and the detailed responses to my previous comments (R2.1–R2.10). I appreciate the authors’ thorough and thoughtful revisions. Overall, the manuscript has been substantially strengthened, and the authors have satisfactorily addressed all major points raised in my initial review.

Response to Previous Methodological and Validation Concerns (R2.2, R2.5)

The authors have convincingly addressed my earlier questions regarding model robustness, sensitivity, and uncertainty. In particular:

• The systematic sensitivity analysis of meteorological grid resolution, now presented in the Supplementary Information (Fig. S14, Table S14), provides strong quantitative evidence supporting the choice of the 0.5° × 0.5° baseline resolution.

• The clarification that the KD-tree approach is a nearest-neighbor matching method rather than a clustering algorithm resolves the ambiguity noted previously.

• The expanded uncertainty quantification and data bias discussion (Methods 5.6) appropriately contextualizes the results as technical upper-bound estimates.

• The explicit documentation of software tools, libraries, and code availability fully addresses reproducibility concerns (codes are not checked yet).

These additions significantly enhance methodological transparency and robustness.

Socio-technical Constraints and Practical Deployability (R2.3, R2.7)

The revised Discussion now clearly and appropriately acknowledges socio-technical barriers, including legal rights-of-way, shading effects, road safety buffers, grid-connection constraints, and economic feasibility. The added discussion on solar design optimization, obstruction modeling, and future site-specific analyses aligns well with my earlier suggestions and strengthens the manuscript’s practical relevance.

Structure and Organization (R2.9)

The manuscript structure has been meaningfully improved. The clear separation of Discussion and Conclusion, and the explicit segmentation into Introduction, Results, Discussion, Conclusion, and Methods, enhances readability and aligns well with Nature Communications conventions (for method see the recommendation).

While the Methods section is placed after the Discussion and Conclusion consistent with journal style. the revised Model Overview (Section 2.1) and clearer cross-referencing now provide sufficient conceptual grounding for interpreting the Results. This adequately resolves my earlier concern regarding structural clarity.

Figures, Tables, and Accessibility (R2.6)

The adoption of colorblind-safe palettes and the inclusion of summary tables comparing baseline, moderate, and optimistic scenarios in the Supplementary Information substantially improve accessibility and interpretability.

Overall Assessment

The authors have responded comprehensively and constructively to all points raised in my initial review. The revised manuscript is now methodologically robust, clearly structured, transparent, and well contextualized, while retaining its strong originality and policy relevance.

I have no further substantive concerns.

The authors have responded comprehensively and constructively to all points raised in my initial review. The revised manuscript is now methodologically robust, clearly structured, transparent, and well contextualized, while retaining its strong originality and policy relevance.

I have no further substantive concerns.

Response to Comment R2.2:

We sincerely thank the reviewer for the careful re-evaluation of our revised manuscript and for the very positive assessment. We are pleased that the reviewer finds our revisions thorough and that the manuscript has been substantially strengthened, with the major concerns from the first round satisfactorily addressed.

Comment R2.3:

Please revise again if it's not an issue with journal. The structure and organization of the paper shouldn't be like: Intro, methods, results, discussion and conclusion?

Response to Comment R2.3:

Thank you for the suggestion.

We agree that different manuscript organizations have their own strengths and are often guided by journal-specific conventions. As *Nature Communications* typically places the **Methods** section after the main text, we have maintained this journal-compliant structure and did not relocate the Methods section. To facilitate reading, we have added clear signposting throughout the main text, explicitly directing readers to the relevant **Methods** subsections at the points

where specific methodological details are needed.

Comment R2.4:

Abstract: as from the abstract, I'd say the novelty is real, but it could be made more explicit. Right now, the abstract reads like: "We did a very good, very detailed assessment." Instead of: "We introduce a new way to treat transportation corridors as energy infrastructure."

Suggested strengthened novelty sentence: "This study introduces a scalable, infrastructureaware spatiotemporal framework that treats transportation corridors as linear energy assets, enabling the first nationwide, high-resolution quantification of roadside photovoltaic potential and associated carbon mitigation."

Response to Comment R2.4:

Thank you for the helpful suggestion. We agree that the novelty can be stated more explicitly. We have therefore revised the Abstract to foreground the methodological contribution before summarizing the national-scale results and implications.

The revised abstract is shown as follows:

Integrating renewable energy into land-based transportation infrastructure represents a promising pathway for decarbonization. However, existing studies have not yet systematically quantified the national potential of roadside photovoltaic (PV) deployment in terms of generation capability and the associated carbon reduction, particularly for extensive systems such as highways and railways. Here we introduce a scalable, infrastructure-aware spatiotemporal framework that treats transportation corridors as linear energy assets, enabling a nationwide, high-resolution quantification of roadside PV potential and associated carbon mitigation. By explicitly coupling geospatial road layouts with high-resolution meteorological inputs, rather than relying on coarse geographical partitioning, our approach improves estimation fidelity for extensive corridor systems, by mapping 480,019 km of national railways and highways onto 4,133 meteorological grids. An efficient KD-tree spatial matching algorithm is proposed to balance computational efficiency and estimation accuracy, offering significant advantages over both geographical partitioning methods and computationally intensive point-by-point mapping. The analysis reveals that roadside PV systems in China's transportation corridors could support 40.91–202.84 GW of installable capacity, generating 57.5–239.2 TWh annually across scenarios. This output meets approximately 50.3% of the current electricity demand in the transportation sector and enables an annual carbon reduction of 33.62–143.97 $MtCO_2$, equivalent to 4.89–20.96% of transportation-related emissions. The results further reveal strong spatial heterogeneity: regions such as North China and Central China offer the highest deployment potential because of dense networks and high irradiation, whereas Northwest China, despite its low demand, can act as a solar export hub. Model robustness is supported by sensitivity analyses, and key uncertainties related to spatial data resolution and infrastructure constraints are

acknowledged. These findings offer scientific guidance for regionalized infrastructure planning and the co-optimization of transportation and energy systems.

Comment R2.5:

Revise the whole manuscript point by point including figures text and readiness align with journal format.

Response to Comment R2.5:

Thank you for the kind suggestion.

We have conducted a careful, point-by-point final check of the entire manuscript and Supplementary Information to ensure submission readiness and compliance with *Nature Communications* formatting requirements. This includes revising figure and table texts/captions, verifying consistency of terminology and notation, checking cross-references between the main text, Methods, and Supplementary Information, and correcting any remaining typographical or formatting issues.

Manuscript: NCOMMS-25-57254-B

Authors: Zhaoyuan Wu, Jianxiao Wang, Lanyi Wei, Bo Li, Lin Chen, Lu Zhang, Daniel M. Kammen, Gengyin Li, Ming Zhou, Jie Song

Title: High-resolution national assessment of infrastructure coupled roadside solar energy toward transportation decarbonization

Response to Reviewers

We thank the Reviewers for their careful evaluation of our manuscript and for the constructive comments. We have revised the manuscript and Supplementary Information accordingly and believe that we have addressed all concerns raised. Below, the reviewers' comments are reproduced in italic and numbered for clarity, followed by our point-by-point responses. All changes in the revised manuscript are highlighted in **Blue**. Point-by-point responses to comments from the reviewer are provided below.

Reviewer 1 Comments to the Authors

The authors' ERA5-based analysis suffers from a fundamental logical circularity. By deriving coarse-resolution fields through spatial aggregation of finer-resolution data, the experiment primarily tests the internal mathematical consistency of spatial averaging, rather than the physical representativeness of inherently coarse-resolution datasets. In this design, high-resolution information is effectively leaked into the synthesized coarse fields.

Because the fine-to-coarse aggregation is itself a form of spatial smoothing, the analysis evaluates whether replacing a known fine-scale field with its spatial mean alters the results. This is not equivalent to the original question of whether native coarse-resolution meteorological data are sufficient. The central concern remains whether the original 0.5° dataset (e.g., NASA POWER), by construction, has already lost critical sub-grid variability associated with local topography, cloud processes, or microclimatic conditions.

The reported 0.0205% difference is therefore a predictable outcome of the smoothing-based experimental design and does not constitute evidence that the original 0.5° resolution is adequate. At a minimum, this methodological limitation should be explicitly acknowledged and the associated conclusions moderated. Ideally, validation against independently generated higher-resolution datasets or ground-based observations—particularly in topographically complex regions—would be required to meaningfully assess the magnitude of sub-grid biases.

Comment R1.1:

Response to Comment of Reviewer 1:

We sincerely thank you for the thoughtful critique.

We agree that a within-product resolution comparison in ERA5 (0.25° versus a 0.5° repre-

sentation obtained via re-gridding/subsampling) only provides a resolution-sensitivity reference and, by design, cannot validate the physical representativeness of a native coarse-resolution product such as NASA POWER or its sub-grid biases.

To directly address this concern, in this revision we strengthened the manuscript in three main ways: **1) explicitly acknowledge this methodological limitation** and accordingly moderate the related conclusions; **2) add a nationwide comparison between native NASA POWER and native ERA5** to quantify data-source uncertainty beyond resolution; and **3) expand the discussion of sub-grid variability**, with particular attention to topographically complex regions.

We agree that ground observations or independent high-resolution products would further strengthen sub-grid bias quantification, and we explicitly note this as an important future direction. **We also clarify one technical detail relevant to the "information leakage" concern: the ERA5 0.5° representation used in Fig. S15 is not constructed by averaging four 0.25° cells.** Instead, it is obtained via consistent re-gridding/subsampling from ERA5, which avoids directly injecting fine-scale averages into the 0.5° representation. Nevertheless, we agree that within-ERA5 resolution comparisons alone cannot validate the physical representativeness of a native coarse-resolution product; therefore, we additionally provide the nationwide native NASA POWER (0.5°) vs native ERA5 (0.25°) comparison.

The specific changes are as follows:

1) Explicitly acknowledge this methodological limitation and accordingly moderate the related conclusion in the revised manuscript **(Section 5.6 Uncertainty quantification and potential data biases)**.

Meteorological data resolution and spatial aggregation effects: The NASA POWER meteorological dataset used in this analysis is provided at a spatial resolution of approximately 0.5° (roughly 50 km). This native grid may not fully capture sub-grid variability in solar irradiance driven by local cloud dynamics, topographic shading, or other microclimatic factors, and can therefore introduce local- to regional-scale biases in estimated photovoltaic generation potential. In addition, mapping gridded meteorological fields to linear roadway infrastructure inevitably involves spatial representation and aggregation choices. We evaluate sensitivity of provincial annual estimates to a within-ERA5 resolution comparison (0.25° versus a 0.5° representation obtained via consistent re-gridding/subsampling) and separately report the native NASA POWER–ERA5 comparison (SI Fig. S15). Residual local discrepancies may be more evident in settings with complex terrain, strong spatial gradients in solar resources, or highly heterogeneous road-network density.

…

These factors introduce local-to-regional uncertainties, particularly in complex-terrain set-

tings. We therefore report the sensitivity associated with meteorological data choice and aggregation in SI Fig. S15 and interpret province-level estimates in light of these ranges.

2) We added an additional resolution-benchmarking analysis between native NASA POWER and native ERA5 to quantify data-source uncertainty beyond resolution **in the revised manuscript (Supplementary Note 5.3 Sensitivity analysis of provincial PV generation errors with varying meteorological grid resolution)**.

We note that the NASA POWER meteorological product used in the main analysis is publicly available at a maximum spatial resolution of 0.5°. An important methodological question is therefore whether using this native 0.5° product could materially affect the estimated provincial annual roadside PV generation potentials, either due to grid resolution or due to dataset-specific construction.

To examine the sensitivity of provincial estimates to using the native 0.5° NASA POWER product, we compared provincial annual PV generation potentials computed using **native NASA POWER (0.5°×0.5°)** with those computed using **native ERA5 (0.25°×0.25°)** under an identical downstream pipeline (same variable extraction, PV conversion procedure, and solar-position calculation via pvlib). Figure S15 provides a nationwide sensitivity check on whether a native 0.5° product is sufficiently representative. Using **native ERA5 (0.25°)** as a reference under an identical downstream pipeline, the comparison between **native NASA POWER (0.5°)** and **native ERA5 (0.25°)** shows **percent-level** differences with clear regional structure. As shown in panels (a) and (c), deviations are predominantly negative for many provinces (NASA POWER yielding lower provincial annual PV generation potentials than ERA5), with the national aggregate offset on the order of **a few percent** (panel c, dashed line). Regionally, larger negative offsets are concentrated in **complex-terrain/high-elevation** regions (e.g., Northwest and parts of Central China), reaching approximately **~5%** at the macro-region level, whereas **Northeast China** exhibits a positive offset (panel c). At the provincial level, deviations can approach **~10%** for some provinces (panel a). These patterns suggest that a native 0.5° product may miss sub-grid variability in certain settings, particularly where spatial gradients and terrain complexity are stronger; accordingly, province-level estimates should be interpreted with appropriate caution.

For reference only, Fig. S15 also includes a within-ERA5 resolution comparison between native ERA5 (0.25°) and an ERA5 representation at 0.5° obtained via consistent re-gridding/subsampling (panels (b) and (d)), which contextualizes the marginal impact of grid resolution within a fixed dataset. As expected, this resolution-only comparison yields much smaller deviations at the provincial annual aggregation scale (maximum absolute difference 0.248% and ~60% of provinces below 0.1% in absolute deviation). Consistent with the reviewer's comment, this within-ERA5 resolution comparison is not used to validate the physical adequacy of

the native NASA POWER product; rather, it provides a magnitude reference under a fixed product.

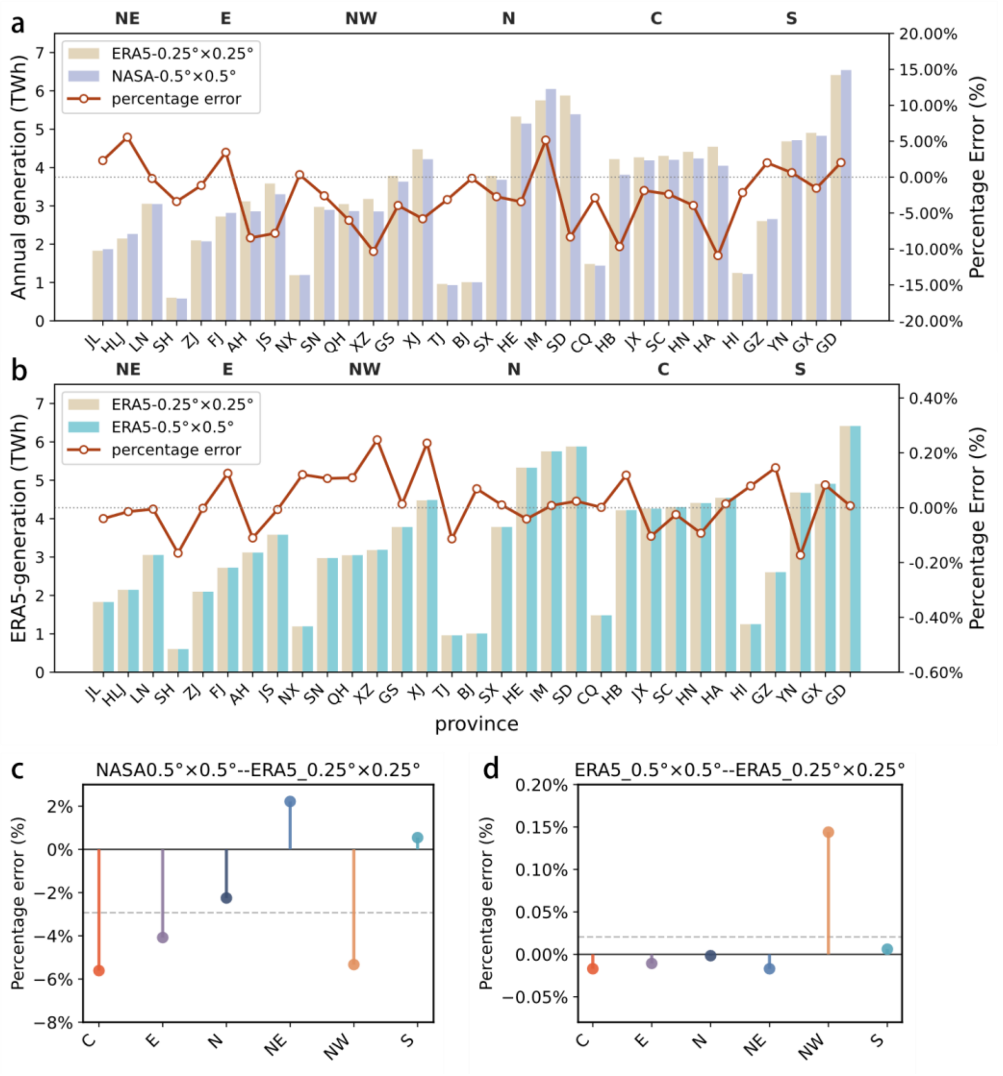

Figure S15 Sensitivity of provincial PV generation estimates to meteorological inputs and resolution coarsening (relative to native ERA5 0.25°). (a–b) Province-level annual roadside PV generation potentials and corresponding relative errors. Bars show provincial annual generation computed using three meteorological inputs: ERA5 0.25°×0.25°, ERA5 0.5°×0.5° (within-ERA5 coarsened setting), and NASA POWER 0.5°×0.5°. The line indicates the relative error (%) of each compared case relative to the ERA5 0.25°×0.25° baseline. (c–d) Macro-region summaries (NE, N, NW, E, C, S) of the relative error (%) for each comparison, where (c) corresponds to NASA POWER 0.5°×0.5° vs ERA5 0.25°×0.25°, and (d) corresponds to ERA5 0.5°×0.5° vs ERA5 0.25°×0.25°. The dashed line indicates the national-scale relative error.

Taken together, Fig. S15 supports two clarifications for interpreting the main analysis. First, the within-ERA5 resolution comparison (0.25° versus an ERA5 0.5° representation obtained via consistent re-gridding/subsampling) provides a resolution-sensitivity context only and does not establish native-dataset adequacy. Second, the native NASA POWER–ERA5 comparison shows that differences associated with native meteorological inputs at 0.5° versus 0.25° can be

material at the provincial annual scale, especially in complex-terrain regions; we therefore disclose this sensitivity and discuss its implications in the revised manuscript.

3) Expand the discussion of sub-grid variability, with particular attention to topographically complex regions in the revised manuscript (Section 3 Discussion), **shown as follows.**

This study contributes to a deeper understanding of how spatial structure, infrastructure form, and solar resource distribution jointly shape the deployment potential and system impact of distributed PV in transportation systems. Three key structural mechanisms emerge. First, pronounced spatial decoupling exists between PV resource availability and transportation electricity demand, driven by the mismatch between solar irradiance gradients and freight activity concentrations. Second, estimation biases in PV potential are systematically linked to spatial aggregation effects, particularly in regions with concentrated or elongated road networks and steep irradiance gradients. Third, the choice of system configuration, such as the tracking method and tilt angle, interacts nonlinearly with local resource conditions, highlighting the need for deployment strategies that reflect both spatial heterogeneity and geometric sensitivity. In addition, sub-grid variability in irradiance can be more pronounced in complex-terrain and high-elevation regions due to terrain-induced gradients and heterogeneous cloud conditions. As a result, province-level estimates should be interpreted with appropriate caution in these settings, and project-level deployment would benefit from higher-resolution irradiance products and site-specific shading assessments.

Second-Round Comments to the Authors

I have carefully reviewed the revised manuscript and the detailed responses to my previous comments (R2.1–R2.10). I appreciate the authors' thorough and thoughtful revisions. Overall, the manuscript has been substantially strengthened, and the authors have satisfactorily addressed all major points raised in my initial review.

Response to Previous Methodological and Validation Concerns (R2.2, R2.5)
The authors have convincingly addressed my earlier questions regarding model robustness, sensitivity, and uncertainty. In particular:
• The systematic sensitivity analysis of meteorological grid resolution, now presented in the Supplementary Information (Fig. S14, Table S14), provides strong quantitative evidence supporting the choice of the 0.5° × 0.5° baseline resolution.
• The clarification that the KD-tree approach is a nearest-neighbor matching method rather than a clustering algorithm resolves the ambiguity noted previously.
• The expanded uncertainty quantification and data bias discussion (Methods 5.6) appropriately contextualizes the results as technical upper-bound estimates.
• The explicit documentation of software tools, libraries, and code availability fully addresses reproducibility concerns (codes are not checked yet).

These additions significantly enhance methodological transparency and robustness.

Socio-technical Constraints and Practical Deployability (R2.3, R2.7)
The revised Discussion now clearly and appropriately acknowledges socio-technical barriers, including legal rights-of-way, shading effects, road safety buffers, grid-connection constraints, and economic feasibility. The added discussion on solar design optimization, obstruction modeling, and future site-specific analyses aligns well with my earlier suggestions and strengthens the manuscript's practical relevance.

Structure and Organization (R2.9)
The manuscript structure has been meaningfully improved. The clear separation of Discussion and Conclusion, and the explicit segmentation into Introduction, Results, Discussion, Conclusion, and Methods, enhances readability and aligns well with Nature Communications conventions (for method see the recommendation).

While the Methods section is placed after the Discussion and Conclusion consistent with journal style. the revised Model Overview (Section 2.1) and clearer cross-referencing now provide sufficient conceptual grounding for interpreting the Results. This adequately resolves my earlier concern regarding structural clarity.

Figures, Tables, and Accessibility (R2.6)
The adoption of colorblind-safe palettes and the inclusion of summary tables comparing

baseline, moderate, and optimistic scenarios in the Supplementary Information substantially improve accessibility and interpretability.

Overall Assessment
The authors have responded comprehensively and constructively to all points raised in my initial review. The revised manuscript is now methodologically robust, clearly structured, transparent, and well contextualized, while retaining its strong originality and policy relevance.

I have no further substantive concerns.

Recommendation:

1. Please revise again if it's not an issue with journal. The structure and organization of the paper shouldn't be like: Intro, methods, results, discussion and conclusion?
2. Abstract: as from the abstract, I'd say the novelty is real, but it could be made more explicit. Right now, the abstract reads like: *"We did a very good, very detailed assessment."* Instead of: *"We introduce a new way to treat transportation corridors as energy infrastructure."*

Suggested strengthened novelty sentence: *"This study introduces a scalable, infrastructure-aware spatiotemporal framework that treats transportation corridors as linear energy assets, enabling the first nationwide, high-resolution quantification of roadside photovoltaic potential and associated carbon mitigation."*

3. Revise the whole manuscript point by point including figures text and readiness align with journal format.

Accept (minor editorial refinements at the editor's discretion).
